# Whole genome sequencing of a banana wild relative *Musa itinerans* provides insights into lineage-specific diversification of the *Musa* genus

**DOI:** 10.1038/srep31586

**Published:** 2016-08-17

**Authors:** Wei Wu, Yu-Lan Yang, Wei-Ming He, Mathieu Rouard, Wei-Ming Li, Meng Xu, Nicolas Roux, Xue-Jun Ge

**Affiliations:** 1 Key Laboratory of Plant Resources Conservation and Sustainable Utilization, South China Botanical Garden, the Chinese Academy of Sciences, Guangzhou 510650, China; 2 BGI-Shenzhen, Shenzhen 518083, China; 3 Bioversity International, Parc Scientifique Agropolis II, 34397 Montpellier Cedex 5, France; 4 Key Laboratory of Tropical Fruit Biology, Ministry of Agriculture, South Subtropical Crops Research Institute, Chinese Academy of Tropical Agricultural Sciences, Zhanjiang 524091, China

## Abstract

Crop wild relatives are valuable resources for future genetic improvement. Here, we report the *de novo* genome assembly of *Musa itinerans*, a disease-resistant wild banana relative in subtropical China. The assembled genome size was 462.1 Mb, covering 75.2% of the genome (615.2Mb) and containing 32, 456 predicted protein-coding genes. Since the approximate divergence around 5.8 million years ago, the genomes of *Musa itinerans* and *Musa acuminata* have shown conserved collinearity. Gene family expansions and contractions enrichment analysis revealed that some pathways were associated with phenotypic or physiological innovations. These include a transition from wood to herbaceous in the ancestral Musaceae, intensification of cold and drought tolerances, and reduced diseases resistance genes for subtropical marginally distributed *Musa* species. Prevalent purifying selection and transposed duplications were found to facilitate the diversification of NBS-encoding gene families for two *Musa* species. The population genome history analysis of *M. itinerans* revealed that the fluctuated population sizes were caused by the Pleistocene climate oscillations, and that the formation of Qiongzhou Strait might facilitate the population downsizing on the isolated Hainan Island about 10.3 Kya. The qualified assembly of the *M. itinerans* genome provides deep insights into the lineage-specific diversification and also valuable resources for future banana breeding.

Crop wild relatives (CWRs) are composed of progenitors and more or less distantly related species of crops^1^. They were gene pools for many desirable traits including yields, nutrition quality, resistances to pests and diseases, and abiotic tolerances to drought, salt, cold etc. Due to substantial loss of genetic variations during domestication, several domestication syndromes such as disease susceptibility were common to many crop species. To help cope with these challenges, plant breeders have resorted to CWRs, and made significant advances in the past 30 years^2^. However, due to climate change and human-mediated disturbances, many of the CWRs were threatened and poorly conserved, so it’s imperative to take immediate action to collect, conserve and evaluate them^3^. In recent years, the coined term ‘super-domestication’ referred to the process of obtaining target traits by manipulation on crops or CWRs via genetic and genomic approaches^4^. Thus, genome sequencing of CWRs will facilitate the discovery of useful alleles or key genomic elements that can contribute to the improvement of targeted traits^5^. Banana is one of the most favoured fruits worldwide, and also an important staple food for people in some African and Latin American countries. Bananas are diploid or triploid hybrids between *Musa acuminata* (A genome) and *M. balbisiana* (B genome) or hybrids between subspecies of *M. acuminata*. Originally, domesticated in Southeast Asia about 7000 years ago^6^, the seedless and parthenocarpic hybrids have been thereafter widely propagated by vegetative reproduction. Long-standing human selections on these spontaneous triploids or diploids have contributed to the diversity of cultivated bananas^7^. Today, one triploid subgroup (AAA genome group), namely Cavendish that originated from a single clone, contributes to almost half of the world’s banana production. However, an emerging strain of *Fusarium* wilt disease, Tropical Race 4′ (*Fusarium oxysporum* f. sp. *cubense* race 4, Foc-TR4) has caused devastating damages to this cultivar. There are about 60 species in the *Musa* genus, and some of them have been utilized in banana resistance improvement, for instance, a wild non-edible accession of *M. acuminata* ssp. *burmannica* was once extensively used in banana breeding for its resistances to black Sigatoka and some races of *Fusarium* wilt^2^. Therefore, wild relatives potentially harbour beneficial alleles for cultivated crop, and are very important resources for future banana breeding strategies.

In 2012, the genome of *M. acuminata* ssp *malaccensis* (A genome), one progenitor of the cultivated banana, was sequenced and the genome evolution peculiarities of the Musaceae in the monocots were thus revealed^8^. Recently, the draft genome of the other progenitor of banana, *M. balbisiana* (B genome) has been released^9^. With the ever-decreasing costs of genome sequencing, it is becoming routine to sequence more genomes even within a genus, which can elucidate lineage-specific diversification mechanisms and genome diversities, as the case of rice^10^. Being one of the most valued crop species worldwide, the additional genome resources of banana are required to reveal hidden genetic diversity for future crop improvements. In this study, we contributed the draft genome sequence for *M. itinerans,* a close relative to both banana progenitors with wide distribution across subtropical China^11^. *Musa itinerans var itinerans*, known also as Yunnan banana, is native to south-east Asia and can be found in moist ravines to mountainous areas up to 2200 m^12^. In China, it is usually found in secondary tropical rainforests. It grows fast and can produce long rhizome with sucker emerging more than 2 meters from the mother plant, explaining its name origin. Interestingly, it was shown as one of the most Foc-TR4 resistant and cold tolerant species in the *Musa* genus^13^, providing valuable resource for the disease resistance and hardiness improvement in banana breeding. *M. itinerans* also displays interesting agronomic characteristic such as rapid fruit maturation (personal observation, Häkkinen M). Moreover, the studies will facilitate the understanding of the lineage-specific genome evolution in the *Musa* genus.

## Results and Discussion

### Genome sequencing and annotation

One individual plant of *M. itinerans* collected from Baoting County on Hainan Island and transplanted in South China Botanical Garden was sequenced by whole-genome shotgun strategy on the Illumina Hiseq2000 platform (Supplementary Note 1, Supplementary Fig. 1). A total of 74.2 Gb clean reads (approximately 120.7 fold coverage) derived from five libraries with insertion size ranging from 170 bp to 10 kb were used (Supplementary Table 1, Supplementary Note 2). Hierarchical assembly implemented in SOAPdenovo2^14^ yielded a draft genome of 462.1 Mb, representing 75.2% of the estimated genome size (615.2 Mb) based on a 17-mer statistics (Supplementary Fig. 2, Supplementary Table 2, Supplementary Note 3, 4). In total, the assembly comprised 7194 scaffolds (above 2 kb) with N50 of 192 kb, and 103,534 contigs (above 100 bp) with N50 of 33.9 kb (Table 1, Supplementary Table 3). The post-assembly quality evaluations showed that the distribution of GC content was concentrated around 0.388, well in accord with that of the published genome *M. acuminata*^8^, and the average base coverage was about 92× (Supplementary Figs 3 and 4). The total heterozygous rate was estimated to be 0.25% based on the ratio of the number of single-nucleotide polymorphisms to the overall genome length, after excluding ‘N’ sites of assembly gaps (Supplementary Note 4). Using the package CEGMA^15^, the complete and partial coverage of core eukaryotic gene sets (CEGMA) for the *M. itinerans* genome assembly were 79.8% and 94.4% respectively, which were very close to the *M. acuminata* genome assembly (81.9% and 93.6% respectively) (Supplementary Table 4). Thus, we obtained a qualified draft genome assembly, which should be valuable genomic resources to support banana breeding.

Using both homology-search against the RepBase library^16^, and *de novo* predictions with RepeatModeler approaches, 38.95% (179.99 Mbp) of the *Musa itinerans* genome were predicted to contain repetitive elements, somewhat higher than that of *M. acuminata* (35.43%, 167.59 Mbp) (Supplementary Table 5), and the higher level of repetitive elements for *M. itinerans* might be caused by using heterozygous diploids instead of the double haploid used for *M. acuminata.* Of the most abundant retrotransposons, long-terminal repeats (LTRs) were predominant in *M. itinerans*, and the two most common subfamilies LTR/Copia (16.7%) and LTR/Gypsy (16.3%) were almost in equal proportions in *M. itinerans*. The two peaks around sequence divergence rate were 20% and 30%, indicating that LTRs families in *M. itinerans* underwent two rounds of expansion events (Supplementary Fig. 5). Using the intact 5′- and 3′- LTR sequences of the retrotransposons within the genome, the insertion time of these LTRs for both *Musa* species were estimated, and quite large proportions of them burst after the divergence between *M. itinerans* and *M. acuminata* (approximately 5.8 million years ago (Mya), estimated by this study), suggestive of high turnover rates of retrotransposable elements in the *Musa* genomes. One recent burst of LTRs occurring at about 0.2 Mya in *M. itinerans* but not in *M. acuminata*, showed rapid dynamic evolution for these repeat elements (Supplementary Fig. 6). For *M. itinerans*, only 3.15% of the genome was the class II elements, and the compositions resembled those of *M. acuminata*, in which CACTA and Mariner, the most common superfamilies in other plants were absent. Instead, two super-families CMC (0.97%) and hAT (0.90%) were the most common types (Supplementary Table 5). Using the protein sequences of six species (*M. acuminata*, *Phoenix dactylifera*, *Oryza sativa*, *Sorghum bicolor*, *Zea mays*, and *Arabidopsis thaliana*) as queries against the repeat-masked assemblies, 32,456 protein-coding genes were predicted with an average coding sequence length of 1065 bp and 5.2 exons per gene (Supplementary Table 6). For these predicted genes, 86.9% of them were annotated with the SWISS-PROT, KEGG, GO, InterPro and TrEMBL databases (Supplementary Table 7). In addition to protein-coding genes, other non-coding RNA including 345 microRNA, 977 tRNA, 278 rRNA, and 299 snRNA were also identified (Supplementary Table 8).

### Genome evolution

#### Gene family expansions and contractions

**Figure 1:**
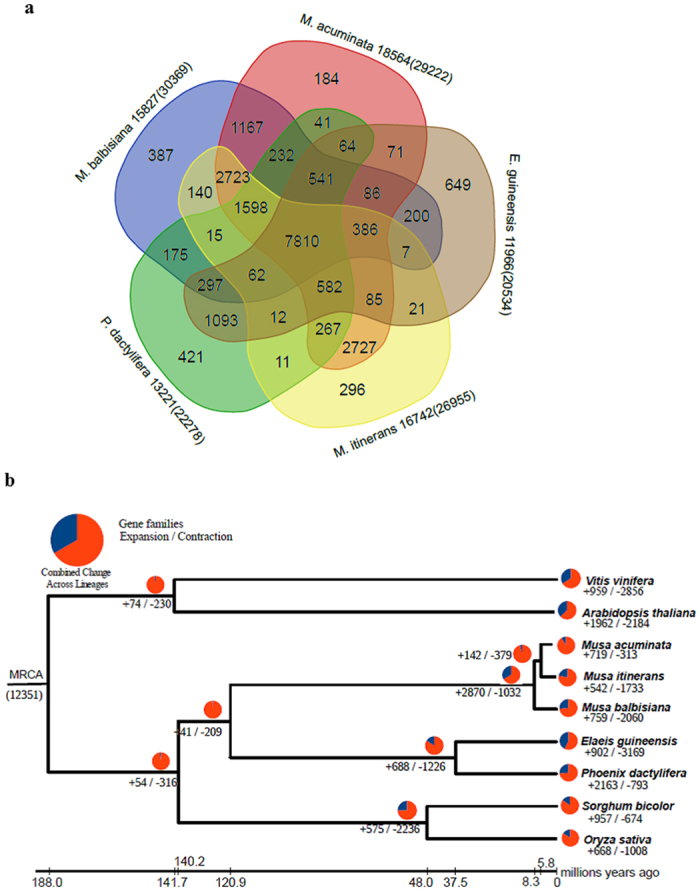
Gene family relationships between *Musa itinerans* and other sequenced genomes. (**a**) The Venn diagram represents shared/unique orthologous gene families between *Musa itinerans* and other three species (*Musa acuminata*, *Musa balbisiana*, *Phoenix dactylifera*); (**b**) Gene family expansions or contractions in three *Musa* genomes. Numbers to the right and left of the slash were expanded (+) and contracted (−) gene families, respectively. Red and blue pie-charts indicate the proportions of changed gene families and background gene family sets, respectively. Maximum likelihood tree was constructed using four-fold degenerate transversion sites of 1201 single-copy gene families,and the values at the node indicated the estimates of divergence time (MYA) with a 95% credibility interval.

We conducted protein-based clustering with OrthoMCL among *M. itinerans* and other eight species, namely, *M. acuminata*, *M. balbisiana*, *Phoenix dactylifera*, *Elaeis guineensis*, *Oryza sativa*, *Sorghum bicolor, Vitis vinifera,* and *Arabidopsis thaliana.* Of the 28,524 predicted protein sequences (>100 amino-acids in length) in *M. itinerans*, 26,955 (94.5%) were assigned into 16,742 gene families with 1201 single-copy orthologs, and 177 gene families were found to be unique to *M. itinerans* (Supplementary Table 9, Supplementary Fig. 7). A 5-way comparison of the three *Musa* species and two closely related Arecaceae species *P. dactylifera,* and *E. guineensis* showed that 7810 gene families were shared among them, and 2723 uniquely shared gene families between the three *Musa* genomes (Fig. 1a). *M. itinerans* shared more gene families with *M. auminata* (2727 gene families) than with *M. balbisiana* (140 gene families), which were suggestive of higher similarity for the latter two species. The unique gene families of *M. itinerans* among the five species were significantly enriched for 40 GO terms and involved in some essential functional categories such as DNA polymerase activity (GO: 0034061, P = 1.56e-9, hyper-geometric test), endoribonuclease activity (GO: 0016891, P = 5.12e-9, hyper-geometric test), and ribonuclease activity (GO:0004540, P = 4.15e-7, hyper-geometric test) (Supplementary Table 10). Twelve genes were significantly mapped on the ribosome pathway (ko03010, P = 4.64e-5, hyper-geometric test) (Supplementary Table 11), and it has been suggested that ribosomes might play some roles in the intrinsic regulation of a subset of mRNAs translation with unique cis-regulatory elements^17^. The followed proteasome pathway (ko03050, P = 1.77e-5, hyper-geometric test) involved the degradation of abnormal or damaged proteins in the cell, and played crucial roles in response to diverse intracellular signals and changing environmental conditions such infection, heat and cold etc^18^. Other enriched pathways fell into diverse categories with development process, growth, response to biotic or abiotic stresses, such as plant hormone signal transduction (ko04075), inositol phosphate metabolism (ko00562), fatty acid biosynthesis (ko00061), plant-pathogen interaction (ko04626), and starch and sucrose metabolism (ko00500) etc. (Supplementary Table S11). Overall, these unique gene families of *M. itinerans* might also give some hints on how a subtropical marginally distributed species coped with the challenging environmental conditions.

Comparison of orthologous gene families among nine related species revealed that 2870 expanded and 1032 contracted gene families in the ancestor of the three *Musa* species (Fig. 1b). The expanded gene families were enriched in 29 pathways, of which the most striking expansions were associated with phagosome (ko04145, hyper-geometric test, P = 2.18e-20), and plant-pathogen interaction (ko04626, hyper-geometric test, P = 1.11e-19) (Supplementary Table 12). Phagosome biogenesis was a key process in tissue remodeling, clearing apoptotic cells, and restricting the spread of intracellular pathogens^19^, together with the plant-pathogen interaction pathway (ko04626), both of which provided some clues on how tropical emergent taxa coped with the intrinsic or exogenous biotic stresses. Besides these two important functional categories, the remaining 27 pathways involved in many other important component biogenesis processes, and might be associated with some phenotypic or physiological innovations in the ancestral *Musa* species (Supplementary Table 12). In contrast, the ancestral *Musa* species lost fewer gene families, and only 15 pathways were significantly over-represented (Supplementary Table 13). One noteworthy pathway, phenylalanine metabolism (ko00360, P = 3.44e-05, hyper-geometric test) was considered to be a key step in the lignin biosynthesis of vascular plants, and the contraction of this pathway might perturb the lignin biosynthesis and cell-wall architecture^20^, thus might result in the crucial transition from a woody to herbaceous disposition in the ancestors of *Musa* species. As one of the most marginally distributed *Musa* species in subtropical areas, 21 expanded and 24 contracted pathways were enriched for *M. itinerans* (Supplementary Tables 14 and15), some of which were in accordance with the release from biotic-stresses such as pathogens (ABC transporter^21^, ko02010, P = 1.25e-17, hyper-geometric test; plant-pathogen interaction, ko04626, P = 2.28e-9, hyper-geometric test), and intensification of abiotic stresses such as drought and cold (phagosome^19^, ko04145, P = 6.61e-24, hyper-geometric test; amino sugar and nucleotide sugar metabolism^22^, ko00520, P = 8.13e-13, hyper-geometric test).

#### Genome duplication and divergence

According to a calibrated divergence time of 187.9 Mya (95% confidence interval:124.0~248.4 Mya) between *Arabidopsis* and *sorghum*^23^, the divergence time between *M. itinerans and M. acuminata* was estimated to be 5.8 Mya (95% confidence interval: 3.4~9.0 Mya) based on four-fold degenerated sites (4DTv sites) of 1201 single-copy orthologs, and the ancestor of *M. itinerans and M. acuminata* diverged with *M. balbisiana* about 8.3 Mya (95% confidence interval: 4.8~13.1 Mya), and Musaceae and Arecaceae coalesced to 120.9 Mya (95% confidence interval 73.4~181.2 Mya) (Fig. 1b, Supplementary Fig. 8).

**Figure 2:**
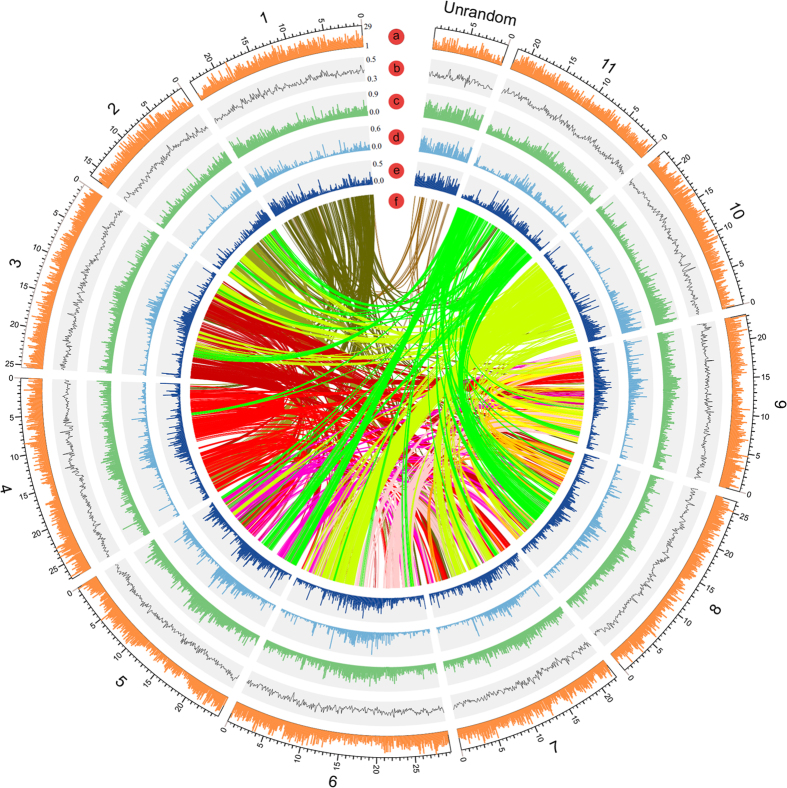
The global view of *Musa itinerans* genome. Pseudo-chromosomes of *M. itinerans* were constructed by anchoring and orienting the scaffolds of *M. itinerans* among the chromosomes of *Musa acuminata* based on the collinearity between them. Concentric circles were shown by window size of 100 kb. Tracks displayed were: (a) gene density, min = 1, max = 29; (b) GC content ratio, min = 0.3, max = 0.5; (c) all repeat content ratio, min = 0, max = 0.9; (d) LTR/Copia content ratio, min = 0,max = 0.6; (e) LTR/Gyspy content ratio, min = 0, max = 0.5; (f) intragenomic collinearity.

**Figure 3:**
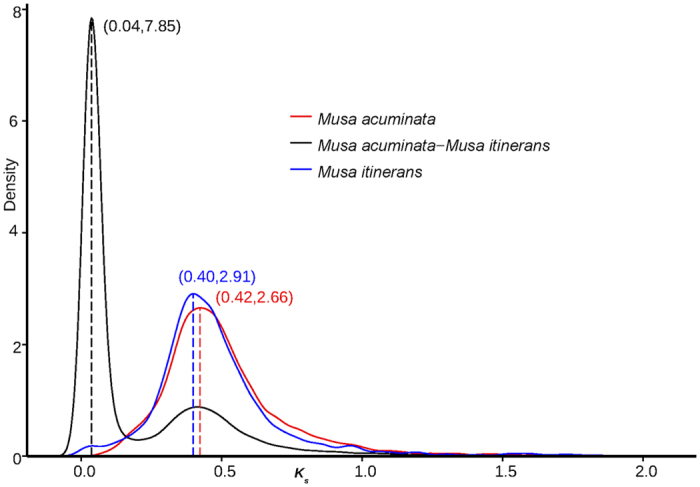
Whole genome duplication within and speciation events among *Musa itinerans* and *Musa acuminata*. The distributions of 4DTv (transversions at fourfold degenerate sites) distances between orthologs and the distributions of 4DTv between paralogs within genomes were used to infer the possible speciation events and whole-genome duplication events, respectively.

Using SynMap, a web-based tool on the plant comparative genome platform CoGe (https://genomevolution.org/CoGe/), syntenic regions between *M. itinerans* and *M. acuminata* were generated. Based on the collinear blocks between *M. itinerans* and *M. acuminata*, 26,670 genes, comprising of 82.5% of the *M. itinerans* gene sets distributed on 1043 scaffolds, were oriented and anchored to the 11 linkage groups (Supplementary Fig. 9, Supplementary Table 16). The global view of the genome *M. itinerans* including gene density, GC content distribution, repeat elements distribution were also plotted along the pseudo-chromosomes (Fig. 2), and it showed that these genes unevenly distributed among given window slides (P = 0, Chi-square test), and positively correlated with the GC content significantly (P = 2.2e-16, Spear’s rank correlation rho = 0.35), as predicted by GC content and gene density^24^. Using the MCScanX Program^25^, 510 syntenic blocks covered 36.25% of gene repertoire of *M. itinerans* were identified, with average number of 13, ranging from 6 to 164 (Fig. 2f); and 695 syntenic blocks composed of 36.10% of the final gene set in *M. acuminata* were found (Supplementary Fig. 10). Based on the distributions of synonymous substitutions rate (*Ks*) of these syntenic orthologous and paralogous gene pairs, the speciation and paleoploidization events in the *Musa* genus were inferred. When following an average *Ks* of 3.47 per 10^9^ years for nuclear genes in the Zingiberales^8^, *M. itinerans* and *M. acuminata* diverged approximately about 5.8 million years ago with peak *Ks* around 0.04 (Fig. 3). This divergence time estimation was consistent with that based on the phylogeny of 1201 single copy nuclear genes among nine plant species (Fig. 1b, Supplementary Fig. 8). For both *Musa* species, one peak around *K*_*s*_ 0.40~0.42 for intragenomic syntenic paralogs indicated an ancient genome duplication event occurred about 57.6~61.0 Mya, which was consistent with the polyploidization explosions in many plant species around Cretaceous-Tertiary boundary^26^. According to the ancestral syntenic blocks inferences in *Musa*^8^, this genome duplication should correspond to the two in separate successive duplication events (α/β) in *M. acuminata.*

### Evolution of NBS and MYB gene families

Following the method described in D’Hont *et al.*^8^, 62, 93, 117 NBS-encoding genes were identified in the genomes of *M. itinerans, M. balbisiana, M. acuminata,* respectively (Supplementary Table 17). The decreasing number of NBS genes was consistent with the latitudinal ascending of the three species. During the transition from humid tropical to cool subtropical habitats, some NBS related genes would be less numerous due to relaxed selection constraints on them. The absence of Toll/interleukin-1 receptor (TIR)-like (TIR-NBS types) in the three *Musa* species was consistent with previous reports in monocots^27^, and these NBS genes were distributed unevenly among the 11 chromosomes with skewed distributions on chromosome 3, 6, 9, respectively (Supplementary Fig. 11). A large proportion of the NBS-encoding genes were singletons, and only a few of them clustered into gene families (Supplementary Table 17, Supplementary Fig. 12). The predominant transposed duplication mode for the NBS-encoding genes might contribute to the widespread singletons (Supplementary Tables 18 and 19), which was prone to cause drastic gene structural divergence and function diversification^28^. Transposed duplication modes were found to be responsible for the proliferation of other important gene families including MADS-box, F-box, B3 transcription factors in Brassicales^29^. In addition, transposed duplications were observed to span wider time scales than other duplication modes in both *Musa* species (Supplementary Fig. 13). With duplication ages in terms of *Ks* ranged from 0.01 to 5.50 with average 1.59 for *M. itinerans*, and from 0.02 to 3.93 with average 0.83 for *M. acuminata*, vast majority of the NBS-encoding genes were duplicated well prior to the divergence of *M. itinerans* and *M. acuminata* (*Ks* = 0.04), hence, the ancient ages of these duplicated NBS-encoding genes were not in accordance with the classic arm-race model, which predicted a high turnover rate and predominant young NBS-encoding genes within a genome^30^,^31^. *K*_*a*_/*K*_*s*_ (nonsynonymous to synonymous substitution ratio) values of most duplicated pairs (segmental duplication, tandem duplication, proximate duplication, transposed duplication) within both genomes were less than 1, indicating that purifying selection played an essential role in the maintenance of intra-genomic diversity of these NBS*-*encoding genes in *Musa*, as the cases in chestnut^32^, sorghum^33^, *Solanum*^34^. In our comparative studies in *Musa*, the NBS-encoding gene repertoire seemed to be associated with their specific habitat shift.

Transcription factor (TF) genes for the two *Musa* species were identified by blast against known transcription factor gene families of related species, as well as homology prediction using all the known transcription factor proteins to blast against the *M. itinerans* genome. In total, 3176 and 2898 putative transcription factor genes, from 58 families, were identified for *M. itinerans* and *M. acuminata*, respectively (Supplementary Table 20). These numbers represent 9.8% and 7.9% of the 32,456 and 36,542 predicted protein-coding loci. Among the 58 transcription factor families identified, MYB, AP2, Dof, G2-like, SBP, HD-zip, WOX, RAV, LBD and SAP were over-represented in both *Musa* species relative to other taxa (Supplementary Fig. 14). Similarly to *M. acuminata*, the *M. itinerans* genome contains a large number of TFs that can be explained by the 3 rounds of specific WGD in the Zingiberales order^8^.

The MYB family was the largest transcription factor family in the two *Musa* species. With the number of repeats in the N-terminal conserved MYB DNA-binding domain, the MYB super-family can been categorized into four classes, 1R-, R2R3-, 3R-, and 4R-MYB, and the largest one R2R3 MYB was plant specific and key regulators in metabolism and responses to biotic and abiotic stress, also in flowering or fruit development^35^. About 292 and 304 *MYB* genes were identified in the genomes of *M. acuminata* and *M. itinerans* after manual inspection, respectively (Supplementary Table 21, Supplementary Fig. 15), and 92.1% and 93.4% of them were R2R3-type *MYB genes*, and much higher than in Arabidopsis (56.8%) and rice (70.1%)^36^. The extensive collinearity between *M. itinerans* and *M. acuminata* for these MYB gene pairs was observed (Supplementary Fig. 16a). The intragenomic duplicated MYB gene pairs also showed good synteny and relatively even distribution across chromosomes (Supplementary Fig. 16b,c). For *M. acuminata*, this gene family arose primarily by segmental duplications (about 186 gene pairs) with median Ks = 0.57 and followed by transposed duplications with median Ks = 0.89 (about 97 gene pairs), both of which were prior to the ancient α/β whole genome duplication events (Ks = 0.40~0.42) in Musaceae (Supplementary Table 22). The MYB TFs’ gene families are essential to plant morphogenesis and diverse physiological processes, and the gene balance hypothesis assumed that these regulating gene families were dosage sensitive and robust to gene loss after whole genome duplication events^37^.About 89.7% of these segmental duplicated gene pairs were under purifying selection (with *K*_*a*_/*K*_*s*_ < 1), followed by transposed duplications (about 97 gene pairs, 84.6% of them with *K*_*a*_/*K*_*s*_ < 1). Under this scenario, purifying selections were expected to wipe out deleterious mutations and stabilize the MYB TFs gene repertoire for their essential functions. Similar evolutionary patterns were observed in *M. itinerans* (Supplementary Table 23), indicating that the conservative evolutionary pattern of MYB transcription factor families in the Musaceae family.

### Demographic history of *M. itinerans*

**Figure 4:**
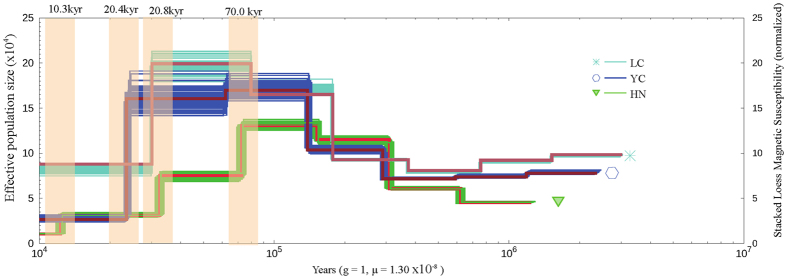
Effective population size changes of *Musa itinerans* over time using the Pairwise Sequentially Markovian Coalescent (PSMC) model. Using a generation time (g) of one year per generation, and a mutation rate of 1.30 × 10^−8^ substitutions per generation, the PSMC results were scaled to real time. Three different geographic populations were marked by distinctive color curves, and the most drastic population size change was shadowed. As shown, the effective population sizes of the two continental populations (YC, LC) were downsized in closely approximate epoch, and more recent than the island population (HN).

To infer the demographic history of *M. itinerans,* each individual from three populations along different latitudes were sampled and re-sequenced in high depth (HN: 71.0-fold coverage; LC: 48.4-fold coverage; YC: 54.8-fold coverage) (Supplementary Note1). Using the pairwise sequentially Markovian coalescent (PSMC) model^38^, the population size changes of the three populations were inferred respectively. The PSMC model estimated the coalescent time distributions between two alleles across all chromosomes based on the density of heterozygous sites across the diploid genome of a single individual, which in turn can be transformed into effective population size (*N*_*e*_) since the inverse relationship between the effective population size and coalescent rate. The population history analysis of each individual from one population depicted the nearly identical trajectory (Fig. 4). Using a generation time of one year and mutation rate 1.30 × 10^−8^ substitution rate per site per year^39^, the genomic variations of the three populations coalesced between 3Ma and 300 kya. Since approximately 150 Kya, the *N*_*e*_ of the marginal ancestral HN population (HN population, Hainan, China) was substantial less than either of the two central populations (LC, Lechang population, northern Guangdong province, China; YC, Yangchun population. southern Guangdong, China), and this spatial pattern of genetic variations was consistent with theoretical prediction^40^,^41^. The effective population size of the three populations increased since 300 kya and lasted until 70 kya for the HN population and about 20 kya for the two continental populations. During the Pleistocene cycles of glacial and interglacial episodes, frequent population bottlenecks and subsequent expansions were expected to reduce the effective population sizes, however, population migrations and concomitant admixtures during expansions among highly heterogeneous populations might lead to increased *N*_*e*_^42^ on the other hand, the heterogeneous topography in South China would result in the persistent population substructure, which was also possible for the elevated Ne during the glacial and interglacial cycles. It’s possible that the counter effect of admixtures between populations of *M. itinerans* overrode that of the population bottleneck until the progressive disappearance of population heterogeneities at the Last Glacial Maximum (LGM, c.a 18–20 Kya). However, due to genetic deficiency or less frequent admixtures, the isolated HN population showed more rapid decay of heterogeneity and earlier shrinkage of Ne than either of the continental populations (Fig. 4). The final formation of the Qiongzhou Strait was in the Middle Holocene when marine strata transgressed the original lowland, and the Hainan Island was completely isolated from Leizhou Peninsula^43^, so an additional Ne reduction of the HN Island population was observed at the time 10.3 Kya.

## Conclusion

Genome sequencing of the wild banana *Musa itinerans,* one of the most disease-resistant wild banana species, provides invaluable genetic resources for future banana genetic improvement. The *M. itinerans* diverged from *M. acuminata* only 5.8 Mya, and exhibited extensive collinearity between them, but the two species occupy distinct habitats, which provides an unpreceded chance to unravel the diversification within the *Musa* genus. Resorting to lineage-specific gene family expansions and contractions approaches, a batch of candidate genes categorized into diverse pathways or GO terms were inferred to be associated with some peculiar traits. However, more delicate experiments were required to clarify their roles in the physiological or phenotypic innovations, as one of the most disease resistant and cold hardy species in the *Musa* genus, the gene repertoire space of *M. itinerans* should be mined in the future. The non-synonymous to synonymous ratio *K*_*a*_/*K*_*s*_ of <1 for most NBS-encoding gene family revealed pervasive purifying selections, together with the ancient duplication ages, both in support of the trench-warfare model hypothesis and against the classic arms-race model. A similar evolution pattern was also observed in the MYB transcription factor family, in which the purifying selection wiped out deleterious mutations and stabilize the MYB TFs’ gene repertoire for their essential functions. Demographic history has a profound effect on the genetic diversity of extant species. The isolated HN population of *M. itinerans* was more prone to genetic loss during glacial and interglacial cycles than the counterpart mainland populations, in which admixture differentially buffered against genetic loss. Furthermore, the detailed scenarios of demographic history for this marginally distributed species will be revealed with more extensive sampling and population genome resequencing studies.

## Methods

### Sampling preparation and Sequencing

Natural populations of *M. itinerans* are distributed in subtropical China, and individual leaves of *M. itinerans* were harvested and immediately storied with sillica-gel in the field (the source of material see Supplementary Notes 1). The total genomic DNA was extracted with the CTAB (cetyl trimethylammonium bromide) method, and qualified with A260/280 ratio and agarose gel electrophoresis for further use. To obtain a high-quality reference genome, we sequenced one individual of *M. itinerans* from Hainan population using a whole-genome shotgun sequencing strategy. Paired and mate-pair Illumina genomic DNA libraries with varying insert size (180 bp, 500 bp, 2 kb, 5 kb, and 10 kb) were constructed following the manufacturer’s instructions. All the libraries were sequenced on the Illumina Hiseq2000 platform. Raw reads were subject to the removals of PCR duplicates, adaptor sequences, and contaminants of bacterial or viruses. The processed high-quality reads were assembled into contigs and scaffolds using SOAPdenovo2 package^14^ and gaps were filled using Gapcloser (version 1.12, http://soap.genomics.org.cn/soapdenovo.html). The reference genome size of *M. itinerans* was estimated based on the K-mer frequency distribution analysis (details for Supplementary Note 2). Two individuals each from the YC and LC populations were sampled and sequenced using a paired-end library size of 500 bp with >30-fold coverage.

### Genome annotation

For the draft genome of *M. itinerans*, interspersed repeats were identified using RepeatMasker v3.30 (http://www.repeatmasker.org) and homolog research for the Repbase database^16^. Repeat proteins were characterized using RepeatProteinMask v3.30 with default parameters, *de novo* interspersed repeats were annotated using RepeatModeler (http://www.repeatmasker.org/RepeatModeler.html). Tandem repeats in the genome of *M. itinerans* were screened using Tandem Repeat Finder v4.04^44^. Next, a homology searching approach was used to identify protein-coding genes. We obtained the protein sequences of *Musa acuminata* v1 from the Banana Genome Hub^45^ and *Phoenix dactylifera* DPV01, *Oryza sativa* IRGSP-1.0, *Sorghum bicolor* v3.1, *Zea mays*, and *Arabidopsis thaliana* TAIR10 from Phytozome (https://phytozome.jgi.doe.gov) or related databases (Supplementary Note 5) and then blasted against the repeat-masked genome assembly of *M. itinerans* using TBLASTN^46^ with percentage identity over 70% and coverage not less than 50%. Gene models were predicted based on these alignments using Genewise^47^. *De novo* gene predictions were performed using Augustus^48^ and GENSCAN^49^. Both sets of genes were integrated using GLEAN^50^. The annotations of the final gene sets were obtained by searching against the protein database KEGG^51^, SwissProt^52^, TrEMBL^53^ using an e-value cut-off of 1e-5, and motifs and domains for the gene set were determined using InterProScan^54^. Using CEGMA package, the completeness of the gene space for the draft genome assembly was evaluated with the highly conserved 248 core eukaryotic gene (CEGs).

For noncoding RNAs, tRNAs were identified using tRNAscan-SE^55^, snRNAs and miRNAs, and rRNAs were obtained by searching the genome assembly against the Rfam database using InFERNAL with default parameters (http://infernal.janelia.org/). All the raw short reads, genome assembly were deposited in NBCI’s Sequence Read Archive (SRA) database with accession number PRJNA312694, and the gene annotation used for the analyses were provided to the Banana Genome Hub (banana-genome-hub.southgreen.fr/organism/Musa_itinerans).

### Genome-wide duplications and estimation of the insertion times for LTR retrotransposons

Using the web-based tool SynMap on the plant comparative genome platform CoGe (https://genomevolution.org/CoGe/)^56^, the syntenic regions between *M. itinerans* and *M. acuminata* were generated, and scaffolds or contigs of *M. itinerans* were mapped or oriented on the 11 pseudo-chromosomes of *M. acuminata*. Reciprocal BLASTP between protein sequences of two *Musa* species with E-value cut-off of 1e-5, were implemented to identify orthologs between species, and self BLASTP for both species were used to obtain paralogs within species. After removing the self-matches of the BLASTP results, the syntenic blocks (≥5 genes per block) were determined with the package MCScanX^25^. The remaining aligned results were used to generate dot plots. Paralogous gene pairs in each block within species were used to infer genome duplication events. Meanwhile, orthologous gene pairs in each block between species provided some insights into the speciation events. The four-fold transversion (4DTv) ratio for each gene pair in the block was calculated on concatenated nucleotide alignments with HKY substitution models, and the distributions of these 4DTv values were used to estimate the whole genome duplication events and speciation events.

The 5′- and 3′-LTR sequences of the full-length LTR retrotransposons were aligned, and the *K* value (the average number of substitutions per aligned site) were calculated in the package MEGA^57^. The insertion times were estimated using this formula: T = K/(2 × r), where r represents the average substitution rate and is estimated to be 1.30 × 10^−8^ substitutions per site per year in rice^39^.

### Phylogenetic analysis

The protein sequences of eight plant species including *Arabidopsis thaliana*, *Vitis vinifera*, *Oryza sativa*, *Sorghum bicolor*, *Phoenix dactylifera*, *Elaeis guineensis*, *M. acuminata,* and *M. balbisiana* were downloaded (sources details in Supplementary Note 5). After filtering out protein sequences with amino acid length of less than 50, the longest protein sequence was chosen among spliced variants. All the eight protein datasets together with those of *M. itinerans* were clustered into paralogous and orthologous groups using the program OrthoMCL v1.4 with the inflation parameter of 1.5^58^. Protein sequences of each single-copy gene obtained from the nine species were aligned using MUSCLE v3.8.31^59^, and corresponding nucleotide sequences alignment of different genes were concatenated into one supergene. Using 4DTv sites of the supergene with the nucleotide substitution model HKY85 + gamma, a maximum likelihood phylogenetic tree of the nine species was constructed using the package PhyML with NNI (Nearest Neighbor Interchange) tree improvement^60^. Bootstrap supports of the internal branches were evaluated by 1000 full-heuristic searches. Divergence time estimations between species were determined using MCMCtree in PAML 4.7^61^, and the “correlated molecular clock” and “REV” substitution model were used in the approximate likelihood calculation. The MCMC process was run for 2,000,000 steps and sampled every 20,000 steps. A calibrated divergence time of 124~248.4 Ma between *Arabidopsis* and *Sorghum* was used^23^. Gene family expansions and contractions of extant species and ancient lineages were estimated using CAFE v2.1^62^. Using a random birth and death model, the gene birth (λ) and death (μ = −λ) rates were estimated across the species tree composed of *M. itinerans* and eight other species, using the maximum likelihood method, and gene families with accelerated rate of expansion and contraction were determined with a threshold conditional P-value (P < 0.05).

### Gene family evolution

Using HMMER V3 (http://hmmer.janelia.org/software), protein sequences of *M. acuminata, M. itinerans,* and *M. balbisiana* were aligned against the raw hidden Markov model (HMM) corresponding to the Pfam NBS (NB-ARC) family (PF00931, downloaded from website http://pfam.sanger.ac.uk/). Following the method described in D’ Hont *et al.*^8^, the hit NBS-domain sequences were aligned and used to construct a new NBS HMM profile using the module ‘hmmbuild’ . Using this *Musa* specific model, we identified NBS- candidate proteins in the three *Musa* species respectively. TIR and LRR domains of these NBS-encoding amino acid sequences were identified with Pfam_scan. Coiled-Coil (CC) motif was identified using MARCOIL^63^ with a threshold probability of 90 and double-checked using paircoil^64^. Duplication modes of these NBS-encoding genes were identified in MCScanX^25^.

Transcription factors’ gene families (TFs) of the *M. itinerans* were identified by two complementary approaches. First, protein sequences of *M. itinerans* were BLASTP against the TFs of five other species including *Musa acuminata*, *Arabidopsis thaliana*, *Vitis vinifera*, *Oryza sativa*, *Phoenix dactylifera* (http://planttfdb.cbi.pku.edu.cn/). Second, *de novo* predicted TFs were obtained using all the TFs of the other five species to TBLASTN against the genome of *M. itinerans* and corresponding gene models were predicted using Genewise^47^. Using GLEAN^50^, non-overlapping TFs of *M. itinerans* were obtained. The duplication history of the MYB gene family was inferred using the programme MCScanX^25^.

### Demographic history inference

One individual from each of the three populations was sequenced in high depth (Supplementary Note 1), and qualified reads from the short insert size library (500 bp or 180 bp) were realigned to the assembly with BWA with default parameters^65^. Consensus sequences were called using samtools^66^, and converted into the Fastq format using bcftools and vcfutils in the package pairwise sequentially Markovian coalescent model^38^ (PMSC). The consensus sequences were split into non-overlapping 100 bp bins marked as homozygous or heterozygous and these split sequences were used to reconstruct the demographic history with the PSMC model with the following parameters: “N30 -t15 -r3 -b -p “4 + 5 * 3 + 4”. The variance of the simulation results was assessed with 100 bootstrap replicates. Finally, the PSMC profiles were scaled using generation time (*g*) of one year, and neutral mutation rate *μ* of 1.30 × 10^−8^ substitution per site per year.

## Additional Information

**Accession codes:** The whole Genome assembly sequences and raw reads have been deposited in GenBank DDBJ/ENA/GenBank under the BioProject ID PRJNA312694, with accession numbers SRR3180710, SRR3180652, SRR3180729, SRR3180738, and SRR3180744 for raw reads, and LVTN00000000 for genome assembly, and the version described in this paper is version LVTN01000000. The genome assembly and annotation files for *Musa itinerans* can also be accessed by The Banana Genome Hub (banana-genome-hub.southgreen.fr/organism/Musa_itinerans).

**How to cite this article**: Wu, W. *et al.* Whole genome sequencing of a banana wild relative *Musa itinerans* provides insights into lineage-specific diversification of the *Musa* genus. *Sci. Rep.* **6**, 31586; doi: 10.1038/srep31586 (2016).

## Supplementary Material

Supplementary Information

## Figures and Tables

**Table 1:** Statistics of the assembly of the *Musa itinerans* genome.

Statistics of genome assembly	Value
Assembled genome size (Mb)	462.1
Effective genome size (Mb)^a^	422.1
Sequencing depth (×)	120
Filtered data (Gb)	74.2
N ratio in assembled genome (%)	8.6
Number of scaffolds (scaffold’s length >=2 kb)	7194
Contig N50 (bp)^b^	33,903
Scaffold N50 (bp)^b^	192,092
GC content (%)^c^	38.8
Repeat rate (%)	38.9
Predicted protein-coding genes	32,456
Sequence anchored on chromosome(%)	57.0
Genes anchored on chromosome(%)	80.4

^a^Effective genome size, without calculating Ns.

^b^N50 values of the genome assembly were calculated using the fragments larger than 100 bp. ^c^GC, guanine-cytosine.

^c^GC, guanine-cytosine.
