# Supplementary material for: Whole genome sequencing of a banana wild relative Musa itinerans provides insights into lineage-specific diversification of the Musa genus
Source: Sci Rep. 2016 Aug 17;6:31586. doi: 10.1038/srep31586 (PMC4987669; doi:10.1038/srep31586)
Supplement: Supplementary Information [file srep31586-s1.pdf]

Supplementary Information

Whole genome sequencing of a banana wild relative *Musa itinerans* provides insights into lineage-specific diversification of the *Musa* genus

Wei Wu[1,*], Yu-Lan Yang[2,*], Wei-Ming He[2], Mathieu Rouard[3,4], Wei-Ming Li[5], Meng Xu[2], Nicolas Roux[3,4], Xue-Jun Ge[1,†]

1. Key Laboratory of Plant Resources Conservation and Sustainable Utilization, South China Botanical Garden, the Chinese Academy of Sciences, Guangzhou 510650, China;

2. BGI-Shenzhen, Shenzhen 518083, China

3. Bioversity International, Parc Scientifique Agropolis II, 34397 Montpellier Cedex 5, France

4. CGIAR Research Program on Roots, Tubers and Bananas (RTB)

5. Key Laboratory of Tropical Fruit Biology, Ministry of Agriculture, South Subtropical Crops Research Institute, Chinese Academy of Tropical Agricultural Sciences, Zhanjiang 524091, China

[†]Correspondence and requests for materials should be addressed to G. X (email: xjge@scbg.ac.cn)

[*]These authors contributed equally to this work.

Contents

Supplementary Information 1

Supplementary Notes 5

Supplementary Note 1. Plant materials 5

Supplementary Note 2. Reads quality control 5

Supplementary Note 3. Genome assembly 6

Supplementary Note 4 Estimation of the genome size and heterozygosity 6

Supplementary Note 5. The source origin of the used genomic data in this study 7

Supplementary Figures 8

Supplementary Figure 1. Morphological characters of *Musa itinerans* and sampling locations 8

Supplementary Figure 2. 17-mer-based analysis to estimate the genome size in *Musa itinerans* 9

Supplementary Figure 3. Sequencing depth distribution of the *Musa itinerans* genome.... 10

Supplementary Figure 4. GC content in the genome of *Musa itinerans*. 11

Supplementary Figure 5. Divergence rate distribution of classified transposable element (TE) families in *Musa itinerans* genome. 12

Supplementary Figure 6. The insertion time distributions of long terminal repeat (LTR) retrotransposons in the genomes *Musa itinerans* and *Musa acuminata* 13

Supplementary Figure 7. Gene family clustering of nine sequenced plant genomes. 14

Supplementary Figure 8. Phylogenetic relationships of *Musa itinerans* and other eight species based on four-fold degenerated transversion sites (4DTvs) using MrBayesian methods 15

Supplementary Figure 9. Syntenic dotplots between *Musa itinerans* -*Musa acuminata* comparison. 16

Supplementary Figure10. Intragenomic syntenic blocks of *Musa acuminata*. Chromosomes are represented as colored blocks. 18

Supplementary Figure 11. The distribution of NBS-encoding genes for *Musa acuminata*, *Musa itinerans*, *Musa balbisiana* along chromosomes. 19

Supplementary Figure 12. Maximum likelihood phylogenetic analysis of the NBS-encoding super gene family of *Musa* species. 20

Supplementary Figure 13. Age and selection intensities of NBS-LRR gene families with different duplicated modes hosted in two *Musa* species 21

Supplementary Figure 14. Hierarchical clustering of 58 transcriptions factors (TFs) among *Musa itinerans* and other five species. 22

Supplementary Figure 15. DNA-binding domains of R2R3-MYB transcription factor proteins in *Musa acuminata*, and *Musa itinerans* 23

Supplementary Figure 16. Collinearity mapping the orthologs MYB gene pairs between *Musa itinerans* and *M. acuminata*. 24

Supplementary Tables 25

Supplementary Table 1 Summaries of reads used for *Musa itinerans* genome assembly... 25

Supplementary Table 2 Genome size estimation of *Musa itinerans* with the 17-mer analysis. 26

Supplementary Table 3 Statistics of the assembly of the *Musa itinerans* genome............ 27

Supplementary Table 4. The completeness of *Musa acuminata* and *Musa itinerans* genome assemblies evaluated with coverage of the conserved 248 core eukaryote gene (CEGs) with CEGMA program. 28

Supplementary Table 5 Comparison of repeat contents in the assembled genomes of *Musa itinerans* and *Musa. acuminata* 29

Supplementary Table 6 Homology-based gene predication for *Musa itinerans* genome ... 30

Supplementary Table 7 Gene functional annotations based on different protein database for *Musa itinerans* genome. 31

Supplementary Table 8 Non-coding RNA genes in the *Musa itinerans* genome.............. 32

Supplementary Table 9. Gene family clustering among *Musa itinerans* and other eight related species 33

Supplementary Table 10. Gene ontology (GO) enrichment analysis of Musa itinerans unique gene families among five species[a] and shown for significantly[b] represented GO terms.. 34

Supplementary Table 11. KEGG pathway enrichment analysis of *Musa itinerans* unique gene families among five species[a] and shown for significantly[b] represented pathways..... 36

Supplementary Table 12. KEGG pathway enrichment analysis of expanded gene families for ancestral *Musa* species among nine species[a] and shown for significantly[b] represented pathways.. 37

Supplementary Table 13. KEGG pathway enrichment analysis of contracted ancestral *Musa* gene families among nine species[a] and shown for significantly[b] represented pathways... 39

Supplementary Table 14. KEGG pathway enrichment analysis of *Musa itinerans* 40
expanded gene families among nine species[a] and shown for significantly[b] represented.... 40
pathways.. 40
Supplementary Table 15. KEGG pathway enrichment analysis of *Musa itinerans* contracted gene families among nine species[a] and shown for significantly [b] represented pathways 41
Supplementary Table 16. Overview of *Musa itinerans* pseudo-chromosomes based on syntenic blocks between *M. itinerans* and *Musa acuminata* 42
Supplementary Table 17 Classifications[*] of NBS-containing Resistances in three *Musa* species *M. acuminata*, *M. balbisiana*, and *M. itinerans* 43
Supplementary Table 18 Genome-wide duplication modes and ages of NBS-encoding genes in *Musa itinerans*.. 44
Supplementary Table 19 Genome-wide duplication modes and ages NBS-encoding genes in *Musa acuminata* 45
Supplementary Table 20. Transcription factors across two *Musa* genomes and other related species 47
Supplementary Table 21 List of MYB transcripton factor genes for two *Musa* species..... 50
Supplementary Table 22. Genome-wide duplication modes and ages MYB transcription gene family in *Musa acuminata*. 63
Supplementary Table 23 Genome-wide duplication modes and ages of MYB transcription gene family in *Musa itinerans*... 77

Supplementary Notes

Supplementary Note 1. Plant materials

One individual used for draft genome assembly was a transplant in South China Botanical Garden, Guangzhou, Guangdong, which were sampled from Wuzhishan Mountain, Baoting, Hainan island (detailed GPS information were not available, and the population designated as HN); the other two individuals used for high depth sequencing were sampled from Jiufeng, Lechang, Guangdong province (25°20' 50.09N, 113°21' 03.34E, alt. 490m. and the population was designated as LC), and Baiyong Nature Conserve, Yangchun, Guangdong province (22°23'46.09N, 111°39'54.04E, alt. 90m, and the population was designated as YC). Fresh leaves were harvested and stored and dried in silica-gel for DNA extraction.

Supplementary Note 2. Reads quality control

The short-read assembly algorithm was sensitive to sequencing errors, we took stringent measures to check and filter the short reads generated by the Illumina Hiseq2000 platforms, including steps:

(1) Remove of adapter contamination. DNA adaptor dimers, empty loading or too small insert sizes (less than the read length) will lead to adapter contamination, and it was library specific. For pair-end reads, If Read1 contains a 3'-adapter, then Read2 should contain a 5'-adapter. The reads were filtered at a threshold when both Read1 and Read2 contained adapters 10 bp and with a mismatch rate ≤ 0.1.

(2) Deduplication of redundant reads. Base-calling duplicates were filtered with a threshold of Euclidean distance ≤ 3 and mismatch rate ≤ 0. Euclidean distance between two reads in the same tile were calculated following formula: $\sqrt{(X_1 - X_2)^2 + (Y_1 - Y_2)^2}$, where 'X' and 'Y' were coordinates recorded in the sequence identifier of the fastq format files.

(3) Low quality reads trimmed or discarded. Low quality sequences at the 3'-end were trimmed for some reads. Individual reads in all lanes were filtered with an

excess (> 10%) of 'N's and where > 40% of the bases had a PHRED quality score below Q7.

Supplementary Note 3. Genome assembly

A total of 74.2Gb (about 120.7-fold sequencing depth) qualified reads were used for genome assembly after the quality control and filtering. Using SOAPdenovo v 2.0, the reads from short insert size libraries (170bp and 500 bp) were split into 31-mers to construct a de Bruijn graph. Contigs were obtained for those reads exhibited unambiguous connections in the *de* Bruijn graphs. Considering contigs with length ≥ 100 bp, the N50 and N90 contig size was 33,903 bp and 3,668 bp, respectively.

Reads of mate-pair libraries (>=2kb) were aligned on the contigs to build scaffolds using the pair-end information. Contigs were linked into scaffolds sequentially from short to long insert sizes. For each contig pair, at least three paired-end reads anchored on it. For the same insert libraries, conflicting connections with more than one other contig at the 3' or 5'end were omitted from scaffold construction. Finally, the read pairs mapped to the local region around gaps were retrived and carried out a local assembly to fill the gaps. Using all short and long insert size libraries, we obtained scaffolds N50 of 192,092 bp and N90 of 19,491 bp length.

Supplementary Note 4 Estimation of the genome size and heterozygosity

Using the filtered reads from the short insert size libraries (170 bp and 500 bp), the genome size Genome size G was estimated as G = K_num/K_depth, where K_num was the total number of K-mers, and K depth is the overall depth estimated from the k-mer distribution. In our 17-mer analysis, the kmer number was 14,764,273,540. By plotting the occurrence of k-mers against the percentage of corresponding k-mers, the K-depth was 24 (Supplementary Figure S1), so the genome size of *M. itinerans* was about 615Mb.

To estimate the heterozygosity rate of *Musa itinerans*, all high quality reads were mapped to the genome assembly to call heterozygous SNPs using the software

SOAP2 (http://soap.genomics.org.cn/soapaligner.html) with the cutoff less than 5 mismatches. Next, SNP mining conducted upon the alignment results using the SOAPsnp (http://soap.genomics.org.cn/soapsnp.html). The effective sites were determined by criterion: quality score of consensus genotype over 20; all the mapped best and second best base covered by at least 4 unique reads; sequencing depth is more than 10-fold; and SNPs interval are at least 5 bp. Finally, the rate of the heterozygosity was estimated as the number of heterozygotic sites divided by the number of criterion effective sites.

Supplementary Note 5. The source origin of the used genomic data in this study

In this study, we used the genome data of other eight related species. The genome resources of these species as following: *Arabidopsis thaliana* TAIR v10 (ftp://ftp.arabidopsis.org), *Vitis vinifera* Genoscope.12X (http://www.genoscope.cns.fr/externe/GenomeBrowser/Vitis/) ,*Oryza sativa* IRGSP-1.0 (http://rapdb.dna.affrc.go.jp/download/irgsp1.html), *Sorghum bicolor* v3.1 (https://phytozome.jgi.doe.gov/pz/portal.html), *Phoenix dactylifera* DPV01 (ftp://ftp.ncbi.nih.gov/genomes/Phoenix_dactylifera/protein/), *Elaeis guineensis* EG5.Genes.V2 (http://genomsawit.mpob.gov.my/genomsawit/) , *M. acuminata* v1.0 and *M. balbisiana* (http://banana-genome.cirad.fr/).

Supplementary Figures

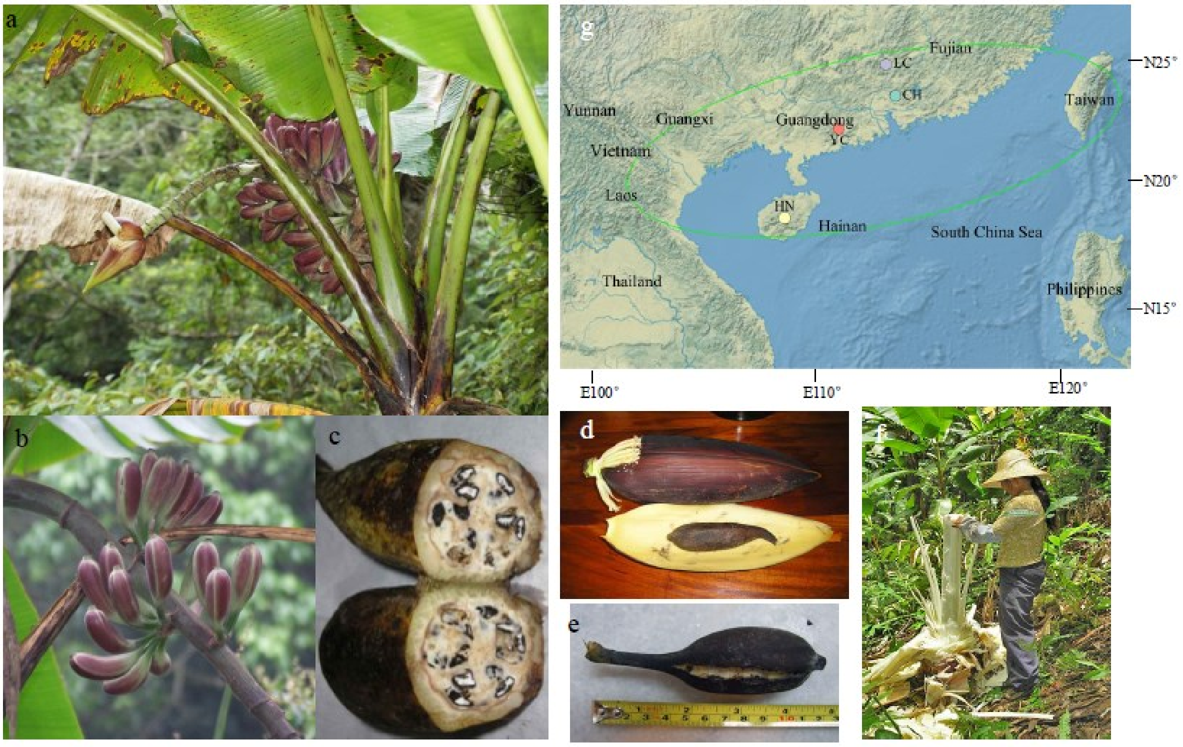

Supplementary Figure 1. Morphological characters of *Musa itinerans* and sampling locations. a. whole plant architectures; b. fruits; c. seeds; d. flowers and bracts; e. finger size; f. farmers harvesting the pseudo-stems for livestock food; g. sample locations of the *Musa itinerans* used in this study, LC, Lecang, Guangdong, CH, Conghua, Guangdong, YC, Guangdong, HN, Hainan, and the distributions of *Musa itinerans* outlined by green circle; The photographs of figures (a, b, c, d, e, f) were taken by Dr Ge, and the map (figure g) was generated with GenGIS 2.4.1(http://kiwi.cs.dal.ca/GenGIS/Main_Page) and manually modified.

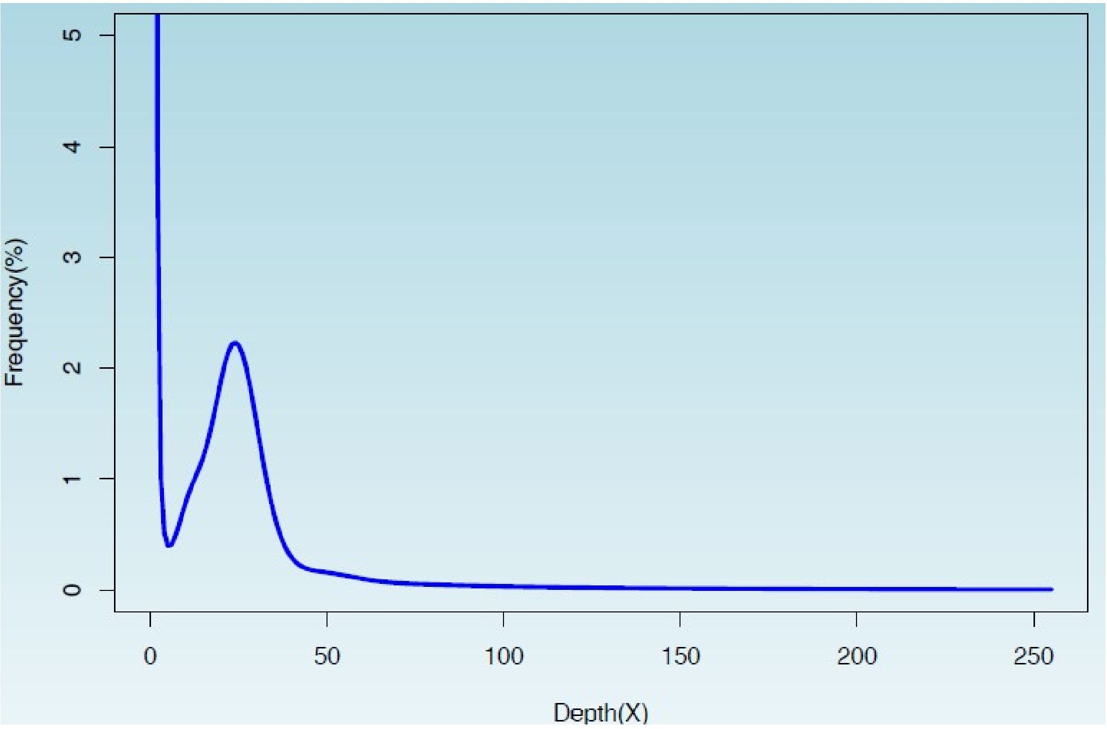

Supplementary Figure 2. 17-mer-based analysis to estimate the genome size in *Musa itinerans*. The X-axis is the depth of K-mers derived from the sequenced reads and Y-axis is the frequency of K-mer depth. One peak occurred at depth about 24-fold.

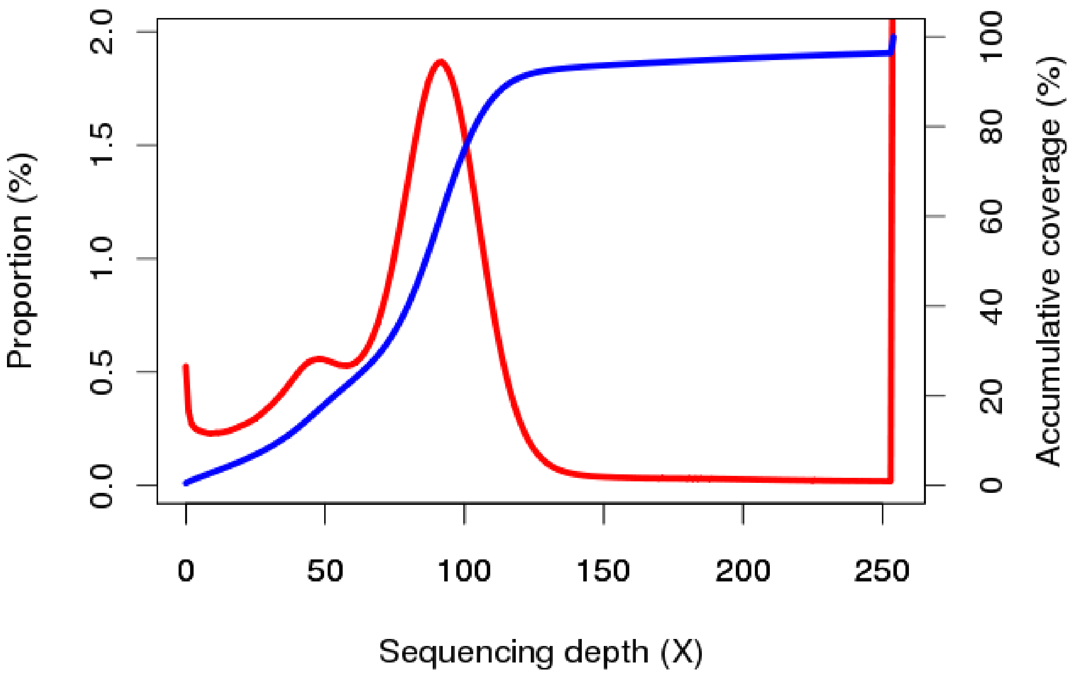

Supplementary Figure 3. Sequencing depth distribution of the *Musa itinerans* genome. Using SOAPaligner, all reads of the small insertion libraries were mapped back to the assembled genome, the proportions of the different base coverage to the genome were summarized using Soapcoverage v2.27; red curve represented the proportions of sequential base sequencing depths to the whole genomes, a peak at about 92 fold gave the average base sequencing depth, and minor peak at about 48 fold caused by heterozygosity; and blue curve depicted the distribution of accumulative base coverage.

a

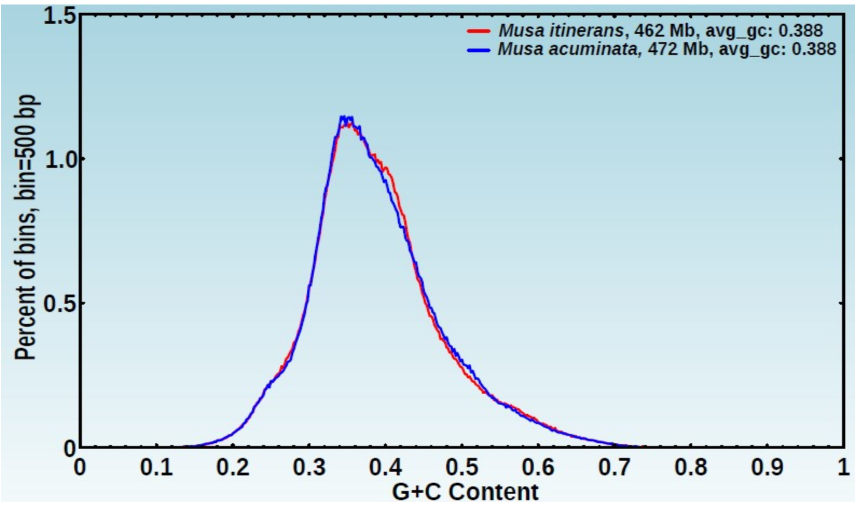

b

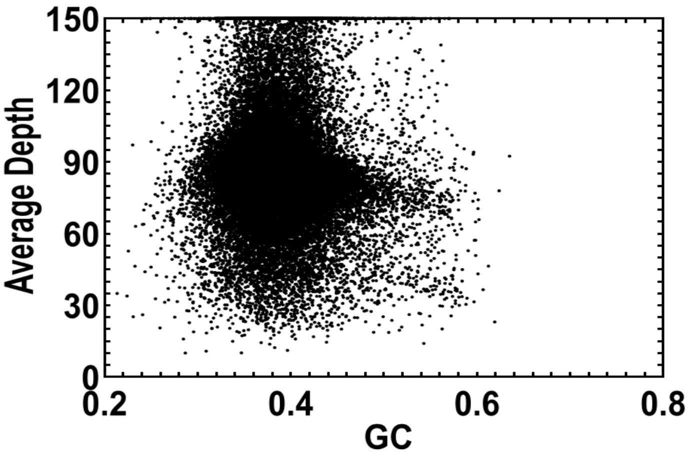

Supplementary Figure 4. GC content in the genome of *Musa itinerans*.

(a) GC content distributions of the genomes for two *Musa* species, blue and red curves represent the *M. acuminata* and *M. itinerans*, respectively. The x-axis is GC content and the y-axis is the proportion of the bin number with the specified GC. Each bin has a window size of 500bp (with 250bp overlap) sliding along the genome; (b) The sequencing depth and GC content for 10 kb non-overlapping sliding windows along the genome.

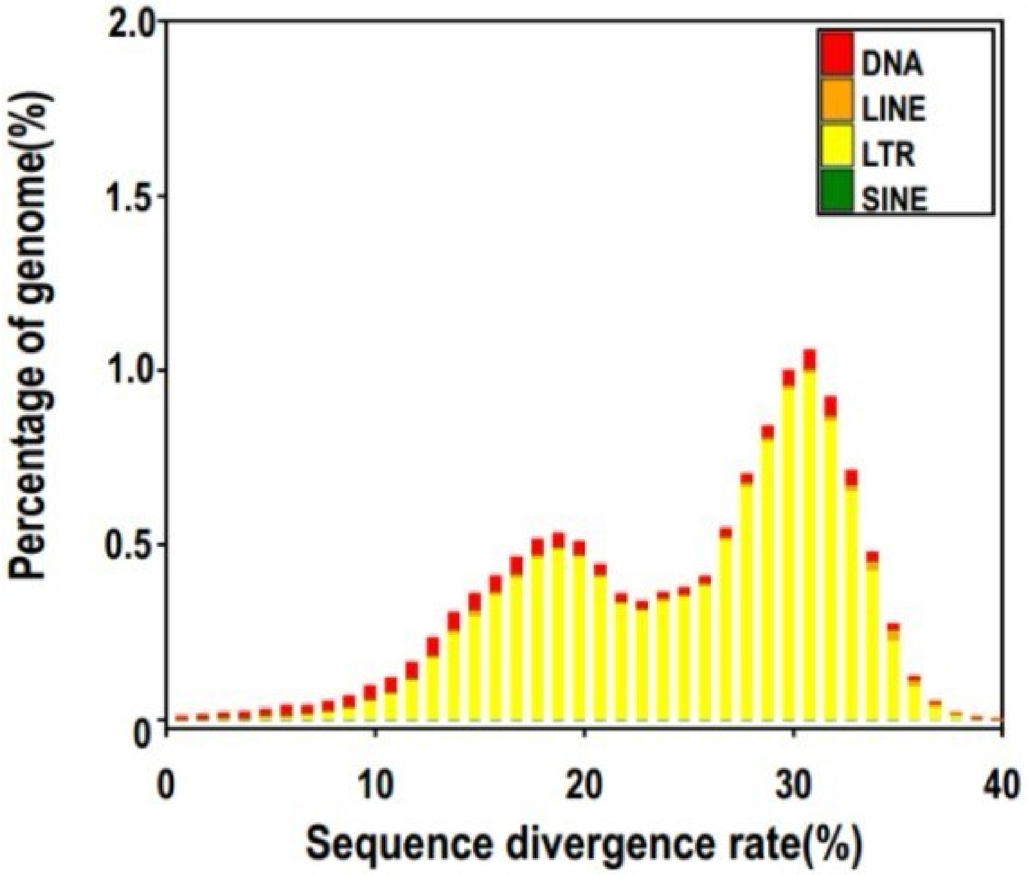

Supplementary Figure 5. Divergence rate distribution of classified transposable element (TE) families in *Musa itinerans* genome. The different classified TE families in the *M. itinerans* genome were aligned to the consensus sequences in the Repbase (v15.02) library. The sequence divergence rates of TEs were counted.

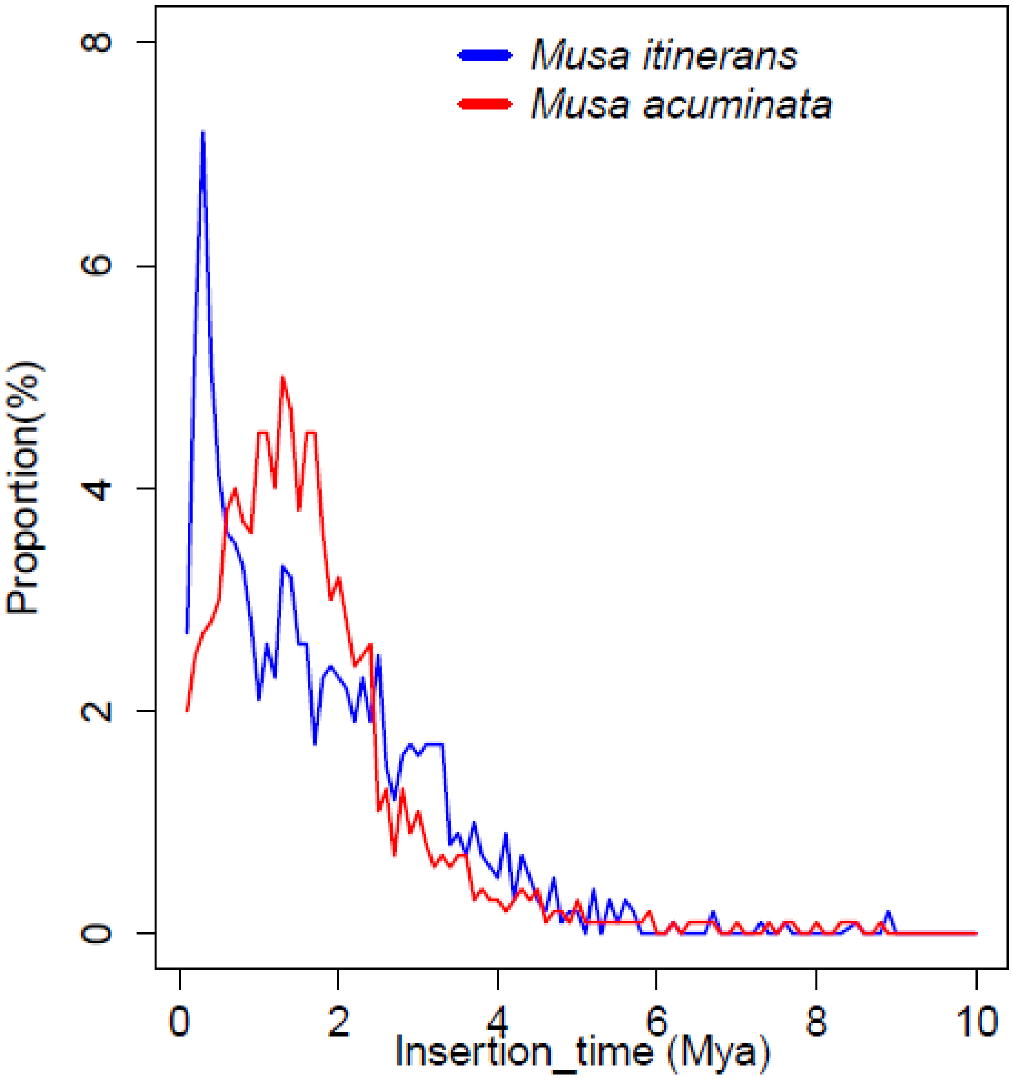

Supplementary Figure 6. The insertion time distributions of long terminal repeat (LTR) retrotransposons in the genomes *Musa itinerans* and *Musa acuminata*. Only sequences with intact 5' and 3' LTR were used to calculate the K-value (the average number of substitutions per aligned site) according to the formula: T = K/(2*r), implemented in MEGA. T denotes the insertion time, r is the average substitute rate for a given value 1.3×10^{-8} substitutions per synonymous site per year.

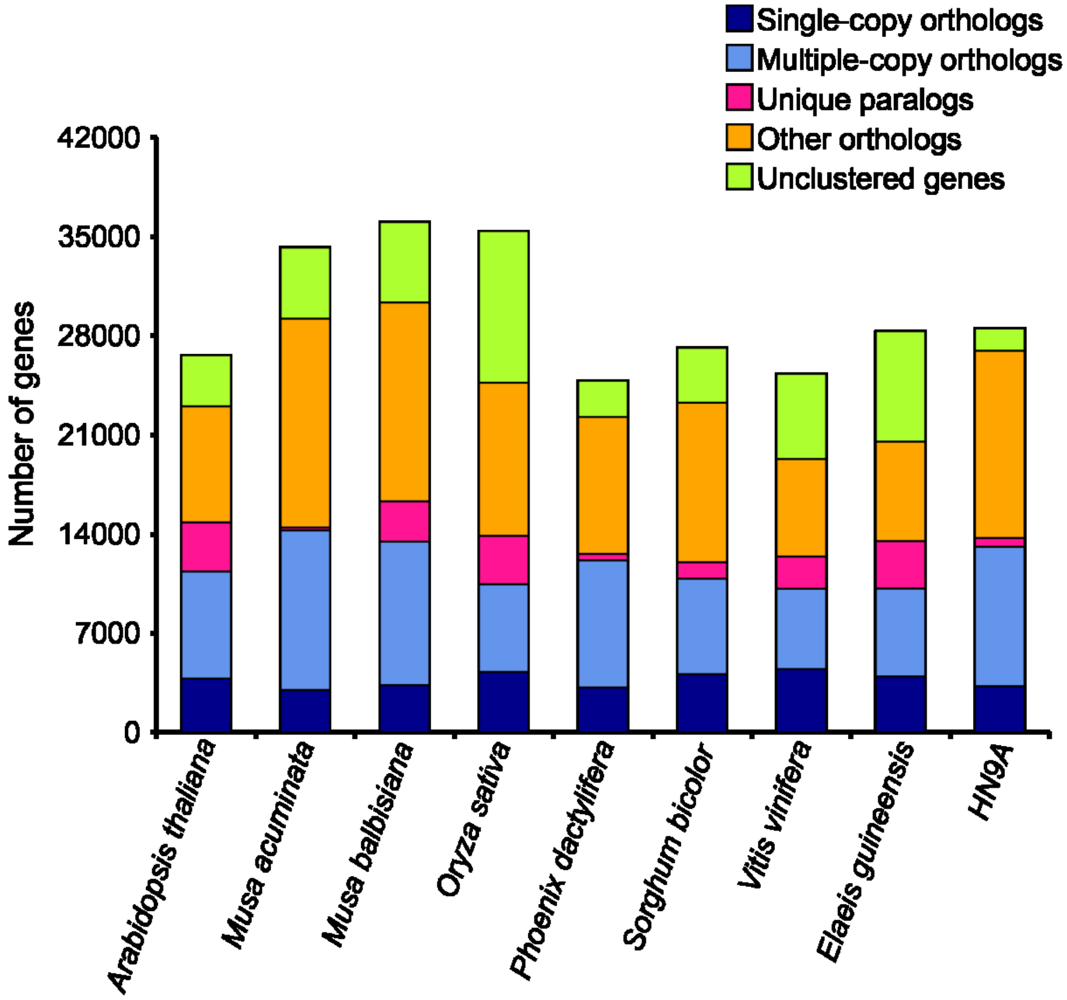

Supplementary Figure 7. Gene family clustering of nine sequenced plant genomes. The genes from nine sequenced plant genomes were aligned to each other using BLASTP with cut-off e value of 1e-5. Pairwise similarity of protein sequences was used to clustering all the genes.HN9A denotes the genome *Musa itinerans* in this study.

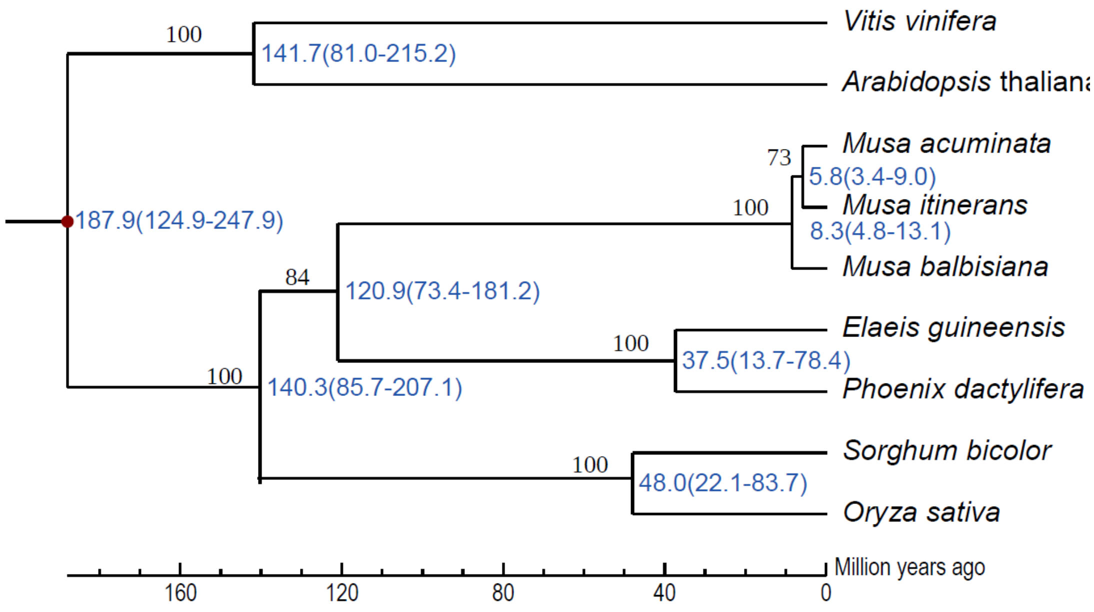

Supplementary Figure 8. Phylogenetic relationships of *Musa itinerans* and other eight species based on four-fold degenerated transversion sites (4DTvs) using MrBayesian methods. The numbers on the branch were bootstrap values, and the values at the node indicated the estimates of divergence time with a 95% credibility interval. A calibrated time of 141.7 (95% credibility interval: 81.0 ~215.2 MYA) for the ancestral node of *Vitis vinifera* and *Arabidopsis thaliana* was used

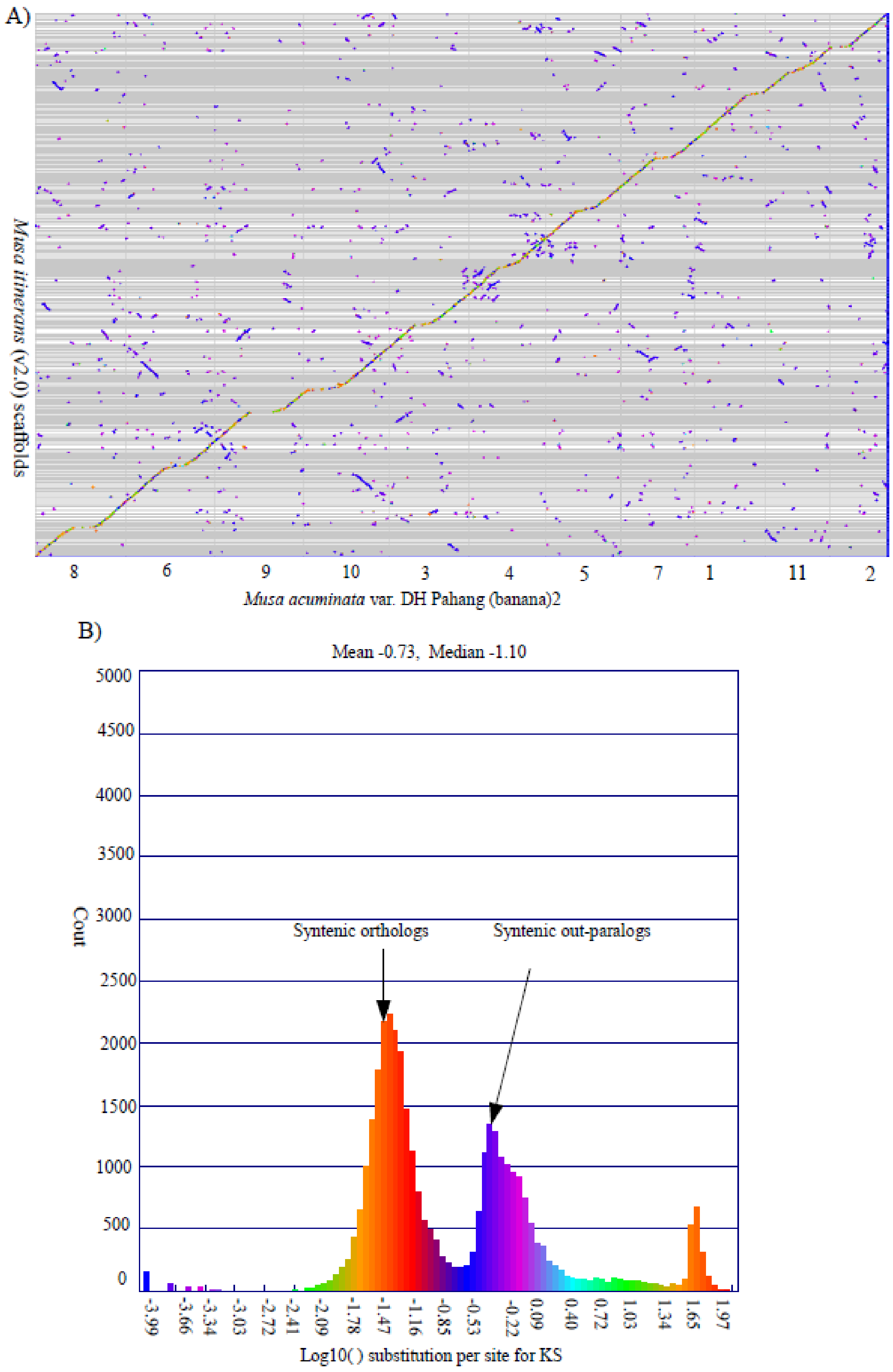

Supplementary Figure 9. Syntenic dotplots between *Musa itinerans*-*Musa acuminata* comparison. Syntenic gene pairs screened for a 3:3 syntenic depth.

(A) *Musa acuminata* is on the x-axis, and *M. itinerans* was on the y-axis. Horizontal and vertical gray lines separate chromosomes or scaffolds. Colored dots/lines were syntenic gene pairs through collinearity, and different colors corresponding to their Ks value shown in (B); (B) Synonymous rate value histogram of syntenic gene pairs. Values have been log10 transformed and colors on histogram match the syntenic dot colors shown in (A). Large peak on right of graph represents noise in the analysis due to erroneous gene models, pseudogenes, non-homologous gene-pairs etc.

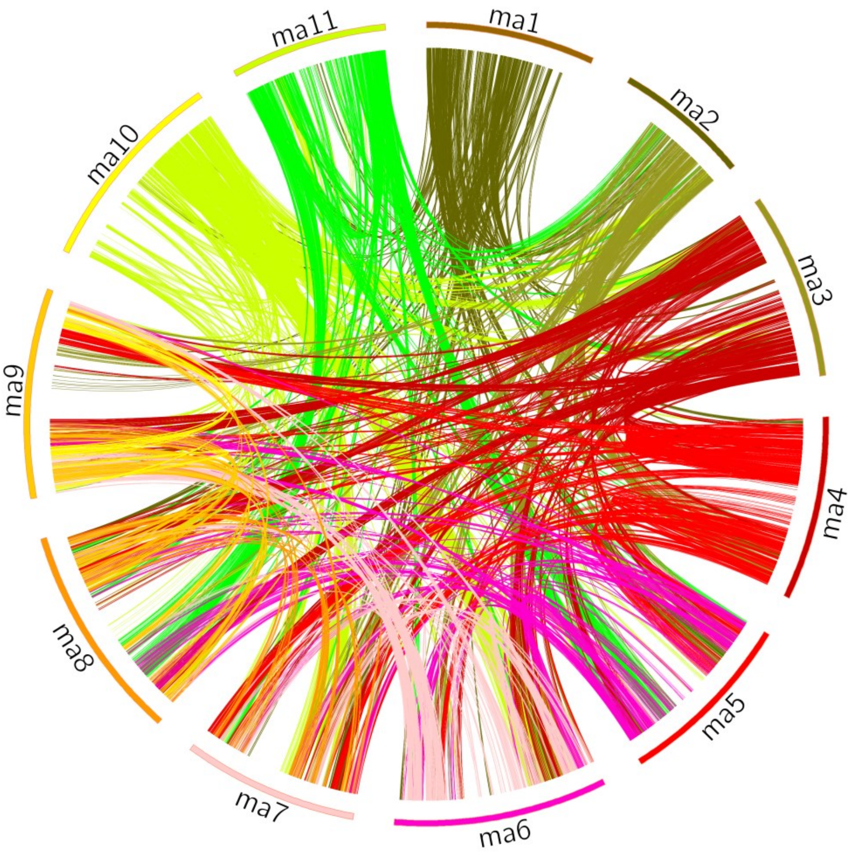

Supplementary Figure10. Intragenomic syntenic blocks of *Musa acuminata*. Chromosomes are represented as colored blocks. For clarity, links between chromosomes that share less than 5 gene pairs were omitted. The image was produced using Circos (http://circos.ca/)

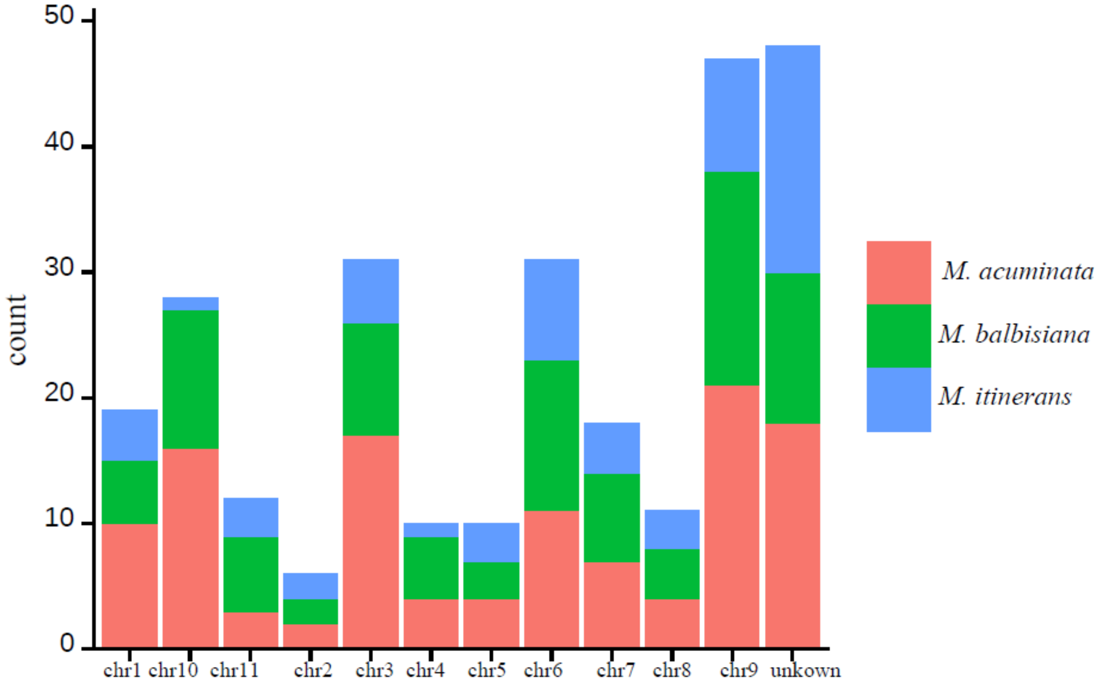

Supplementary Figure 11. The distribution of NBS-encoding genes for *Musa acuminata*, *Musa itinerans*, *Musa balbisiana* along chromosomes.

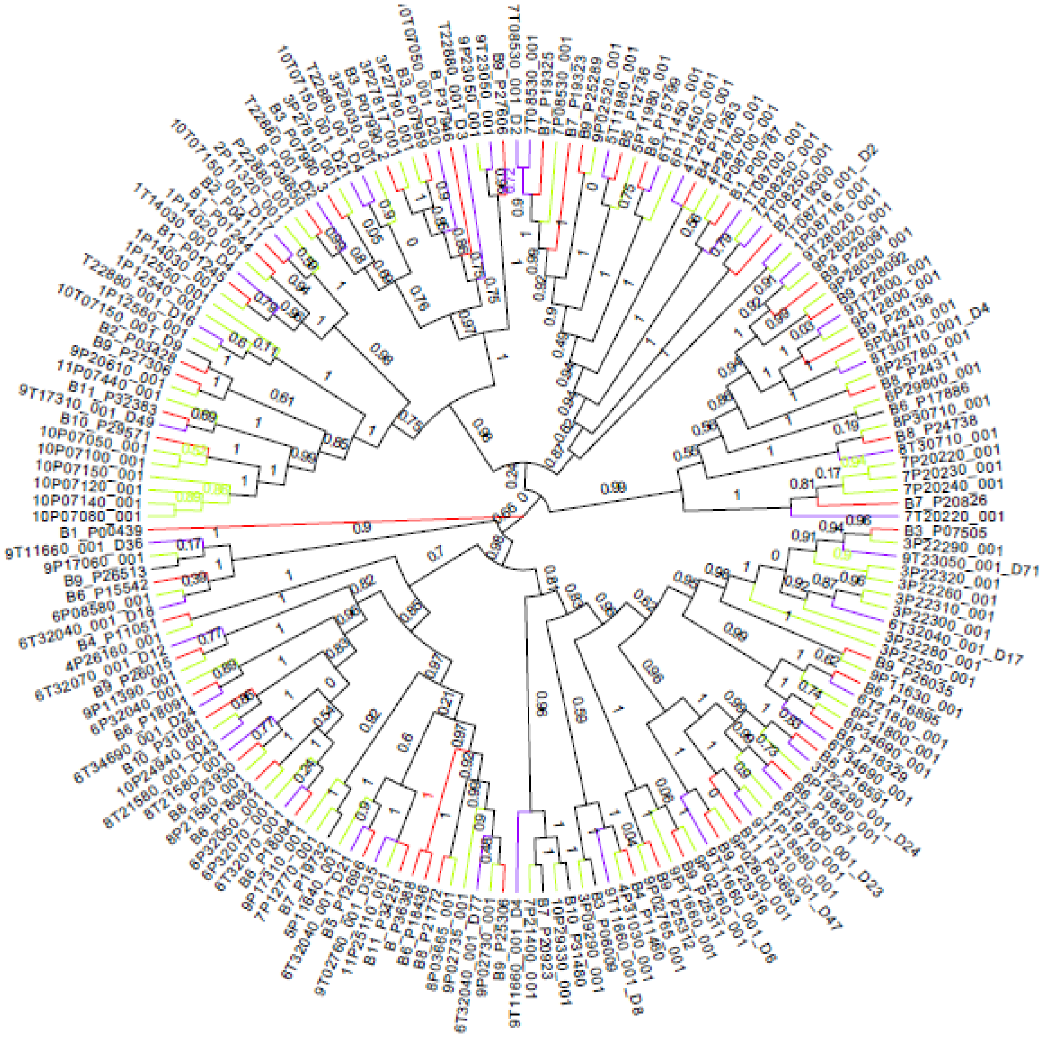

Supplementary Figure 12. Maximum likelihood phylogenetic analysis of the NBS-encoding super gene family of *Musa* species. The green tip branches denoted *M. acuminata,* blue tip branches represented *M. itinerans,* red tip branches represented *M. balbisiana*. Numbers on the branches are the bootstrap values. Intermingled branches between species and few clusters of gene families within species indicated the ancient polymorphisms or random lineage sorting.

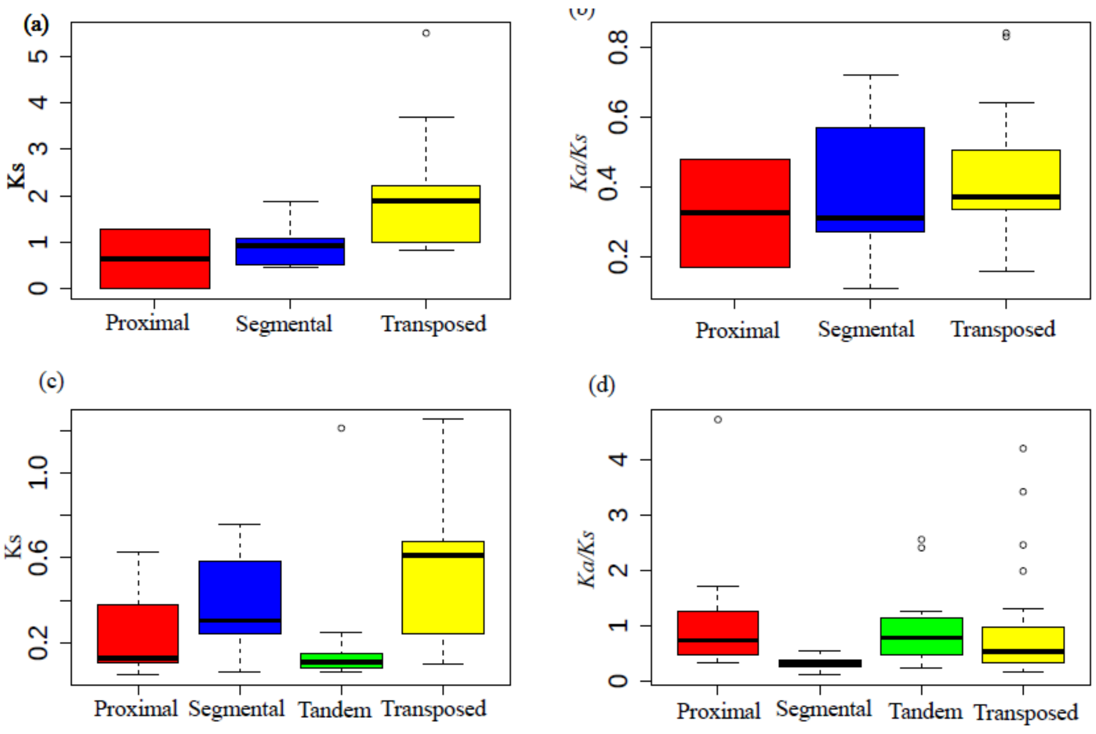

Supplementary Figure 13. Age and selection intensities of NBS-LRR gene families with different duplicated modes hosted in two *Musa* species. *M. itinerans* (Panel a and b) and *M. acuminata* (Panel c and d).

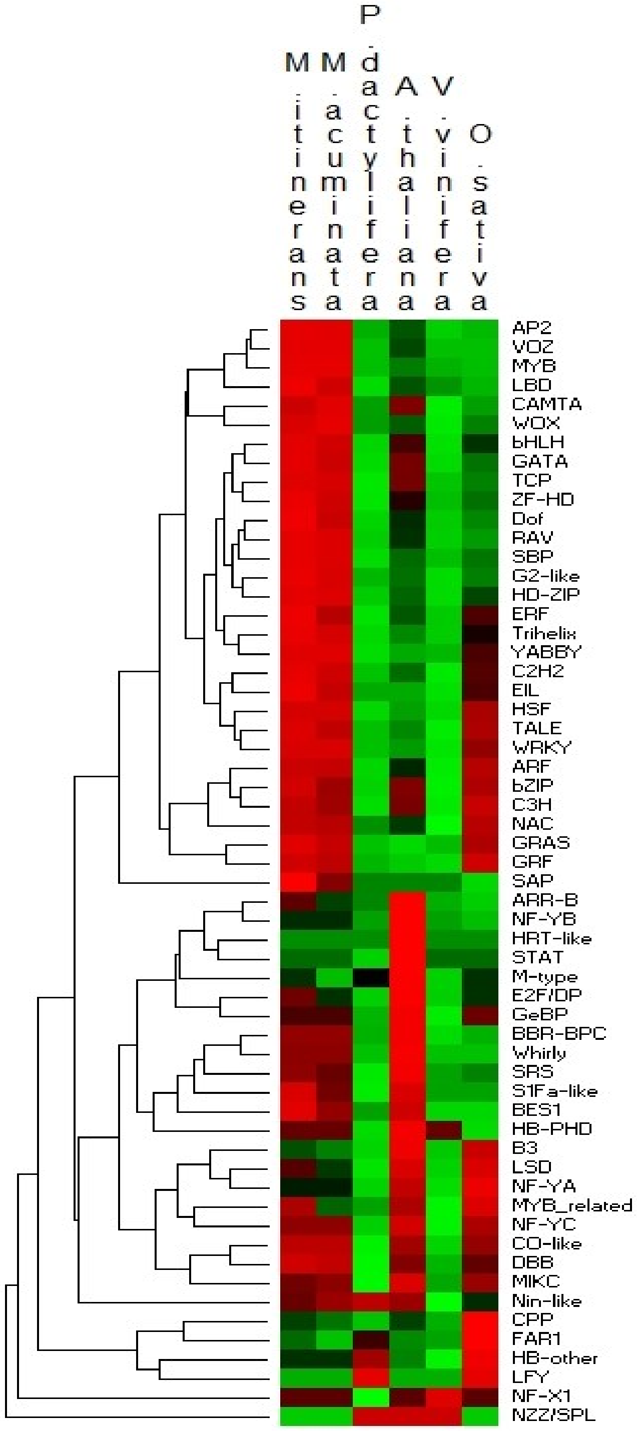

Supplementary Figure 14. Hierarchical clustering of 58 transcriptions factors (TFs) among *Musa itinerans* and other five species. The green and red color code indicates whether a particular TF is under or over-represented (based on z-scores), respectively, in a particular species across the 6 genomes analyzed. Hence, red blocks reflect gene family expansions.

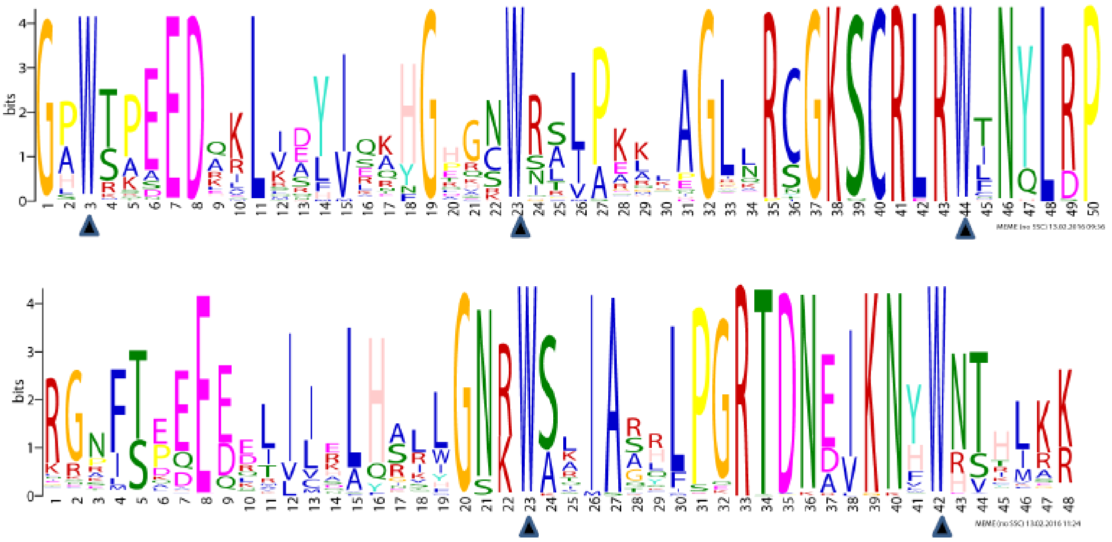

Supplementary Figure 15. DNA-binding domains of R2R3-MYB transcription factor proteins in *Musa acuminata*, and *Musa itinerans*. Above represent the R2 repeats, and bottom represent the R3 repeats of R2R3-MYBs; highly conserved tryptophan amino acids are labeled with solid triangles.

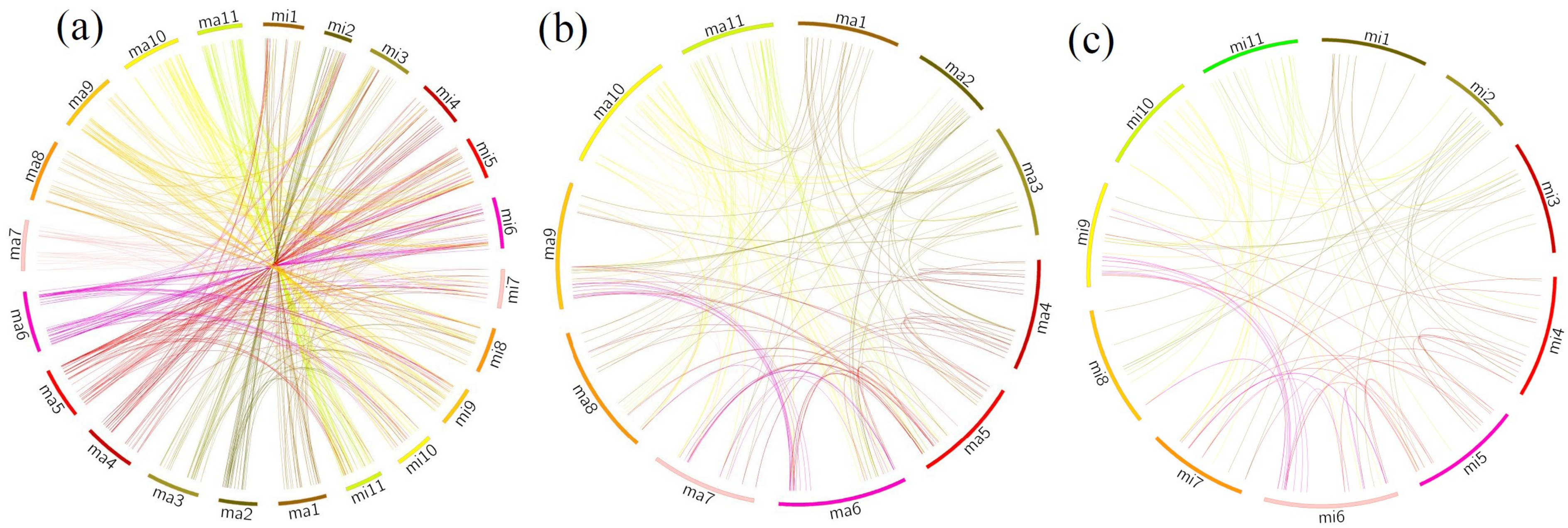

Supplementary Figure 16. Collinearity mapping the orthologs MYB gene pairs between *Musa itinerans* and *M. acuminata*. (a) paralogous MYB gene pairs for *M. acuminata;* (b) and *M. itinerans* (c). the chromosomes of *M. acuminata* were abbreviated as ‘ma’, and M. *itinerans* as ‘mi’; about 52 de novo predicted MYB genes for *M. itinerans* were excluded from the collinearity analysis for unanchored on chromosomes

Supplementary Tables

Supplementary Table 1 Summaries of reads used for *Musa itinerans* genome assembly

Insert size (bp)	Read_length (bp)	Data output(Mb)[a]	Sequencing depth(X)[b]	Physical depth(X)[b]
170	90_90	17984.54/18991.31	29.24	27.62
500	100_100	6166.44/6679.07	10.03	25.07
500	90_90	29527.75/32925.5	48.01	133.37
2000	49_49	7061.49/9709.01	11.48	234.33
5000	49_49	6435.69/10006.69	10.46	533.9
10000	49_49	7051.73/16080.25	11.47	1170.02
Total	-	74227.63/94391.83	120.7	2124.31

[a]Outputs of clean reads after filtering duplicates and reads with low quality , and raw read were separated by slash; [b]The estimation of sequencing depth and physical depth were based on clean data, using a genome size of 615.2Mb.

Supplementary Table 2 Genome size estimation of *Musa itinerans* with the 17-mer analysis.

K	K-mer_number	Peak-depth	Genome size	Used bases	Used reads	Sequencing depth
17	14,764,273,540	24	615,178,064	17,956,548,900	199,517,210	291,892

Supplementary Table 3 Statistics of the assembly of the *Musa itinerans* genome

	Scaffold size (bp)	Scaffold number	Contig size(bp)	Contig number
Maximum size	1,648,447	-	7,822	-
N50	192,092	571	33,903	3,002
N60	130,648	863	23,709	4,499
N70	85,290	1,302	15,294	6,179
N80	47,683	2,026	8,984	10,313
N90	19,491	3,519	3,668	17,421
Total_length	462,139,488	-	422,119,989	-
Number>=100bp		75,687		103,534
Number>=2kb		7,194		22,395

Supplementary Table 4. The completeness of *Musa acuminata* and *Musa itinerans* genome assemblies evaluated with coverage of the conserved 248 core eukaryote gene (CEGs) with CEGMA program.

		Complete CEGs	Prots[2]	%Completeness[3]	Total[4]	Average[5]	%Ortho[6]
M. itinerans	complete		198	79.84	425	2.15	58.59
	Group1	66	51	77.27	89	1.75	43.14
	Group2	56	43	76.79	76	1.77	44.19
	Group3	61	46	75.41	105	2.28	65.22
	Group4	65	58	89.23	155	2.67	77.59
	Partial		234	94.35	589	2.52	70.09
	Group1	66	60	90.91	124	2.07	50
	Group2	56	52	92.86	116	2.23	55.77
	Group3	61	59	96.72	156	2.64	83.05
	Group4	65	63	96.92	193	3.06	88.89
M. acuminata	complete		203	81.85	436	2.15	60.1
	Group1	66	51	77.27	89	1.75	43.14
	Group2	56	43	76.79	79	1.84	41.86
	Group3	61	49	80.33	116	2.37	73.47
	Group4	65	60	92.31	152	2.53	76.67
	Partial		232	93.55	565	2.44	72.84
	Group1	66	59	89.39	118	2	54.24
	Group2	56	49	87.5	110	2.24	63.27
	Group3	61	61	100	159	2.61	83.61
	Group4	65	63	96.92	178	2.83	87.3

[1] Complete: the predicted proteins in the set of 248 CEGs not less than 70% of the alignment length when aligned to the HMM for the KOG for that protein-family

[2] Prots : number of 248 ultra-conserved CEGs present in genome

[3] %Completeness: percentage of 248 ultra-conserved CEGs present

[4] Total: total number of CEGs present including putative orthologs

[5] Average: average number of orthologs per CEG

[6] %Ortho: percentage of detected CEGS that have more than one ortholog

Supplementary Table 5 Comparison of repeat contents in the assembled genomes of *Musa itinerans* and *Musa. acuminata*

Classification	*Musa itinerans*		*Musa acuminata*	
	DNA_length (Mbp)	Percent of genome (%)	DNA_length (Mbp)	Percent of genome(%)
**Classs I: Retrotransposon**	162.88	35.24	157.81	33.37
LTR-Retrotransposon	157.43	34.07	154.75	32.72
Gypsy	75.11	16.25	86.76	18.34
Copia	77.26	16.71	73.86	15.62
other	39.32	8.51	29.38	6.21
Non-LTR Retrotransposon	7.19	1.56	4.69	0.99
LINE	6.68	1.45	4.48	0.95
SINE	0.51	0.11	0.21	0.04
**Class II: DNA** Transposon	14.55	3.15	10.08	2.13
hAT	4.15	0.90	2.48	0.52
CMC	4.48	0.97	4.91	1.04
TcMar	0.52	0.11	0.36	0.11
other	6.65	1.43	3.75	0.79
**Satellite**	0.24	0.05	0.08	0.02
**Simple repeat**	0.6	0.13	0.3	0.06
**Other**	0.003	0	0.006	0
**Unknown**	11.58	2.51	8.14	1.72
total	179.99	38.95	167.59	35.43

Supplementary Table 6 Homology-based gene predication for *Musa itinerans* genome

Gene set		Gene number	Average gene length	Average cds length	Average exon number	Average exon length	Average intron length
Homolog	*Arabidopsis thaliana*	34,958	2931.69	939.41	4.06	231	651.29
	Musa acuminata	49,727	2685.5	864.81	4.05	213	596.7
	Oryza sativa	50,775	2150.9	771.51	3.17	243	635.07
	Phoenix dactylifera	47,543	2045.76	774.47	3.19	243	581.46
	Sorghum bicolor	36,796	2822.91	914.75	3.96	231	644.81
	Zea mays	58,241	1933.62	722.16	2.95	244	620.08
Final gene set	*M.itinerans*	32,456	3608.35	1065.4	5.15	207	613.49

Supplementary Table 7 Gene functional annotations based on different protein database for *Musa itinerans* genome.

	Number	Percent (%)
Total	32,456	
InterPro	23,155	71.34
GO	17,883	55.10
KEGG	17,365	53.50
Swissprot	21,958	67.65
TrEMBL	28,048	86.42
Annotated	28,231	86.98
Unannotated	4,225	13.02

Supplementary Table 8 Non-coding RNA genes in the *Musa itinerans* genome.

Type		Copy(w)	Average length(bp)	Total length(bp)	% of genome
miRNA		345	116.4	40,159	0.0087
tRNA		977	75.4	73,657	0.0159
rRNA		278	194.2	53,994	0.0117
	18S	42	748.5	31,437	0.0068
	28S	54	117.9	6,368	0.0014
	5.8S	13	117.5	1,528	0.0003
	5S	169	86.8	14,661	0.0032
snRNA	snRNA	299	120.7	36,081	0.0078
	CD-box	170	103.2	17,540	0.0038
	HACA-box	18	121.9	2,194	0.0005
	splicing	111	147.3	16,347	0.0035

Supplementary Table 9. Gene family clustering among *Musa itinerans* and other eight related species

Species	Genes number	Genes in families	Unclustered genes	Family number	Unique families	Average genes per family
Arabidopsis thaliana	26,637	23,036	3,601	12,334	889	1.87
Musa acuminata	34,239	29,222	5,017	18,564	88	1.57
Musa balbisiana	36,037	30,369	5,668	15,827	274	1.92
Oryza sativa	35,402	24,698	10,704	15,867	1,191	1.56
Phoenix dactylifera	24,838	22,278	2,560	13,221	167	1.69
Sorghum bicolor	27,159	23,284	3,875	15,068	416	1.55
Vitis vinifera	25,329	19,311	6,018	12,144	695	1.59
Elaeis guineensis	28,336	20,534	7,802	11,966	351	1.72
Musa itinerans	28,524	26,955	1,569	16,742	177	1.61

Supplementary Table 10. Gene ontology (GO) enrichment analysis of Musa itinerans unique gene families among five species[a] and shown for significantly[b] represented GO terms.

GO_id	GO terms	Number of enriched genes	Number of genes in background	P-value
GO:0034061	DNA polymerase activity	8	30	1.56E-09
GO:0016891	endoribonuclease activity, producing 5'-phosphomonoesters	7	21	5.12E-09
GO:0016893	endonuclease activity, active with either ribo- or deoxyribonucleic acids and producing 5'-phosphomonoesters	7	22	7.47E-09
GO:0004521	endoribonuclease activity	7	25	2.07E-08
GO:0004519	endonuclease activity	7	35	2.74E-07
GO:0004540	ribonuclease activity	7	37	4.15E-07
GO:0003676	nucleic acid binding	31	1628	4.01E-06
GO:0006260	DNA replication	8	70	4.61E-06
GO:0060089	molecular transducer activity	9	142	4.48E-05
GO:0044391	ribosomal subunit	13	311	5.28E-05
GO:0005840	ribosome	15	481	0.00
GO:0015934	large ribosomal subunit	9	172	0.00
GO:0004871	signal transducer activity	7	112	0.00
GO:0032991	macromolecular complex	28	1579	0.00
GO:0016779	nucleotidyltransferase activity	8	161	0.00
GO:0006259	DNA metabolic process	13	440	0.00
GO:1901360	organic cyclic compound metabolic process	34	2501	0.00
GO:0090304	nucleic acid metabolic process	26	1608	0.00
GO:0004518	nuclease activity	7	123	0.00
GO:0034641	cellular nitrogen compound metabolic process	32	2300	0.00
GO:0046483	heterocycle metabolic process	32	2314	0.00
GO:0051716	cellular response to stimulus	19	993	0.00
GO:0043228	non-membrane-bounded organelle	19	929	0.01
GO:0043232	intracellular non-membrane-bounded organelle	19	929	0.01
GO:0006725	cellular aromatic compound metabolic process	32	2414	0.01
GO:0007154	cell communication	17	839	0.01
GO:0006807	nitrogen compound metabolic process	34	2693	0.01
GO:0007165	signal transduction	15	680	0.01
GO:0023052	signaling	15	680	0.01

GO:0044700	single organism signaling	15	680	0.01
GO:0034645	cellular macromolecule biosynthetic process	21	1239	0.01
GO:0006139	nucleobase-containing compound metabolic process	29	2106	0.01
GO:0001071	nucleic acid binding transcription factor activity	9	285	0.01
GO:0009059	macromolecule biosynthetic process	21	1268	0.01
GO:0043170	macromolecule metabolic process	46	4384	0.01
GO:0030529	ribonucleoprotein complex	15	711	0.02
GO:0005198	structural molecule activity	12	550	0.03
GO:0044249	cellular biosynthetic process	27	2015	0.03
GO:0034518	RNA cap binding complex	2	5	0.04
GO:1901576	organic substance biosynthetic process	27	2044	0.04
GO:0010467	gene expression	25	1828	0.05

[a] Five species including *Musa itinerans*, *Musa acuminata, Musa babisiana, Phoenix dactylifera, Elaeis guineensis,*

[b] The Bonferroni correction of the significance level of hypergeometric test set threshold P value as 0.05

Supplementary Table 11. KEGG pathway enrichment analysis of *Musa itinerans* unique gene families among five species[a] and shown for significantly[b] represented pathways

Pathway	Number of enriched genes (82)	Number of genes in background (16517)	P-value	Pathway ID
Ribosome	16	584	2.33E-08	ko03010
Proteasome	6	110	1.77E-05	ko03050
Plant hormone signal transduction	14	1151	0.00	ko04075
Inositol phosphate metabolism	4	135	0.00	ko00562
Ether lipid metabolism	2	41	0.02	ko00565
Fatty acid biosynthesis	2	60	0.04	ko00061

[a] Five species including *Musa itinerans, Musa acuminata, Musa babisiana, Phoenix dactylifera, Elaeis guineensis.*

[b] The Bonferroni correction of the significance level of hypergeometric test set threshold P value as 0.05

Supplementary Table 12. KEGG pathway enrichment analysis of expanded gene families for ancestral *Musa* species among nine species[a] and shown for significantly[b] represented pathways

Pathway	Number of enriched genes(1086)	Number of genes in background (16517)	P-value	Pathway ID
Phagosome	61	225	2.18E-20	ko04145
Plant-pathogen interaction	149	1020	1.11E-19	ko04626
Flavonoid biosynthesis	47	154	2.29E-18	ko00941
Glycosphingolipid biosynthesis - ganglio series	20	32	3.11E-15	ko00604
Flavone and flavonol biosynthesis	28	76	1.62E-13	ko00944
Glycosaminoglycan degradation	20	52	3.26E-10	ko00531
Cysteine and methionine metabolism	33	137	3.89E-10	ko00270
Galactose metabolism	29	110	4.91E-10	ko00052
Linoleic acid metabolism	13	32	2.90E-07	ko00591
Ascorbate and aldarate metabolism	27	136	1.21E-06	ko00053
alpha-Linolenic acid metabolism	21	93	2.67E-06	ko00592
Sphingolipid metabolism	20	89	4.96E-06	ko00600
Inositol phosphate metabolism	24	135	3.34E-05	ko00562
Phenylpropanoid biosynthesis	38	280	7.06E-05	ko00940
Monoterpenoid biosynthesis	9	25	7.06E-05	ko00902
Stilbenoid, diarylheptanoid and gingerol biosynthesis	21	117	8.59E-05	ko00945
Isoflavonoid biosynthesis	7	19	4.67E-04	ko00943
Other glycan degradation	20	123	4.98E-04	ko00511
Phosphatidylinositol signaling system	24	163	5.35E-04	ko04070
Amino sugar and nucleotide sugar metabolism	34	270	6.15E-04	ko00520
Tryptophan metabolism	14	74	8.37E-04	ko00380
Limonene and pinene degradation	15	90	2.03E-03	ko00903

Diterpenoid biosynthesis	12	69	4.39E-03	ko00904
Biosynthesis of secondary metabolites	157	1921	5.04E-03	ko01110
ABC transporters	22	181	9.51E-03	ko02010
Metabolic pathways	280	3783	2.69E-02	ko01100
Starch and sucrose metabolism	43	463	3.08E-02	ko00500
Phenylalanine metabolism	18	156	3.08E-02	ko00360
Plant hormone signal transduction	94	1151	3.36E-02	ko04075

[a] Nine species used in OrthoMCL clustering including *Musa itinerans, Musa acuminata, Musa balbisiana, Phoenix dactylifera, Elaeis guineensis, Sorghum bicolor, Oryza sativa*, *Vitis vinifera, Arabidopsis thaliana*

[b] The Bonferroni correction of the significance level of hypergeometric test set threshold P value as 0.05

Supplementary Table 13. KEGG pathway enrichment analysis of contracted ancestral *Musa* gene families among nine species[a] and shown for significantly[b] represented pathways

Pathway	Number of enriched genes(900)	Number of genes in background (16517)	P-value	Pathway ID
Phenylalanine metabolism	26	156	3.44E-05	ko00360
Isoquinoline alkaloid biosynthesis	10	34	4.73E-04	ko00950
Phenylpropanoid biosynthesis	32	280	2.21E-03	ko00940
Ribosome	54	584	2.71E-03	ko03010
Tropane, piperidine and pyridine alkaloid biosynthesis	7	25	6.14E-03	ko00960
Ubiquinone and other terpenoid-quinone biosynthesis	11	64	1.07E-02	ko00130
Other types of O-glycan biosynthesis	6	21	1.07E-02	ko00514
Zeatin biosynthesis	11	67	1.25E-02	ko00908
Protein processing in endoplasmic reticulum	41	474	2.56E-02	ko04141
Tyrosine metabolism	11	75	2.56E-02	ko00350
Galactose metabolism	14	110	2.56E-02	ko00052
Glycosphingolipid biosynthesis - globo series	5	19	2.64E-02	ko00603
Protein export	11	80	3.31E-02	ko03060
Cyanoamino acid metabolism	12	95	4.09E-02	ko00460
Pyruvate metabolism	16	145	4.09E-02	ko00620

[a] Nine species used in OrthoMCL clustering including *Musa itinerans, Musa acuminata, Musa balbisiana, Phoenix dactylifera, Elaeis guineensis, Sorghum bicolor, Oryza sativa*, *Vitis vinifera, Arabidopsis thaliana*

[b] The Bonferroni correction of the significance level of hypergeometric test set threshold P value as 0.05

Supplementary Table 14. KEGG pathway enrichment analysis of *Musa itinerans* expanded gene families among nine species[a] and shown for significantly[b] represented pathways.

Pathway	Number of enriched genes (594)	Number of genes in background (16517)	P-value	Pathway ID
Phagosome	50	225	6.61E-24	ko04145
Amino sugar and nucleotide sugar metabolism	40	270	8.13E-13	ko00520
Biosynthesis of secondary metabolites	130	1921	8.33E-12	ko01110
Ribosome	53	584	9.98E-09	ko03010
Flavone and flavonol biosynthesis	16	76	1.37E-07	ko00944
Cysteine and methionine metabolism	19	137	5.59E-06	ko00270
Flavonoid biosynthesis	19	154	2.91E-05	ko00941
Pentose and glucuronate interconversions	19	193	6.44E-04	ko00040
Metabolic pathways	174	3783	1.12E-03	ko01100
Starch and sucrose metabolism	33	463	1.12E-03	ko00500
Zeatin biosynthesis	9	67	4.18E-03	ko00908
Glycolysis / Gluconeogenesis	18	221	6.59E-03	ko00010
Carbon fixation in photosynthetic organisms	13	136	7.02E-03	ko00710
Tyrosine metabolism	9	75	7.13E-03	ko00350
Biosynthesis of unsaturated fatty acids	8	61	7.13E-03	ko01040
Glucosinolate biosynthesis	4	15	7.42E-03	ko00966
Tropane, piperidine and pyridine alkaloid biosynthesis	5	25	7.42E-03	ko00960
Protein export	9	80	9.17E-03	ko03060
Photosynthesis	12	130	9.78E-03	ko00195
Fructose and mannose metabolism	11	123	1.76E-02	ko00051
Isoquinoline alkaloid biosynthesis	5	34	2.41E-02	ko00950

[a] Nine species used in OrthoMCL clustering including *Musa itinerans, Musa acuminata,Musa balbisiana, Phoenix dactylifera, Elaeis guineensis, Sorghum bicolor, Oryza sativa*, *Vitis vinifera, Arabidopsis thaliana*

[b] The Bonferroni correction of the significance level of hypergeometric test set threshold P value as 0.05

Supplementary Table 15. KEGG pathway enrichment analysis of *Musa itinerans* contracted gene families among nine species[a] and shown for significantly [b] represented pathways

Pathway	Number of enriched genes (876)	Number of genes in background (16517)	P-value	Pathway
ABC transporters	46	181	1.25E-17	ko02010
Glycosphingolipid biosynthesis - ganglio series	20	32	1.08E-16	ko00604
Glycosaminoglycan degradation	20	52	1.47E-11	ko00531
Plant-pathogen interaction	103	1020	2.28E-09	ko04626
Linoleic acid metabolism	13	32	4.57E-08	ko00591
Sphingolipid metabolism	21	89	6.36E-08	ko00600
Other glycan degradation	24	123	2.50E-07	ko00511
Galactose metabolism	20	110	1.03E-05	ko00052
Limonene and pinene degradation	17	90	3.34E-05	ko00903
Ether lipid metabolism	11	41	4.47E-05	ko00565
Stilbenoid, diarylheptanoid and gingerol biosynthesis	19	117	7.68E-05	ko00945
Inositol phosphate metabolism	20	135	1.63E-04	ko00562
Endocytosis	28	228	1.63E-04	ko04144
RNA polymerase	22	160	1.92E-04	ko03020
Ascorbate and aldarate metabolism	19	136	4.77E-04	ko00053
Tryptophan metabolism	13	74	5.43E-04	ko00380
Flavone and flavonol biosynthesis	13	76	6.76E-04	ko00944
Phosphatidylinositol signaling system	20	163	1.57E-03	ko04070

[a] Nine species used in OrthoMCL clustering including *Musa itinerans, Musa acuminata, Musa balbisiana, Phoenix dactylifera, Elaeis guineensis, Sorghum bicolor, Oryza sativa*, *Vitis vinifera, Arabidopsis thaliana*

[b] The Bonferroni correction of the significance level of hypergeometric test set threshold P value as 0.05

Supplementary Table 16. Overview of *Musa itinerans* pseudo-chromosomes based on syntenic blocks between *M. itinerans* and *Musa acuminata*

Pseudo chromosome	Number of Scaffolds	Total size of scaffold(bp）	Number of genes
1	96	23,618,511	2,271
2	46	16,378,154	1,698
3	78	25,367,131	2,540
4	100	27,186,878	2,742
5	84	24,886,606	2,332
6	105	29,658,729	2,947
7	107	22,363,963	2,171
8	108	26,494,654	2,605
9	93	23,201,293	2,247
10	80	22,470,544	2,345
11	97	21,913,503	2,106
Un_random	49	9,706,350	666
Total	1,043	273,246,316	26,670
M.itinerans assembly	75,687	462,139,488	32,356

Supplementary Table 17 Classifications* of NBS-containing Resistances in three *Musa* species *M. acuminata*, *M. balbisiana*, and *M. itinerans*.

	M. acuminata	*M. balbisiana*	*M. itinerans*
NBS（>95aa)	117(99)	93(82)	62(57)
(NBS)n(n>1)	20	19	8
NBS-LRR1	80	69	50
NBS-LRR2	0	0	0
NBS-LRR3	1	0	1
CC-NBS	30	34	20
TIR-NBS	0	0	0
Number of singletons	40	47	32
Number of multiple genes	35	12	12
Number of gene families*	10	4	4
Average members per gene family	3.5	3	3
Proportion of multi-gene	0.467	0.203	0.273

*Gene family classification determined by identity > 70% with the 140 amino acids standard NB-ARC Pfam domain (PF00931)

Supplementary Table 18 Genome-wide duplication modes and ages of NBS-encoding genes in *Musa itinerans*

Duplicate1	Duplicate2	Mode	*Ka*	*Ks*	*Ka/Ks*
Mi_g021494	Mi_g021491	proximal	0.61	1.27	0.48
Mi_g023761	Mi_g023763	proximal	0	0.01	0.17
Mi_g000507	Mi_g000689	segmental	0.32	0.45	0.72
Mi_g010079	Mi_g019630	segmental	0.06	0.53	0.11
Mi_g015709	Mi_g011020	segmental	0.32	0.99	0.32
Mi_g004238	Mi_g026403	segmental	0.62	1.08	0.57
Mi_g021494	Mi_g003639	segmental	0.56	1.88	0.3
Mi_g000382	Mi_g031777	segmental	0.24	0.87	0.27
Mi_g005793	Mi_g000507	transposed	0.43	0.84	0.51
Mi_g018211	Mi_g005793	transposed	0.41	0.85	0.48
Mi_g024483	Mi_g001472	transposed	0.54	2.43	0.22
Mi_g008673	Mi_g000689	transposed	0.38	0.86	0.44
Mi_g018211	Mi_g008673	transposed	0.38	1.02	0.37
Mi_g023244	Mi_g008031	transposed	-	-	-
Mi_g000496	Mi_g001472	transposed	1	3.68	0.27
Mi_g011001	Mi_g008031	transposed	0.73	2.01	0.36
Mi_g007030	Mi_g001419	transposed	0.79	2.17	0.36
Mi_g001419	Mi_g026403	transposed	0.62	1.22	0.5
Mi_g023807	Mi_g004238	transposed	0.65	1	0.64
Mi_g014071	Mi_g004238	transposed	0.82	0.99	0.83
Mi_g007030	Mi_g008031	transposed	0.69	1.99	0.35
Mi_g012577	Mi_g012553	transposed	1.13	1.35	0.84
Mi_g007832	Mi_g026403	transposed	0.71	2.28	0.31
Mi_g020550	Mi_g004238	transposed	0.65	1.78	0.37
Mi_g008262	Mi_g021494	transposed	0.69	1.98	0.35
Mi_g017994	Mi_g000382	transposed	0.9	5.5	0.16
Mi_g003578	Mi_g008031	transposed	1.04	2.24	0.46
Mi_g002312	Mi_g008031	transposed	1.25	2.1	0.6
Mi_g018211	Mi_g000689	transposed	0.36	1.14	0.32

.

Supplementary Table 19 Genome-wide duplication modes and ages NBS-encoding genes in *Musa acuminata*.

Duplicate1[a]	Duplicate2	Mode	Ks	Ka	Ka/Ks
1P08690_001	1P08716_001	proximal	0.13	0.16	0.79
10P07050_001	10P07065_001	proximal	0.09	0.02	4.72
9P02760_001	9P02800_001	proximal	0.62	1.72	0.36
3P27810_001	3P27830_001	proximal	0.12	0.16	0.74
3P22300_001	3P22320_001	proximal	0.14	0.08	1.72
10P07080_001	10P07120_001	proximal	0.05	0.09	0.58
6P32040_001	6P32070_001	proximal	0.63	1.91	0.33
10P29330_001	7P21400_001	segmental	0.23	1.09	0.22
3P27790_001	9P23050_001	segmental	0.35	0.93	0.38
3P10930_001	9P19800_001	segmental	0.06	0.51	0.12
8P03665_001	9P02730_001	segmental	0.56	0.99	0.56
10P07050_001	11P07440_001	segmental	0.26	0.8	0.33
8P25780_001	9P28020_001	segmental	0.76	2.39	0.32
8P25780_001	9P12800_001	segmental	-	-	-
5P04240_001	9P12800_001	segmental	0.26	0.82	0.31
6P32040_001	8P21580_001	segmental	0.61	1.48	0.41
1P12540_001	1P12550_001	tandem	0.07	0.07	1.14
1P12550_001	1P12560_001	tandem	0.12	0.14	0.82
9P02730_001	9P02735_001	tandem	0.11	0.24	0.47
3P27810_001	3P27817_001	tandem	0.25	0.1	2.41
6P32040_001	6P32050_001	tandem	1.21	0.47	2.56
9P02760_001	9P02765_001	tandem	0.25	1.04	0.25
10P07100_001	10P07120_001	tandem	0.21	0.63	0.34
1P14020_001	1P14030_001	tandem	0.06	0.12	0.49
3P27790_001	3P27810_001	tandem	0.11	0.13	0.8
3P22300_001	3P22310_001	tandem	0.11	0.13	0.81
3P22250_001	3P22260_001	tandem	0.1	0.12	0.83
10P24920_001	10P24940_001	tandem	0.15	0.12	1.24
10P24910_001	10P24920_001	tandem	0.14	0.18	0.76
10P07140_001	10P07150_001	tandem	0.07	0.16	0.42
9P28020_001	9P28030_001	tandem	0.11	0.15	0.75
3P22280_001	3P22290_001	tandem	0.15	0.2	0.78
7P20230_001	7P20240_001	tandem	0.08	0.07	1.27
7P20220_001	7P20230_001	tandem	0.07	0.09	0.73
5P19520_001	9P19800_001	transposed	-	-	-
7P12770_001	7P21400_001	transposed	1.25	2.22	0.57
1P04610_001	9P19800_001	transposed	0.64	3.93	0.16
9P02460_001	8P03665_001	transposed	0.63	1.17	0.54
3P28030_001	3P27790_001	transposed	0.11	0.13	0.84
2P11320_001	11P07440_001	transposed	0.53	1.79	0.3
0P25090_001	11P07440_001	transposed	0.54	0.22	2.46

0P25100_001	10P07050_001	transposed	0.61	0.18	3.42
9P20610_001	10P07050_001	transposed	0.38	1.02	0.37
0P27050_001	3P27790_001	transposed	0.1	0.11	0.98
9P11390_001	3P09290_001	transposed	0.81	1.28	0.63
6P08580_001	9P17060_001	transposed	0.55	1.28	0.43
6P34690_001	6P21800_001	transposed	0.24	0.63	0.39
4P31030_001	6P21800_001	transposed	0.61	1.83	0.34
4P31030_001	9P11660_001	transposed	0.61	1.91	0.32
5P11640_001	10P29330_001	transposed	0.67	2.7	0.25
9P17310_001	10P29330_001	transposed	0.68	2.59	0.26
11P18580_001	9P11660_001	transposed	0.77	0.86	0.9
6P21780_001	6P19710_001	transposed	0.56	1.2	0.47
0P28160_001	3P22250_001	transposed	0.11	0.09	1.25
0P28220_001	3P22250_001	transposed	0.18	0.13	1.32
0P26110_001	3P22250_001	transposed	0.16	0.08	1.99
6P29800_001	5P04240_001	transposed	0.79	2.65	0.3
9P02520_001	4P28700_001	transposed	0.83	1.3	0.64
8P30710_001	9P12800_001	transposed	0.74	1.39	0.53
0P28310_001	3P27790_001	transposed	0.2	0.05	4.2
11P25110_001	10P29330_001	transposed	–	-	–

[a] The name of *Musa acuminata* duplicated genes were abbreviated by dropping off the string of 'GSMUA_Achr' compared with the original gene name.

Supplementary Table 20. Transcription factors across two *Musa* genomes and other related species

TF family	Blast	Homolog search	*Musa itinerans*	*Musa acuminata*	*Phoenix dactylifera*_v3	*Arabidopsis thaliana*	*Vitis vinifera*	*Oryza sativa*
AP2	47	3	**50**	**49**	23	30	19	22
ARF	42	9	**51**	**50**	22	37	17	48
ARR-B	12	3	**15**	**14**	13	21	12	11
B3	27	17	**44**	**40**	26	77	29	65
BBR-BPC	9	3	**12**	**12**	7	17	5	7
BES1	11	4	**15**	**12**	8	14	6	6
bHLH	249	71	**320**	**298**	128	225	115	211
bZIP	108	48	**156**	**135**	68	127	47	140
C2H2	120	69	**189**	**174**	88	116	64	135
C3H	54	19	**73**	**69**	46	66	43	74
CAMTA	8	4	**12**	**13**	7	10	4	7
CO-like	19	5	**24**	**24**	1	22	6	21
CPP	8	1	**9**	**8**	5	9	6	20
DBB	12	6	**18**	**17**	1	14	7	13
Dof	53	37	**90**	**74**	19	47	22	37
E2F/DP	9	2	**11**	**10**	7	16	7	10
EIL	18	2	**20**	**16**	6	6	2	11
ERF	123	145	**268**	**209**	55	139	80	163
FAR1	19	14	**33**	**6**	48	26	18	133
G2-like	94	16	**110**	**102**	51	64	40	62
GATA	37	19	**56**	**51**	19	41	19	32
GeBP	6	6	**12**	**12**	6	23	1	13

GRAS	58	21	**79**	**73**	42	37	43	69
GRF	15	4	**19**	**18**	10	9	8	19
HB-other	9	3	**12**	**12**	14	11	7	17
HB-PHD	2		**2**	**2**	1	3	2	1
HD-ZIP	89	10	**99**	**95**	38	58	33	61
HRT-like	1		**1**	**1**	1	2	1	1
HSF	28	15	**43**	**43**	19	25	19	38
LBD	66	15	**81**	**72**	31	50	44	39
LFY	1		**1**	**1**	2	1	1	2
LSD	6	2	**8**	**7**	2	12	3	12
MIKC	55	1	**56**	**59**	10	76	36	61
M-type	20	15	**35**	**22**	36	70	18	35
MYB	249	55	**304**	**303**	129	168	138	130
MYB_related	76	21	**97**	**82**	76	97	57	106
NAC	153	21	**174**	**167**	124	138	71	170
NF-X1	2		**2**	**2**		2	3	2
NF-YA	15		**15**	**15**	8	21	7	25
NF-YB	18	1	**19**	**19**	17	27	17	16
NF-YC	17	1	**18**	**18**	11	21	8	19
Nin-like	16		**16**	**17**	18	17	8	15
NZZ/SPL	0	0	**0**	**0**	1	1	1	0
RAV	14	2	**16**	**15**	1	7	1	4
S1Fa-like	1	3	**4**	**3**	1	4	2	2
SAP	3	1	**4**	**2**	1	1	1	0
SBP	55	6	**61**	**58**	12	30	19	29
SRS	7	3	**10**	**9**	1	16	5	6

STAT	1	0	**1**	**1**	0	4	1	1
TALE	39	12	**51**	**47**	28	33	21	45
TCP	26	21	**47**	**45**	8	33	15	23
Trihelix	28	32	**60**	**55**	24	34	26	40
VOZ	4	1	**5**	**5**	2	3	2	2
Whirly	3	0	**3**	**3**	2	4	2	2
WOX	21	5	**26**	**27**	16	18	11	17
WRKY	122	31	**153**	**153**	78	90	59	128
YABBY	23	2	**25**	**25**	3	8	7	15
ZF-HD	16	15	**31**	**27**	5	18	10	15
**Total**	2343	833	**3176**	**2898**	1426	2296	1276	2408

Supplementary Table 21 List of MYB transcripton factor genes for two *Musa* species

Musa acuminata			***Musa itinerans***		
MYB genes	position of domain	Type	MYB genes	positions of domain	Type
10P19840_001[a]	18 67[b]	1R	5P17010_001_D1[c]	14 63	1R
11P07660_001	14 64	1R	Mi_g009970	5 54	1R
4P06410_001	65 112	1R	Mi_g008629	14 64	1R
4P13280_001	64 111	1R	Mi_g005375	14 64	1R
5P07380_001	25 87	1R	4P06410_001_D1	65 112	1R
5P10690_001	14 64	1R	5P07380_001_D1	24 86	1R
5P17010_001	14 63	1R	5P21840_001_D1	14 114	1R
6P30830_001	14 64	1R	Mi_g005112	21 69	1R
7P21550_001	41 90	1R	Mi_g017587	14 64	1R
8P18460_001	5 54	1R	Mi_g007855	41 90	1R
9P17000_001	87 135	1R	8P14190_001_D2	34 80	1R
Un_randomP10790_001	14 64	1R	Mi_g000673	13 63	1R
Un_randomP21150_001	17 92	1R	Mi_g000872	14 64	1R
Un_randomP22350_001	70 117	1R	Mi_g012547	35 83	1R
10P14840_001	41 89 93 141 145 192	3R	Mi_g029930	67 115 119 167 171 218	R1R2R3
10P26610_001	67 115 119 167 171 218	3R	Mi_g008651	36 84 88 136 140 187	R1R2R3
2P03880_001	36 84 88 136 140 187	3R	6P05030_001_D2	56 104 108 156	R1R2R3

				160 207	
2P19890_001	47 95 99 147 151 198	3R	Mi_g020828	319 365 371 418 423 467	R1R2R3
3P01370_001	5 54 22 71 75 118	3R	Mi_g001538	794 842 846 894 898 945	R1R2R3
6P05030_001	56 104 108 156 160 207	3R	10P19970_001_D1	14 63 67 114	R2R3
6P31410_001	340 386 392 439 444 488	3R	10P26730_001_D1	8 57 61 108	R2R3
7P10520_001	795 843 847 895 899 946	3R	Mi_g017415	187 235 242 286	R2R3
9P10150_001	172 219 223 271 277 324 329 373	4R	Mi_g025663	14 63 68 114	R2R3
10P00770_001	184 232 239 283	R2R3	Mi_g005920	14 63 67 114	R2R3
10P01620_001	14 63 67 114	R2R3	Mi_g000908	31 77 136 185	R2R3
10P01640_001	14 63 67 114	R2R3	Mi_g015857	39 88 97 139	R2R3
10P02290_001	31 77 136 185	R2R3	Mi_g007998	39 88 97 139	R2R3
10P03710_001	10 59 63 110	R2R3	Mi_g011792	26 74 78 125	R2R3
10P04590_001	51 100 104 151	R2R3	Mi_g017225	14 63 67 112	R2R3
10P05080_001	63 111 115 162	R2R3	Mi_g012174	14 68 72 119	R2R3
10P06880_001	11 59 80 127	R2R3	Mi_g009075	14 78 82 129	R2R3
10P08070_001	14 63 67 114	R2R3	Mi_g016283	14 63 67 114	R2R3
10P08350_001	14 64 71 115	R2R3	Mi_g008837	15 64 68 115	R2R3
10P10800_001	19 67 71 118	R2R3	Mi_g011643	14 63 67 114	R2R3
10P12830_001	78 126 141 177	R2R3	Mi_g005860	8 57 61 108	R2R3
10P13520_001	15 64 68 115	R2R3	Mi_g031435	6 55 59 106	R2R3
10P14030_001	39 88 97 139	R2R3	Mi_g016052	14 63 68 114	R2R3
10P16010_001	14 63 67 114	R2R3	Mi_g012551	14 63 67 114	R2R3
10P17610_001	14 63 67 112	R2R3	Mi_g026137	14 63 71 114	R2R3

10P18880_001	29 75 79 126	R2R3	Mi_g028484	14 63 71 114	R2R3
10P19130_001	14 63 67 114	R2R3	Mi_g013052	14 63 71 114	R2R3
10P19970_001	14 63 67 114	R2R3	Mi_g010260	14 63 71 114	R2R3
10P24560_001	14 63 67 114	R2R3	Mi_g030451	14 63 71 114	R2R3
10P25700_001	14 68 72 119	R2R3	11P07850_001_D1	15 64 68 115	R2R3
10P26730_001	8 57 61 108	R2R3	11P13080_001_D1	14 63 67 114	R2R3
10P29210_001	68 114 14 63	R2R3	11P13200_001_D2	18 67 71 114	R2R3
10P29280_001	63 127 10 59	R2R3	11P22820_001_D1	14 63 67 114	R2R3
10P29680_001	14 63 67 114	R2R3	Mi_g020713	14 63 67 114	R2R3
10P29970_001	71 114 14 63	R2R3	Mi_g006153	14 63 67 114	R2R3
11P00410_001	14 63 67 114	R2R3	Mi_g007232	53 101 105 152	R2R3
11P02410_001	17 66 70 117	R2R3	Mi_g017949	48 96 100 147	R2R3
11P04090_001	14 63 67 114	R2R3	Mi_g005370	18 73 77 120	R2R3
11P04930_001	15 64 68 111	R2R3	Mi_g015986	18 67 71 114	R2R3
11P07180_001	14 63 67 114	R2R3	Mi_g005722	5 54 58 101	R2R3
11P07850_001	15 64 68 115	R2R3	Mi_g031532	16 65 69 116	R2R3
11P09110_001	12 61 75 124	R2R3	Mi_g012944	23 72 76 119	R2R3
11P09160_001	53 101 105 152	R2R3	Mi_g028165	22 71 76 122	R2R3
11P10490_001	91 139 143 190	R2R3	Mi_g016435	33 82 87 133	R2R3
11P13080_001	14 63 67 114	R2R3	1P09180_001_D1	14 63 67 114	R2R3
11P13200_001	18 73 77 120	R2R3	1P22380_001_D1	14 63 71 114	R2R3
11P15910_001	14 63 67 114	R2R3	1P26140_001_D1	15 63 68 114	R2R3
11P16130_001	19 68 72 115	R2R3	Mi_g015151	41 90 97 142	R2R3
11P17190_001	69 116 16 65	R2R3	Mi_g019853	16 65 69 116	R2R3
11P17870_001	16 65 69 116	R2R3	Mi_g020932	5 54 58 105	R2R3
11P20830_001	23 72 76 119	R2R3	Mi_g029097	10 59 63 110	R2R3

11P22820_001	14 63 67 114	R2R3	Mi_g009254	14 64 71 115	R2R3
11P23430_001	14 63 67 114	R2R3	Mi_g029043	17 65 70 116	R2R3
11P23500_001	18 67 71 118	R2R3	Mi_g016194	11 59 118 165	R2R3
11P24690_001	28 77 82 128	R2R3	Mi_g020057	7 56 60 103	R2R3
11P25100_001	38 86 90 137	R2R3	Mi_g028580	14 63 67 114	R2R3
1P01660_001	39 88 95 140	R2R3	Mi_g031463	14 63 67 114	R2R3
1P02420_001	14 63 67 114	R2R3	Mi_g022835	15 64 68 111	R2R3
1P03140_001	14 64 69 115	R2R3	2P01830_001_D1	14 63 67 114	R2R3
1P03450_001	16 65 69 116	R2R3	2P11450_001_D3	15 64 69 115	R2R3
1P04910_001	14 64 71 115	R2R3	Mi_g013983	27 76 80 127	R2R3
1P07550_001	51 100 104 151	R2R3	Mi_g022486	21 70 74 121	R2R3
1P09180_001	14 63 67 114	R2R3	Mi_g022485	21 70 74 121	R2R3
1P09750_001	17 65 70 116	R2R3	Mi_g026023	13 62 66 113	R2R3
1P17150_001	71 120 124 171	R2R3	Mi_g015591	14 63 67 114	R2R3
1P17480_001	5 54 58 122	R2R3	Mi_g023573	15 64 109 141	R2R3
1P17730_001	11 59 118 165	R2R3	Mi_g018991	15 64 68 134	R2R3
1P18610_001	14 63 71 114	R2R3	Mi_g028414	15 64 68 138	R2R3
1P21020_001	18 67 71 114	R2R3	Mi_g031056	14 63 67 114	R2R3
1P23990_001	14 63 67 114	R2R3	Mi_g011516	20 69 73 120	R2R3
1P24960_001	17 66 70 113	R2R3	Mi_g011505	16 65 69 116	R2R3
1P26140_001	15 63 68 114	R2R3	Mi_g013106	16 65 69 116	R2R3
1P26520_001	14 63 67 114	R2R3	3P01370_001_D24	5 54 58 90	R2R3
1P27270_001	15 64 68 111	R2R3	3P07950_001_D1	15 64 68 115	R2R3
2P01830_001	14 63 67 114	R2R3	3P09450_001_D1	14 63 67 114	R2R3
2P02970_001	14 63 67 114	R2R3	3P18970_001_D1	18 66 70 117	R2R3
2P04200_001	14 64 69 115	R2R3	3P21000_001_D1	77 126 130 177	R2R3

2P04730_001	15 64 68 115	R2R3	3P21060_001_D1	18 67 71 114	R2R3
2P07780_001	27 76 80 127	R2R3	3P22200_001_D4	14 63 67 114	R2R3
2P11450_001	15 73 80 143	R2R3	3P28230_001_D1	5 53 58 104	R2R3
2P14770_001	14 63 67 111	R2R3	3P28540_001_D1	14 63 67 114	R2R3
2P16200_001	5 54 58 105	R2R3	Mi_g029488	14 63 67 114	R2R3
2P17960_001	20 69 73 120	R2R3	Mi_g022136	14 63 67 114	R2R3
2P18090_001	16 65 69 116	R2R3	Mi_g029523	23 72 76 119	R2R3
2P19380_001	3 52 57 103	R2R3	Mi_g005621	132 180 185 231	R2R3
2P20600_001	14 63 67 114	R2R3	Mi_g024573	69 108 112 157	R2R3
2P22010_001	14 63 67 114	R2R3	Mi_g028191	14 63 67 114	R2R3
3P06470_001	14 63 67 114	R2R3	Mi_g003668	14 63 67 114	R2R3
3P07700_001	4 52 56 103	R2R3	Mi_g030419	14 63 67 114	R2R3
3P07940_001	14 63 67 114	R2R3	Mi_g005533	14 63 70 114	R2R3
3P07950_001	15 64 68 115	R2R3	Mi_g009976	23 72 76 123	R2R3
3P08370_001	23 72 76 119	R2R3	Mi_g024042	14 63 67 114	R2R3
3P09050_001	132 180 185 231	R2R3	Mi_g019328	14 63 67 114	R2R3
3P09450_001	14 63 67 114	R2R3	Mi_g031735	3 51 56 100	R2R3
3P09490_001	80 128 132 179	R2R3	4P19880_001_D1	14 63 74 114	R2R3
3P10060_001	14 63 70 114	R2R3	4P20150_001_D1	17 65 70 116	R2R3
3P12730_001	6 55 59 102	R2R3	4P25710_001_D1	17 66 70 122	R2R3
3P13070_001	21 69 74 120	R2R3	4P28090_001_D1	14 64 68 115	R2R3
3P16490_001	20 68 72 114	R2R3	4P28850_001_D1	16 65 69 116	R2R3
3P18970_001	18 66 70 117	R2R3	Mi_g020049	17 66 70 144	R2R3
3P19490_001	23 78 82 127	R2R3	Mi_g001029	20 68 73 119	R2R3
3P21000_001	77 126 130 177	R2R3	Mi_g007661	20 68 73 119	R2R3
3P21060_001	18 67 71 114	R2R3	Mi_g010968	23 70 75 121	R2R3

3P22200_001	14 63 67 114	R2R3	Mi_g008865	14 63 67 114	R2R3
3P24580_001	14 63 67 114	R2R3	Mi_g003265	14 63 67 114	R2R3
3P27700_001	14 63 67 114	R2R3	Mi_g030561	14 63 67 114	R2R3
3P28230_001	5 53 58 104	R2R3	Mi_g000657	14 63 67 114	R2R3
3P28540_001	14 63 67 114	R2R3	Mi_g004164	14 63 67 114	R2R3
4P00480_001	13 62 66 113	R2R3	Mi_g016970	14 63 67 114	R2R3
4P01050_001	14 64 71 115	R2R3	Mi_g009559	15 64 68 115	R2R3
4P05410_001	17 66 70 153	R2R3	Mi_g008508	14 63 71 114	R2R3
4P09450_001	20 68 73 119	R2R3	Mi_g008694	19 68 72 119	R2R3
4P11960_001	17 75 79 134	R2R3	Mi_g012524	19 68 72 119	R2R3
4P12950_001	6 53 58 104	R2R3	Mi_g012626	30 77 81 128	R2R3
4P18180_001	14 63 67 114	R2R3	Mi_g000080	3 52 56 103	R2R3
4P19030_001	14 63 67 114	R2R3	Mi_g001931	17 66 70 117	R2R3
4P19560_001	14 63 67 114	R2R3	Mi_g020623	14 64 71 115	R2R3
4P19880_001	14 63 71 114	R2R3	Mi_g018597	14 64 69 111	R2R3
4P19990_001	6 55 59 102	R2R3	5P12230_001_D1	14 63 67 114	R2R3
4P20150_001	17 65 70 116	R2R3	5P23110_001_D1	14 63 71 114	R2R3
4P21700_001	20 69 73 120	R2R3	5P23240_001_D1	14 63 67 114	R2R3
4P21910_001	5 54 58 105	R2R3	5P28940_001_D1	30 79 132 181	R2R3
4P23690_001	19 68 72 119	R2R3	Mi_g003787	14 64 71 115	R2R3
4P25260_001	14 64 69 115	R2R3	Mi_g029851	14 64 71 115	R2R3
4P25340_001	15 64 69 115	R2R3	Mi_g012470	14 64 71 115	R2R3
4P25710_001	17 66 70 122	R2R3	Mi_g025517	16 65 69 116	R2R3
4P27350_001	17 66 70 117	R2R3	Mi_g025554	14 63 67 114	R2R3
4P28090_001	14 64 68 115	R2R3	Mi_g008709	14 63 67 114	R2R3
4P28850_001	16 65 69 116	R2R3	Mi_g008708	14 63 67 114	R2R3

4P29230_001	18 67 71 118	R2R3	Mi_g008710	14 63 67 114	R2R3
4P29410_001	15 64 68 115	R2R3	Mi_g023340	14 63 67 114	R2R3
4P32240_001	14 64 71 115	R2R3	Mi_g030163	13 62 66 113	R2R3
5P01180_001	14 63 67 114	R2R3	Mi_g031658	20 69 74 120	R2R3
5P01930_001	14 64 71 115	R2R3	Mi_g031159	14 63 67 114	R2R3
5P03520_001	16 65 69 116	R2R3	Mi_g005086	10 59 64 110	R2R3
5P03920_001	14 63 67 114	R2R3	Mi_g015904	3 52 57 103	R2R3
5P05910_001	14 63 71 114	R2R3	Mi_g016607	32 79 135 184	R2R3
5P06530_001	35 84 88 135	R2R3	Mi_g012310	20 68 128 177	R2R3
5P07720_001	14 63 68 114	R2R3	Mi_g031711	5 51 102 151	R2R3
5P09280_001	14 64 69 115	R2R3	Mi_g005137	4 55 59 106	R2R3
5P12230_001	14 63 67 114	R2R3	6P09620_001_D1	13 62 66 113	R2R3
5P14600_001	13 62 66 113	R2R3	6P09760_001_D2	18 67 71 114	R2R3
5P18080_001	14 63 67 114	R2R3	6P09760_001_D4	7 56 60 103	R2R3
5P18600_001	14 63 67 114	R2R3	6P10700_001_D1	14 63 67 114	R2R3
5P21090_001	18 67 71 114	R2R3	6P15370_001_D1	4 53 57 104	R2R3
5P21250_001	14 63 67 114	R2R3	6P17630_001_D1	18 66 70 117	R2R3
5P21840_001	14 63 71 115	R2R3	6P19410_001_D2	20 56 83 132	R2R3
5P22760_001	14 64 71 115	R2R3	6P29310_001_D2	12 61 65 112	R2R3
5P23110_001	14 63 71 114	R2R3	6P31520_001_D1	16 65 69 116	R2R3
5P23240_001	14 63 67 114	R2R3	6P35800_001_D1	14 63 67 114	R2R3
5P23290_001	81 129 133 180	R2R3	Mi_g022049	14 63 67 114	R2R3
5P26000_001	14 63 67 114	R2R3	Mi_g021008	81 129 133 180	R2R3
5P27750_001	22 70 74 121	R2R3	Mi_g003034	14 63 67 114	R2R3
5P28350_001	10 59 64 110	R2R3	Mi_g010355	15 63 67 114	R2R3
5P28780_001	21 69 73 120	R2R3	Mi_g007720	86 125 129 174	R2R3

5P28940_001	30 79 132 181	R2R3	Mi_g014262	33 82 86 133	R2R3
5P29070_001	4 55 59 106	R2R3	Mi_g019039	31 77 81 128	R2R3
6P01850_001	14 64 71 115	R2R3	Mi_g016259	30 76 80 127	R2R3
6P02530_001	14 63 67 114	R2R3	Mi_g014574	20 68 72 119	R2R3
6P02550_001	14 63 67 114	R2R3	Mi_g027225	14 63 67 114	R2R3
6P02580_001	90 138 142 189	R2R3	Mi_g008329	14 63 67 114	R2R3
6P02690_001	14 63 67 114	R2R3	Mi_g006309	14 63 67 114	R2R3
6P04130_001	14 63 67 114	R2R3	Mi_g002791	14 64 71 115	R2R3
6P04400_001	14 63 67 114	R2R3	Mi_g021477	66 114 129 165	R2R3
6P06490_001	89 124 128 173	R2R3	Mi_g006425	78 126 141 177	R2R3
6P06800_001	35 84 88 135	R2R3	Mi_g026784	67 115 130 166	R2R3
6P07260_001	36 85 89 136	R2R3	Mi_g027397	42 90 94 141	R2R3
6P09620_001	13 62 66 113	R2R3	Mi_g009786	31 79 83 130	R2R3
6P09760_001	19 68 72 115	R2R3	Mi_g005585	68 116 120 167	R2R3
6P10650_001	31 77 81 128	R2R3	Mi_g024243	52 100 104 151	R2R3
6P10700_001	14 63 67 114	R2R3	Mi_g004323	114 162 169 213	R2R3
6P12930_001	14 63 67 114	R2R3	Mi_g008562	14 63 67 114	R2R3
6P14840_001	22 71 80 122	R2R3	Mi_g007306	14 63 67 114	R2R3
6P15370_001	4 53 57 104	R2R3	7P05570_001_D1	28 76 80 123	R2R3
6P15900_001	51 99 114 150	R2R3	7P21550_001_D5	41 90 94 123	R2R3
6P17630_001	18 66 70 117	R2R3	Mi_g006832	14 63 71 114	R2R3
6P25420_001	14 63 67 114	R2R3	Mi_g017465	4 52 56 103	R2R3
6P27290_001	24 73 77 120	R2R3	Mi_g004448	13 62 66 113	R2R3
6P29310_001	14 63 67 114	R2R3	Mi_g031010	8 57 61 108	R2R3
6P31520_001	16 65 69 116	R2R3	Mi_g013023	116 164 168 215	R2R3
6P31740_001	14 64 71 115	R2R3	Mi_g022053	90 138 142 189	R2R3

6P32250_001	66 114 129 165	R2R3	Mi_g013025	48 97 101 148	R2R3
6P32970_001	788 834 996 1033	R2R3	Mi_g013038	14 63 67 114	R2R3
6P33470_001	118 166 170 217	R2R3	Mi_g013039	14 63 67 114	R2R3
6P33620_001	61 109 125 174	R2R3	Mi_g012608	14 64 71 115	R2R3
6P33700_001	120 168 175 219	R2R3	Mi_g023093	14 64 71 115	R2R3
6P35800_001	14 63 67 114	R2R3	Mi_g010269	14 64 71 115	R2R3
6P36930_001	14 63 67 114	R2R3	Mi_g026997	14 64 68 115	R2R3
7P00280_001	2 38 42 89	R2R3	Mi_g005275	14 64 69 111	R2R3
7P05570_001	28 76 80 123	R2R3	Mi_g023833	14 63 67 114	R2R3
7P05680_001	14 63 71 114	R2R3	Mi_g023839	15 63 67 114	R2R3
7P08150_001	14 64 69 111	R2R3	Mi_g028992	14 64 71 115	R2R3
7P10550_001	4 55 59 106	R2R3	Mi_g015962	14 64 71 115	R2R3
7P11320_001	4 52 56 103	R2R3	Mi_g016662	14 64 71 115	R2R3
7P12520_001	14 63 71 114	R2R3	8P25220_001_D1	12 59 66 110	R2R3
7P13700_001	19 68 72 119	R2R3	Mi_g031709	128 176 183 227	R2R3
7P18080_001	14 63 67 114	R2R3	Mi_g031628	37 85 142 191	R2R3
7P18200_001	8 57 61 108	R2R3	Mi_g031627	22 68 112 161	R2R3
7P18390_001	116 164 168 215	R2R3	Mi_g017837	10 59 64 110	R2R3
7P18410_001	48 97 101 148	R2R3	Mi_g015447	10 59 63 110	R2R3
7P18550_001	14 63 67 114	R2R3	Mi_g019957	14 63 67 114	R2R3
7P18560_001	14 63 67 114	R2R3	Mi_g012710	14 63 67 114	R2R3
7P18680_001	14 63 71 125	R2R3	Mi_g028070	14 63 67 114	R2R3
7P19530_001	14 64 71 115	R2R3	Mi_g017600	14 64 69 115	R2R3
7P21010_001	14 63 67 114	R2R3	Mi_g009692	14 64 69 115	R2R3
7P21670_001	14 63 67 114	R2R3	Mi_g030276	14 64 69 115	R2R3
7P21740_001	16 64 68 115	R2R3	Mi_g032408	21 70 74 119	R2R3

7P25060_001	14 64 71 115	R2R3	Mi_g018396	18 67 71 118	R2R3
8P01400_001	14 63 67 114	R2R3	Mi_g015554	14 63 67 114	R2R3
8P02260_001	15 64 79 128	R2R3	Mi_g021391	14 63 67 114	R2R3
8P02290_001	129 177 184 228	R2R3	Mi_g004953	12 60 65 111	R2R3
8P02370_001	19 65 109 158	R2R3	Mi_g026899	54 104 111 157	R2R3
8P02380_001	38 85 142 191	R2R3	Mi_g028617	10 59 63 110	R2R3
8P02640_001	10 59 64 110	R2R3	9P03720_001_D2	5 53 57 104	R2R3
8P03510_001	13 62 66 113	R2R3	9P08480_001_D1	18 66 70 117	R2R3
8P10470_001	14 63 67 114	R2R3	9P08480_001_D2	20 68 72 116	R2R3
8P10800_001	23 72 76 119	R2R3	9P12180_001_D1	14 63 67 114	R2R3
8P11340_001	15 64 68 115	R2R3	9P22190_001_D1	14 64 68 115	R2R3
8P12710_001	17 66 70 117	R2R3	Mi_g026489	6 54 58 105	R2R3
8P14190_001	14 64 69 115	R2R3	Mi_g026546	798 844 1011 1049	R2R3
8P14540_001	21 70 74 119	R2R3	Mi_g021425	792 838 1000 1037	R2R3
8P16920_001	15 64 68 100	R2R3	Mi_g005961	69 118 122 165	R2R3
8P22690_001	14 63 67 114	R2R3	Mi_g001779	14 63 67 114	R2R3
8P24780_001	14 64 68 115	R2R3	Mi_g026681	14 63 67 114	R2R3
8P25220_001	12 59 66 110	R2R3	Mi_g008186	14 63 67 114	R2R3
8P25970_001	66 116 123 169	R2R3	9P08600_001_D4	14 63 67 114	R2R3
8P29910_001	23 72 76 119	R2R3	Mi_g001320	14 63 67 114	R2R3
8P31350_001	21 70 74 121	R2R3	Mi_g023815	14 63 67 114	R2R3
8P32480_001	14 63 67 114	R2R3	Mi_g023814	14 63 67 114	R2R3
8P33900_001	14 63 67 114	R2R3	Mi_g019901	14 63 67 114	R2R3
8P34340_001	10 59 63 110	R2R3	Mi_g007849	13 62 66 113	R2R3
9P03320_001	4 55 59 100	R2R3	Mi_g013221	13 62 66 113	R2R3
9P03720_001	6 54 58 105	R2R3	Mi_g022821	13 63 67 114	R2R3

9P04400_001	798 844 1011 1049	R2R3	Mi_g014953	4 52 56 98	R2R3
9P04990_001	47 95 99 146	R2R3	Mi_g029471	4 52 56 103	R2R3
9P07000_001	69 118 122 165	R2R3	Mi_g028730	14 63 67 114	R2R3
9P08480_001	18 66 70 117	R2R3	Mi_g005795	1 48 107 154	R2R3
9P08600_001	14 63 67 114	R2R3	Mi_g020867	7 55 59 103	R2R3
9P09790_001	14 64 71 115	R2R3	Mi_g019547	12 59 110 157	R2R3
9P11190_001	13 62 66 113	R2R3	Mi_g005856	4 55 59 106	R2R3
9P12180_001	14 63 67 114	R2R3	Mi_g001537	4 55 59 106	R2R3
9P13610_001	14 64 69 111	R2R3	Mi_g006673	4 52 56 103	R2R3
9P14850_001	14 63 67 114	R2R3	Mi_g005731	14 63 67 114	R2R3
9P15640_001	14 63 71 114	R2R3	Mi_g030664	14 64 69 115	R2R3
9P15750_001	4 52 56 98	R2R3	Mi_g006834	10 59 67 110	R2R3
9P16040_001	14 63 67 114	R2R3	Mi_g006538	2 51 55 102	R2R3
9P16640_001	23 72 76 119	R2R3	Mi_g022051	14 63 67 114	R2R3
9P17050_001	14 63 67 112	R2R3	Mi_g007343	11 60 64 111	R2R3
9P19380_001	4 55 59 106	R2R3	Mi_g024178	7 56 60 103	R2R3
9P20620_001	53 103 119 166	R2R3	Mi_g021826	7 56 60 103	R2R3
9P21870_001	15 82 137 184	R2R3	Mi_g024675	3 52 56 103	R2R3
9P22190_001	14 64 68 115	R2R3	Mi_g032081	3 52 56 103	R2R3
9P22600_001	5 53 58 104	R2R3	Mi_g002140	17 66 70 117	R2R3
9P23340_001	7 55 59 103	R2R3	Mi_g026115	14 63 67 114	R2R3
9P23760_001	12 59 110 157	R2R3	Mi_g003850	14 63 67 114	R2R3
9P24180_001	14 63 67 114	R2R3	Mi_g031193	14 63 67 114	R2R3
9P24560_001	14 63 67 114	R2R3	Mi_g008101	3 51 56 102	R2R3
9P25110_001	13 54 58 105	R2R3	Mi_g002676	14 63 67 114	R2R3

9P27380_001	15 63 67 114	R2R3	PDK_30s1024561g012_D4	14 63 67 114	R2R3
9P28330_001	4 55 59 106	R2R3	Mi_g010294	14 63 68 138	R2R3
9P28370_001	5 54 58 105	R2R3	Mi_g026448	4 55 59 100	R2R3
9P29090_001	4 52 56 103	R2R3	PDK_30s1185931g007_D1	14 63 67 111	R2R3
10P00660_001	36 84 108 157	R2R3	Mi_g018279	14 63 67 114	R2R3
Un_randomP00010_001	18 67 71 118	R2R3	Mi_g002872	11 59 116 165	R2R3
Un_randomP00040_001	14 63 67 114	R2R3	PDK_30s65509598g001_D2	15 64 68 115	R2R3
Un_randomP01280_001	26 74 79 125	R2R3	PDK_30s662981g002_D1	20 69 120 170	R2R3
Un_randomP02160_001	14 63 67 114	R2R3	PDK_30s704701g002_D5	14 74 78 125	R2R3
Un_randomP02890_001	14 63 67 114	R2R3	PDK_30s722171g012_D13	3 52 56 99	R2R3
Un_randomP07900_001	14 63 67 114	R2R3	Mi_g030747	14 63 67 114	R2R3
Un_randomP08600_001	18 67 71 114	R2R3	PDK_30s809821g005_D5	7 55 70 106	R2R3
Un_randomP11880_001	14 63 67 114	R2R3	Mi_g007512	14 63 67 114	R2R3
Un_randomP12700_001	14 63 67 114	R2R3	Mi_g014228	14 63 67 114	R2R3
Un_randomP15100_001	41 90 94 156	R2R3	PDK_30s844971g007_D4	14 63 67 114	R2R3
Un_randomP15560_001	15 64 68 115	R2R3	Mi_g000953	7 56 60 103	R2R3
Un_randomP16400_001	12 61 65 112	R2R3	Mi_g026602	4 55 59 106	R2R3
Un_randomP21080_001	22 71 75 122	R2R3	Mi_g031699	26 74 78 110	R2R3

Un_randomP27010_001	22 71 76 122	R2R3	0P02160_001_D1	14 63 67 114	R2R3
Un_randomP28780_001	14 63 67 114	R2R3	0P11880_001_D1	14 63 67 114	R2R3
			Mi_g020015	18 67 71 114	R2R3
			Mi_g015594	14 63 67 114	R2R3
			Mi_g026713	17 66 70 113	R2R3
			Mi_g032306	13 62 66 109	R2R3
			Mi_g002237	23 72 76 119	R2R3
			Mi_g028365	23 72 76 119	R2R3
			Mi_g018226	17 66 70 113	R2R3
			Mi_g029695	41 90 94 156	R2R3
			Mi_g031085	41 90 94 141	R2R3
			Mi_g026079	41 90 94 141	R2R3
			Mi_g003017	14 63 67 114	R2R3
			Mi_g002122	259 306 310 358 364 411 416 460	R4

[a] For clairity,the name of each gene were deleted by strings as "GSMUA_Achr" for *Musa acuminata*

[b].Consecutive pair of numbers denotes the start and stop position for each domain

[c] The gene names without header "Mi" were novel predicted gene by homology search against the MI genome assembly using trancription factors gene in other five speices and these genes were not in the final gene set.

Supplementary Table 22. Genome-wide duplication modes and ages MYB transcription gene family in *Musa acuminata*.

Duplicate1[a]	Location[b]	Duplicate1	Location	Ks	Ka	Ka/Ks	Mode
10P01620_001	ma10:3827267	10P01640_001	ma10:3884919	0.08	0.09	0.89	proximal
9P27380_001	ma9:31594796	9P27420_001	ma9:31608916	0.43	0.09	4.78	proximal
0P07900_001	ma0:32764662	1P23990_001	ma1:18428968	0.18	0.6	0.3	segmental
0P11880_001	ma0:56765463	6P06800_001	ma6:4618309	0.16	0.34	0.47	segmental
1P17730_001	ma1:13179687	10P06880_001	ma10:17161933	0.16	0.6	0.27	segmental
1P21020_001	ma1:15824207	10P19840_001	ma10:26059476	0.24	0.98	0.24	segmental
1P03450_001	ma1:2820599	11P02410_001	ma11:1676890	0.2	0.82	0.24	segmental
1P03470_001	ma1:2830179	11P02420_001	ma11:1680053	0.37	1.04	0.36	segmental
1P07550_001	ma1:5850917	11P07180_001	ma11:5611190	0.17	0.6	0.28	segmental
1P23990_001	ma1:18428968	11P07850_001	ma11:6130857	0.42	1.15	0.37	segmental
1P21020_001	ma1:15824207	11P13200_001	ma11:13238633	0.14	0.43	0.33	segmental
1P21020_001	ma1:15824207	2P22640_001	ma2:21653995	0.2	0.53	0.38	segmental
1P09750_001	ma1:7424574	3P13070_001	ma3:9594136	0.31	0.7	0.44	segmental
1P26140_001	ma1:21161795	3P13070_001	ma3:9594136	0.12	0.42	0.29	segmental
1P02420_001	ma1:1970933	4P25710_001	ma4:25131531	0.29	0.84	0.35	segmental
1P03140_001	ma1:2527188	4P25260_001	ma4:24842318	0.33	0.77	0.43	segmental
1P18610_001	ma1:13911762	4P19880_001	ma4:20707286	0.14	0.64	0.22	segmental
1P18990_001	ma1:14167062	4P19560_001	ma4:20495181	0.26	0.66	0.39	segmental

1P04910_001	ma1:4071302	4P01050_001	ma4:862510	0.34	0.76	0.45	segmental
1P04910_001	ma1:4071302	5P09280_001	ma5:6729584	0.34	0.73	0.47	segmental
1P03140_001	ma1:2527188	5P10690_001	ma5:7656005	0.25	0.49	0.51	segmental
1P18610_001	ma1:13911762	7P05680_001	ma7:4228294	0.14	0.61	0.23	segmental
1P12470_001	ma1:9547472	7P11320_001	ma7:9028627	1.24	1.6	0.78	segmental
1P26520_001	ma1:22105948	8P33900_001	ma8:34821383	0.11	0.34	0.32	segmental
1P24960_001	ma1:19356384	8P29910_001	ma8:32136422	0.24	0.73	0.33	segmental
1P07550_001	ma1:5850917	8P33900_001	ma8:34821383	0.17	0.68	0.25	segmental
1P09750_001	ma1:7424574	8P25220_001	ma8:29114006	0.29	0.57	0.51	segmental
10P19840_001	ma10:26059476	2P22640_001	ma2:21653995	0.2	0.63	0.32	segmental
10P16010_001	ma10:23714184	2P20600_001	ma2:20400441	0.1	0.44	0.23	segmental
10P25700_001	ma10:29594341	2P14770_001	ma2:16399343	0.26	0.63	0.41	segmental
10P26730_001	ma10:30151198	2P16200_001	ma2:17204463	0.12	0.4	0.3	segmental
10P14840_001	ma10:22957834	2P19880_001	ma2:19858005	0.46	0.23	2	segmental
10P19130_001	ma10:25624488	2P14770_001	ma2:16399343	0.35	1.24	0.28	segmental
10P01620_001	ma10:3827267	3P09450_001	ma3:6828783	0.27	0.78	0.35	segmental
10P24560_001	ma10:28968325	3P06470_001	ma3:4384442	0.22	0.88	0.25	segmental
10P19130_001	ma10:25624488	3P06470_001	ma3:4384442	0.41	1.36	0.3	segmental
10P19840_001	ma10:26059476	3P21060_001	ma3:22092435	0.23	0.48	0.48	segmental
10P19970_001	ma10:26139058	3P21000_001	ma3:22054692	0.35	0.52	0.67	segmental
10P00660_001	ma10:1222113	5P28940_001	ma5:28883215	0.15	0.61	0.25	segmental
10P05080_001	ma10:14706374	5P27750_001	ma5:28208071	0.24	0.46	0.52	segmental

10P01620_001	ma10:3827267	5P07720_001	ma5:5537475	0.42	0.81	0.52	segmental
10P26610_001	ma10:30090981	6P05030_001	ma6:3422319	0.23	0.48	0.48	segmental
10P29680_001	ma10:32111086	6P02550_001	ma6:1664084	0.38	0.69	0.55	segmental
10P24560_001	ma10:28968325	6P04130_001	ma6:2832832	0.16	0.53	0.3	segmental
10P12830_001	ma10:21711545	6P32250_001	ma6:31729692	0.26	0.56	0.46	segmental
10P10800_001	ma10:20485779	6P17630_001	ma6:11864241	0.36	1.12	0.32	segmental
10P12830_001	ma10:21711545	6P15900_001	ma6:10625059	0.14	0.4	0.35	segmental
10P13520_001	ma10:22118137	6P15370_001	ma6:10243841	0.11	0.49	0.22	segmental
10P14030_001	ma10:22432437	6P14840_001	ma6:9832272	0.16	0.36	0.44	segmental
10P16010_001	ma10:23714184	6P12930_001	ma6:8670597	0.18	0.46	0.39	segmental
10P18880_001	ma10:25439113	6P10650_001	ma6:7080327	0.14	0.47	0.3	segmental
10P19840_001	ma10:26059476	6P09760_001	ma6:6572824	0.24	0.49	0.49	segmental
10P19970_001	ma10:26139058	6P09620_001	ma6:6502243	0.33	0.68	0.49	segmental
10P00770_001	ma10:1381488	6P33700_001	ma6:32639515	0.38	0.73	0.52	segmental
10P29680_001	ma10:32111086	7P18410_001	ma7:21208138	0.21	0.49	0.43	segmental
10P29970_001	ma10:32275664	7P18680_001	ma7:21462395	0.18	0.44	0.41	segmental
10P14030_001	ma10:22432437	7P13700_001	ma7:11012737	0.49	0.89	0.55	segmental
10P29210_001	ma10:31806677	7P21670_001	ma7:24419242	0.28	0.54	0.52	segmental
10P29280_001	ma10:31868652	7P21550_001	ma7:24292621	0.25	0.12	2.08	segmental
10P03710_001	ma10:12019091	8P02640_001	ma8:1876800	0.18	0.61	0.3	segmental
10P00660_001	ma10:1222113	8P02380_001	ma8:1735034	0.2	0.64	0.31	segmental
10P00770_001	ma10:1381488	8P02290_001	ma8:1678965	0.38	0.57	0.67	segmental

10P26730_001	ma10:30151198	9P28370_001	ma9:32408981	0.15	0.63	0.24	segmental
10P24560_001	ma10:28968325	9P14850_001	ma9:9719265	0.25	0.53	0.47	segmental
10P12830_001	ma10:21711545	9P04990_001	ma9:3240268	0.24	0.57	0.42	segmental
10P01620_001	ma10:3827267	9P17050_001	ma9:11583434	0.27	0.77	0.35	segmental
11P02420_001	ma11:1680053	11P21360_001	ma11:22014665	0.21	0.66	0.32	segmental
11P16130_001	ma11:17834970	11P20830_001	ma11:21701206	0.29	0.5	0.58	segmental
11P04470_001	ma11:3350433	11P21020_001	ma11:21812151	0.34	2.63	0.13	segmental
11P04930_001	ma11:3696160	11P20830_001	ma11:21701206	0.24	0.58	0.41	segmental
11P07660_001	ma11:5940648	2P04200_001	ma2:9661223	0.17	0.43	0.4	segmental
11P17870_001	ma11:19406677	2P18090_001	ma2:18516616	0.3	0.96	0.31	segmental
11P23500_001	ma11:23380329	2P17960_001	ma2:18414859	0.24	0.83	0.29	segmental
11P17870_001	ma11:19406677	5P03520_001	ma5:2445891	0.39	1.35	0.29	segmental
11P03140_001	ma11:2260846	5P10440_001	ma5:7457562	0.11	0.72	0.15	segmental
11P24690_001	ma11:24116060	5P28350_001	ma5:28589027	0.22	0.6	0.37	segmental
11P26520_001	ma11:25333770	5P25730_001	ma5:26664437	0.23	0.79	0.29	segmental
11P20830_001	ma11:21701206	5P21090_001	ma5:23001338	0.17	0.31	0.55	segmental
11P21020_001	ma11:21812151	5P20940_001	ma5:22833763	0.32	1.59	0.2	segmental
11P22790_001	ma11:22957333	5P18600_001	ma5:20088661	1.51	2.34	0.65	segmental
11P23430_001	ma11:23320590	5P18080_001	ma5:19380625	0.16	0.41	0.39	segmental
11P02420_001	ma11:1680053	5P20480_001	ma5:22257736	0.17	0.56	0.3	segmental
11P25100_001	ma11:24340132	6P32250_001	ma6:31729692	0.25	0.36	0.69	segmental
11P10490_001	ma11:8300707	7P21740_001	ma7:24438808	0.95	0.31	3.06	segmental

11P01660_001	ma11:1124949	8P13660_001	ma8:10400849	0.11	0.34	0.32	segmental
11P20830_001	ma11:21701206	8P10800_001	ma8:7738326	0.31	0.5	0.62	segmental
11P16130_001	ma11:17834970	8P10800_001	ma8:7738326	0.23	0.52	0.44	segmental
11P02410_001	ma11:1676890	8P12710_001	ma8:9475867	0.1	0.42	0.24	segmental
11P03450_001	ma11:2490827	8P11800_001	ma8:8540654	0.08	0.26	0.31	segmental
11P04930_001	ma11:3696160	8P10800_001	ma8:7738326	0.2	0.61	0.33	segmental
11P23500_001	ma11:23380329	8P14540_001	ma8:11897078	0.35	1.07	0.33	segmental
2P22640_001	ma2:21653995	3P21060_001	ma3:22092435	0.22	0.61	0.36	segmental
2P22640_001	ma2:21653995	3P01370_001	ma3:977749	0.13	0.22	0.59	segmental
2P13930_001	ma2:15867063	4P23690_001	ma4:23700942	0.27	0.71	0.38	segmental
2P11450_001	ma2:14323124	4P29410_001	ma4:27377433	0.18	0.51	0.35	segmental
2P19380_001	ma2:19474985	5P06530_001	ma5:4800837	0.24	0.33	0.73	segmental
2P22010_001	ma2:21258919	6P10700_001	ma6:7121789	0.15	0.6	0.25	segmental
2P22640_001	ma2:21653995	6P09760_001	ma6:6572824	0.26	0.56	0.46	segmental
2P20600_001	ma2:20400441	6P12930_001	ma6:8670597	0.12	0.45	0.27	segmental
2P19380_001	ma2:19474985	7P13700_001	ma7:11012737	0.43	0.64	0.67	segmental
2P17960_001	ma2:18414859	8P14540_001	ma8:11897078	0.32	0.9	0.36	segmental
2P22640_001	ma2:21653995	8P18460_001	ma8:22616986	0.19	0.7	0.27	segmental
2P16200_001	ma2:17204463	9P28370_001	ma9:32408981	0.17	0.75	0.23	segmental
3P28540_001	ma3:27792238	4P06410_001	ma4:4867671	0.22	0.58	0.38	segmental
3P12200_001	ma3:9048575	4P19560_001	ma4:20495181	0.25	0.5	0.5	segmental
3P12730_001	ma3:9430861	4P19990_001	ma4:20777825	0.2	0.51	0.39	segmental

3P13070_001	ma3:9594136	4P20150_001	ma4:20875824	0.16	0.53	0.3	segmental
3P22200_001	ma3:23097441	5P26000_001	ma5:26945773	0.18	0.56	0.32	segmental
3P09450_001	ma3:6828783	5P07720_001	ma5:5537475	0.24	0.59	0.41	segmental
3P21000_001	ma3:22054692	6P09620_001	ma6:6502243	0.27	0.39	0.69	segmental
3P21060_001	ma3:22092435	6P09760_001	ma6:6572824	0.23	0.65	0.35	segmental
3P19490_001	ma3:20857458	6P06490_001	ma6:4374465	0.36	0.72	0.5	segmental
3P07940_001	ma3:5521762	8P22690_001	ma8:27268546	0.21	0.79	0.27	segmental
3P21060_001	ma3:22092435	8P18460_001	ma8:22616986	0.18	0.45	0.4	segmental
3P06470_001	ma3:4384442	9P14850_001	ma9:9719265	0.21	0.45	0.47	segmental
3P07940_001	ma3:5521762	9P16040_001	ma9:10751627	0.17	0.58	0.29	segmental
3P08370_001	ma3:5939949	9P16640_001	ma9:11293677	0.45	0.71	0.63	segmental
3P28230_001	ma3:27580987	9P22600_001	ma9:27709017	0.32	0.76	0.42	segmental
3P28540_001	ma3:27792238	9P22190_001	ma9:27319716	0.17	0.52	0.33	segmental
3P09450_001	ma3:6828783	9P17050_001	ma9:11583434	0.17	0.5	0.34	segmental
3P09490_001	ma3:6873436	9P17000_001	ma9:11543943	0.14	0.33	0.42	segmental
3P07700_001	ma3:5311517	9P15750_001	ma9:10441720	0.17	0.78	0.22	segmental
3P16490_001	ma3:17842513	9P08480_001	ma9:5431915	0.28	0.94	0.3	segmental
4P05410_001	ma4:4187540	4P11960_001	ma4:8728787	0.24	0.52	0.46	segmental
4P06410_001	ma4:4867671	4P13280_001	ma4:9890136	0.14	0.49	0.29	segmental
4P25340_001	ma4:24901615	4P29410_001	ma4:27377433	0.22	0.37	0.59	segmental
4P03800_001	ma4:3023347	4P06410_001	ma4:4867671	0.66	0.43	1.53	segmental
4P27350_001	ma4:26219201	5P03520_001	ma5:2445891	0.2	0.69	0.29	segmental

4P25260_001	ma4:24842318	5P10690_001	ma5:7656005	0.29	0.63	0.46	segmental
4P32240_001	ma4:29087072	5P01930_001	ma5:1183911	0.2	0.48	0.42	segmental
4P21910_001	ma4:22303702	5P03920_001	ma5:2760556	0.21	0.62	0.34	segmental
4P32240_001	ma4:29087072	6P31740_001	ma6:31431932	0.36	0.98	0.37	segmental
4P27730_001	ma4:26467154	7P00280_001	ma7:246110	0.38	0.61	0.62	segmental
4P23690_001	ma4:23700942	7P00280_001	ma7:246110	0.16	0.48	0.33	segmental
4P19880_001	ma4:20707286	7P05680_001	ma7:4228294	0.13	0.46	0.28	segmental
4P00480_001	ma4:365369	7P21010_001	ma7:23930895	0.17	0.52	0.33	segmental
4P09450_001	ma4:6860442	8P25220_001	ma8:29114006	0.23	0.47	0.49	segmental
4P13280_001	ma4:9890136	9P22190_001	ma9:27319716	0.16	0.55	0.29	segmental
5P22760_001	ma5:24681383	6P01850_001	ma6:1204556	0.15	0.38	0.39	segmental
5P23240_001	ma5:25024020	6P02550_001	ma6:1664084	0.21	0.52	0.4	segmental
5P23290_001	ma5:25055605	6P02580_001	ma6:1686852	0.27	0.53	0.51	segmental
5P27750_001	ma5:28208071	6P32250_001	ma6:31729692	0.18	0.48	0.38	segmental
5P28780_001	ma5:28794485	6P33470_001	ma6:32493021	0.14	0.17	0.82	segmental
5P21840_001	ma5:23977741	6P30830_001	ma6:30793406	0.2	0.39	0.51	segmental
5P28940_001	ma5:28883215	6P33620_001	ma6:32593456	0.22	0.98	0.22	segmental
5P17010_001	ma5:15785888	6P07260_001	ma6:4881251	0.21	0.45	0.47	segmental
5P01930_001	ma5:1183911	6P31740_001	ma6:31431932	0.34	0.77	0.44	segmental
5P27750_001	ma5:28208071	6P15900_001	ma6:10625059	0.3	0.89	0.34	segmental
5P06530_001	ma5:4800837	7P13700_001	ma7:11012737	0.48	0.57	0.84	segmental
5P23110_001	ma5:24947461	7P18680_001	ma7:21462395	0.17	0.47	0.36	segmental

5P23240_001	ma5:25024020	7P18410_001	ma7:21208138	0.11	0.63	0.17	segmental
5P23290_001	ma5:25055605	7P18390_001	ma7:21181466	0.37	0.63	0.59	segmental
5P22760_001	ma5:24681383	7P19530_001	ma7:22316984	0.19	0.6	0.32	segmental
5P28940_001	ma5:28883215	8P02370_001	ma8:1731834	0.2	0.52	0.38	segmental
5P22760_001	ma5:24681383	8P24780_001	ma8:28831601	0.22	0.49	0.45	segmental
5P21090_001	ma5:23001338	8P10800_001	ma8:7738326	0.14	0.4	0.35	segmental
5P21250_001	ma5:23271254	8P10470_001	ma8:7453357	0.19	0.73	0.26	segmental
5P05910_001	ma5:4409346	9P15640_001	ma9:10341287	0.2	0.72	0.28	segmental
5P07720_001	ma5:5537475	9P17050_001	ma9:11583434	0.2	0.47	0.43	segmental
5P01930_001	ma5:1183911	9P09790_001	ma9:6315751	0.31	0.73	0.42	segmental
5P27750_001	ma5:28208071	9P04990_001	ma9:3240268	0.22	0.52	0.42	segmental
5P28780_001	ma5:28794485	9P03720_001	ma9:2564190	0.8	1.29	0.62	segmental
5P29070_001	ma5:28955095	9P03320_001	ma9:2317111	0.26	0.29	0.9	segmental
6P01850_001	ma6:1204556	7P19530_001	ma7:22316984	0.17	0.56	0.3	segmental
6P02530_001	ma6:1646480	7P18550_001	ma7:21322148	0.29	0.5	0.58	segmental
6P02550_001	ma6:1664084	7P18410_001	ma7:21208138	0.27	0.59	0.46	segmental
6P02580_001	ma6:1686852	7P18390_001	ma7:21181466	0.36	0.8	0.45	segmental
6P02690_001	ma6:1746525	7P18080_001	ma7:20896774	0.24	0.66	0.36	segmental
6P05030_001	ma6:3422319	7P10520_001	ma7:8469177	0.33	0.18	1.83	segmental
6P14840_001	ma6:9832272	7P13700_001	ma7:11012737	0.52	0.97	0.54	segmental
6P33620_001	ma6:32593456	8P02260_001	ma8:1656100	0.35	0.63	0.56	segmental
6P33700_001	ma6:32639515	8P02290_001	ma8:1678965	0.37	0.71	0.52	segmental

6P27290_001	ma6:27836929	9P07000_001	ma9:4396755	NA	NA	NA	segmental
6P25420_001	ma6:26510462	9P08600_001	ma9:5530063	0.13	0.39	0.33	segmental
6P32250_001	ma6:31729692	9P04990_001	ma9:3240268	0.17	0.42	0.4	segmental
6P32970_001	ma6:32156680	9P04400_001	ma9:2930622	0.18	0.49	0.37	segmental
6P33470_001	ma6:32493021	9P03720_001	ma9:2564190	1.53	1.73	0.88	segmental
6P31410_001	ma6:31212076	9P10150_001	ma9:6524734	0.15	0.36	0.42	segmental
6P31740_001	ma6:31431932	9P09790_001	ma9:6315751	0.23	0.47	0.49	segmental
6P29310_001	ma6:29578336	9P12180_001	ma9:7955715	0.21	0.63	0.33	segmental
6P32250_001	ma6:31729692	9P17000_001	ma9:11543943	0.26	0.57	0.46	segmental
7P19530_001	ma7:22316984	8P24780_001	ma8:28831601	0.23	0.68	0.34	segmental
7P10550_001	ma7:8483769	9P28330_001	ma9:32378337	0.15	0.63	0.24	segmental
7P18680_001	ma7:21462395	9P15640_001	ma9:10341287	0.23	0.81	0.28	segmental
8P03510_001	ma8:2402421	9P11190_001	ma9:7282803	0.15	0.49	0.31	segmental
8P22690_001	ma8:27268546	9P16040_001	ma9:10751627	0.23	0.78	0.29	segmental
8P03510_001	ma8:2402421	9P25100_001	ma9:29693387	0.79	0.34	2.32	segmental
9P12180_001	ma9:7955715	9P24560_001	ma9:29318237	0.35	0.57	0.61	segmental
11P09170_001	ma11:7079110	11P09180_001	ma11:7085790	1.52	0.25	6.08	tandem
3P07940_001	ma3:5521762	3P07950_001	ma3:5529381	0.44	1.75	0.25	tandem
7P18550_001	ma7:21322148	7P18560_001	ma7:21326915	0.37	1.09	0.34	tandem
8P02370_001	ma8:1731834	8P02380_001	ma8:1735034	0.17	0.28	0.61	tandem
9P25100_001	ma9:29693387	9P25110_001	ma9:29699940	0.7	0.23	3.04	tandem
0P00010_001	ma0:5911	8P29910_001	ma8:32136422	0.49	0.92	0.53	transposed

0P01280_001	ma0:4240224	9P22600_001	ma9:27709017	0.37	0.89	0.42	transposed
0P02160_001	ma0:10079858	7P21010_001	ma7:23930895	0.45	1.51	0.3	transposed
0P02890_001	ma0:14335878	11P23430_001	ma11:23320590	0.49	1.2	0.41	transposed
0P04880_001	ma0:19257049	8P02260_001	ma8:1656100	0.56	1.51	0.37	transposed
0P05930_001	ma0:25461097	7P21550_001	ma7:24292621	0.46	0.4	1.15	transposed
0P08600_001	ma0:39288889	8P10800_001	ma8:7738326	0.22	0.56	0.39	transposed
0P10790_001	ma0:52550469	11P07660_001	ma11:5940648	0.23	0.46	0.5	transposed
0P12700_001	ma0:60970282	9P27380_001	ma9:31594796	0.63	1.71	0.37	transposed
0P15100_001	ma0:71214440	10P29280_001	ma10:31868652	0.33	0.5	0.66	transposed
0P15560_001	ma0:73673346	3P06470_001	ma3:4384442	0.27	1.03	0.26	transposed
0P16400_001	ma0:77590851	3P21000_001	ma3:22054692	0.18	0.54	0.33	transposed
0P16400_001	ma0:77590851	9P24180_001	ma9:28998002	0.31	1.04	0.3	transposed
0P18640_001	ma0:89403099	7P10520_001	ma7:8469177	1.57	1.41	1.11	transposed
0P19120_001	ma0:91767882	2P04730_001	ma2:10035776	0.59	0.37	1.59	transposed
0P21080_001	ma0:102480920	6P35800_001	ma6:33924613	0.69	1.97	0.35	transposed
0P21080_001	ma0:102480920	8P31350_001	ma8:33047459	0.21	0.82	0.26	transposed
0P21150_001	ma0:103035185	5P07380_001	ma5:5309335	0.44	0.63	0.7	transposed
0P21150_001	ma0:103035185	9P22600_001	ma9:27709017	0.64	0.84	0.76	transposed
0P22350_001	ma0:109329376	9P22190_001	ma9:27319716	0.26	0.62	0.42	transposed
0P27010_001	ma0:132135986	8P29910_001	ma8:32136422	0.26	0.82	0.32	transposed
0P28780_001	ma0:139549853	6P07260_001	ma6:4881251	0.09	0.43	0.21	transposed
10P04250_001	ma10:13791652	11P26520_001	ma11:25333770	0.22	0.78	0.28	transposed

10P04590_001	ma10:14224803	2P20600_001	ma2:20400441	0.28	0.58	0.48	transposed
10P08070_001	ma10:18092917	9P11190_001	ma9:7282803	0.73	1.41	0.52	transposed
10P08350_001	ma10:18359986	5P01930_001	ma5:1183911	0.32	0.78	0.41	transposed
10P17610_001	ma10:24759638	3P07940_001	ma3:5521762	0.6	1.57	0.38	transposed
11P00410_001	ma11:238132	11P22820_001	ma11:22972764	0.37	0.82	0.45	transposed
11P04090_001	ma11:2997742	6P04130_001	ma6:2832832	0.7	1.39	0.5	transposed
11P07550_001	ma11:5887956	11P04080_001	ma11:2992016	0.41	1.14	0.36	transposed
11P09110_001	ma11:7039899	6P19410_001	ma6:13697491	0.14	0.55	0.25	transposed
11P09160_001	ma11:7075580	5P27750_001	ma5:28208071	0.54	1.49	0.36	transposed
11P13080_001	ma11:12858603	7P21010_001	ma7:23930895	0.51	1.28	0.4	transposed
11P13790_001	ma11:14824831	7P21740_001	ma7:24438808	1.3	1.2	1.08	transposed
11P15910_001	ma11:17592155	11P00430_001	ma11:259223	0.33	0.55	0.6	transposed
11P17150_001	ma11:18825848	1P06510_001	ma1:5193504	0.35	0.8	0.44	transposed
11P17190_001	ma11:18859717	8P12710_001	ma8:9475867	0.19	0.71	0.27	transposed
11P17730_001	ma11:19301622	3P12610_001	ma3:9339168	0.2	1.02	0.2	transposed
11P26140_001	ma11:25024790	10P28340_001	ma10:31192481	0.63	2.92	0.22	transposed
1P09180_001	ma1:7027537	5P03920_001	ma5:2760556	0.41	1.73	0.24	transposed
1P17150_001	ma1:12797871	10P29280_001	ma10:31868652	0.23	0.46	0.5	transposed
1P17150_001	ma1:12797871	7P02510_001	ma7:1980771	0.47	0.66	0.71	transposed
1P17480_001	ma1:13006455	4P11960_001	ma4:8728787	0.4	0.88	0.45	transposed
1P22380_001	ma1:16850209	10P29970_001	ma10:32275664	0.23	0.83	0.28	transposed
1P27270_001	ma1:23895634	8P29910_001	ma8:32136422	0.49	1.21	0.4	transposed

2P01830_001	ma2:7701667	1P18990_001	ma1:14167062	0.32	0.81	0.4	transposed
2P02970_001	ma2:8708443	3P06470_001	ma3:4384442	0.57	1.53	0.37	transposed
2P03880_001	ma2:9453094	7P10520_001	ma7:8469177	NA	NA	NA	transposed
2P04730_001	ma2:10035776	11P23430_001	ma11:23320590	0.47	1.49	0.32	transposed
2P07780_001	ma2:12087173	1P25120_001	ma1:19553840	0.76	1.45	0.52	transposed
2P19890_001	ma2:19864656	10P26610_001	ma10:30090981	0.81	0.81	1	transposed
3P09050_001	ma3:6536827	3P19490_001	ma3:20857458	0.4	0.88	0.45	transposed
3P10060_001	ma3:7278997	6P29310_001	ma6:29578336	0.79	2.65	0.3	transposed
3P13920_001	ma3:13050078	8P02380_001	ma8:1735034	0.92	2.34	0.39	transposed
3P18970_001	ma3:20354812	9P08480_001	ma9:5431915	0.36	1.18	0.31	transposed
3P24580_001	ma3:25066979	1P18990_001	ma1:14167062	0.3	0.97	0.31	transposed
3P27700_001	ma3:27206568	8P22690_001	ma8:27268546	NA	NA	NA	transposed
4P06340_001	ma4:4796268	11P02420_001	ma11:1680053	NA	NA	NA	transposed
4P16900_001	ma4:15506891	11P23990_001	ma11:23683457	1.23	0.59	2.08	transposed
4P18180_001	ma4:18816135	5P18600_001	ma5:20088661	0.2	0.45	0.44	transposed
4P19030_001	ma4:20118750	11P23430_001	ma11:23320590	0.32	1.23	0.26	transposed
4P21700_001	ma4:22113988	11P23500_001	ma11:23380329	0.42	1.56	0.27	transposed
4P24510_001	ma4:24280662	9P07000_001	ma9:4396755	1.41	0.5	2.82	transposed
4P28090_001	ma4:26633991	5P01930_001	ma5:1183911	0.2	0.5	0.4	transposed
4P28850_001	ma4:27084708	5P03520_001	ma5:2445891	0.4	1.15	0.35	transposed
4P29230_001	ma4:27282657	8P12710_001	ma8:9475867	0.24	0.64	0.38	transposed
5P01180_001	ma5:678774	6P07260_001	ma6:4881251	0.29	0.61	0.48	transposed

5P07380_001	ma5:5309335	6P33470_001	ma6:32493021	1.94	1.62	1.2	transposed
5P14600_001	ma5:10539751	5P03920_001	ma5:2760556	0.24	0.63	0.38	transposed
5P15730_001	ma5:11951295	7P19530_001	ma7:22316984	1.36	0.51	2.67	transposed
5P22410_001	ma5:24497053	10P29680_001	ma10:32111086	0.46	0.41	1.12	transposed
6P04400_001	ma6:3004586	9P27380_001	ma9:31594796	0.23	0.84	0.27	transposed
6P31520_001	ma6:31267214	5P03520_001	ma5:2445891	0.45	1.4	0.32	transposed
7P02510_001	ma7:1980771	10P29280_001	ma10:31868652	0.52	0.53	0.98	transposed
7P05570_001	ma7:4142609	8P10800_001	ma8:7738326	0.41	1.23	0.33	transposed
7P08150_001	ma7:6094819	5P22760_001	ma5:24681383	0.5	2.85	0.18	transposed
7P18200_001	ma7:20997754	10P26730_001	ma10:30151198	0.34	0.95	0.36	transposed
7P21560_001	ma7:24305018	10P29280_001	ma10:31868652	0.25	0.22	1.14	transposed
7P25060_001	ma7:26860985	4P01050_001	ma4:862510	0.4	0.92	0.43	transposed
8P01400_001	ma8:1175893	7P21010_001	ma7:23930895	0.39	1.14	0.34	transposed
8P01930_001	ma8:1438932	9P22600_001	ma9:27709017	0.61	0.91	0.67	transposed
8P05290_001	ma8:3439354	11P04910_001	ma11:3686976	NA	NA	NA	transposed
8P11810_001	ma8:8552080	11P03450_001	ma11:2490827	0.3	0.29	1.03	transposed
8P16920_001	ma8:14980756	2P22010_001	ma2:21258919	0.17	0.18	0.94	transposed
8P25970_001	ma8:29688201	4P01050_001	ma4:862510	0.61	0.59	1.03	transposed
8P31350_001	ma8:33047459	1P25120_001	ma1:19553840	0.72	1.33	0.54	transposed
8P34340_001	ma8:35236263	10P03710_001	ma10:12019091	0.33	0.96	0.34	transposed
9P10160_001	ma9:6535135	6P31410_001	ma6:31212076	1.82	0.9	2.02	transposed
9P19380_001	ma9:20003709	9P28330_001	ma9:32378337	0.46	1.24	0.37	transposed

9P20620_001	ma9:24755344	1P17730_001	ma1:13179687	0.15	0.66	0.23	transposed
9P21870_001	ma9:27003360	1P17730_001	ma1:13179687	0.39	1.95	0.2	transposed
9P23340_001	ma9:28198983	2P03880_001	ma2:9453094	NA	NA	NA	transposed
9P23340_001	ma9:28198983	7P10520_001	ma7:8469177	1.57	1.79	0.88	transposed
9P23760_001	ma9:28602350	1P17730_001	ma1:13179687	0.16	0.86	0.19	transposed
9P24180_001	ma9:28998002	1P26520_001	ma1:22105948	0.14	0.56	0.25	transposed
9P28160_001	ma9:32239521	11P01660_001	ma11:1124949	1.25	2.27	0.55	transposed
9P29090_001	ma9:32857364	7P11320_001	ma7:9028627	NA	NA	NA	transposed

[a] The name of *Musa acuminata* duplicated genes were abbreviated and indicated by letter ‘P’, and corresponding chromosomes also abbreviated as ‘ma’;

[b] The location referred to the first site of alignment on the chromosomes of *Musa acuminata* respectively

Supplementary Table 23 Genome-wide duplication modes and ages of MYB transcription gene family in *Musa itinerans*.

Duplicate1	Location[a]	Duplicate1	Location	Ks	Ka	Ka/Ks	Mode
Mi_g026023	mi8:23959678	Mi_g022486	mi8:23997594	0.16	0.65	0.24	proximal
Mi_g006834	mi7:4531089	Mi_g006832	mi7:4539660	0	0	0	proximal
Mi_g019853	mi1:2923201	Mi_g020932	mi11:2174609	0.19	0.79	0.24	segmental
Mi_g009692	mi1:2615701	Mi_g030276	mi4:21624024	0.21	0.75	0.28	segmental
Mi_g019853	mi1:2923201	Mi_g029097	mi4:24185146	0.21	0.46	0.46	segmental
Mi_g015962	mi1:4172748	Mi_g009254	mi4:1006715	0.31	0.74	0.41	segmental
Mi_g008101	mi1:8040677	Mi_g010968	mi4:10844301	0.32	0.73	0.44	segmental
Mi_g015962	mi1:4172748	Mi_g030664	mi5:6933931	0.09	0.19	0.45	segmental
Mi_g009692	mi1:2615701	Mi_g000872	mi5:7972037	0.21	0.46	0.45	segmental
Mi_g019853	mi1:2923201	Mi_g002140	mi8:10088354	0.19	0.55	0.35	segmental
Mi_g011352	mi10:11976710	Mi_g008186	mi2:14623575	0.15	0.57	0.27	segmental
Mi_g012174	mi10:18347951	Mi_g009075	mi2:10204791	1.07	1.52	0.7	segmental
Mi_g016283	mi10:13989619	Mi_g009075	mi2:10204791	1.15	1	1.14	segmental
Mi_g008837	mi10:17573538	Mi_g011643	mi3:4741460	0.16	0.6	0.26	segmental
Mi_g016283	mi10:13989619	Mi_g011643	mi3:4741460	0.34	1.16	0.29	segmental
Mi_g026784	mi10:9935168	Mi_g006425	mi6:11620634	0.12	0.39	0.3	segmental
Mi_g015857	mi10:10658394	Mi_g007998	mi6:10616573	0.16	0.32	0.49	segmental
Mi_g016259	mi10:13791068	Mi_g019039	mi6:7845398	0.14	0.44	0.32	segmental

Mi_g017415	mi10:934502	Mi_g004323	mi6:27312708	0.31	0.71	0.44	segmental
Mi_g026115	mi10:20945633	Mi_g013025	mi7:15326570	0.09	0.28	0.32	segmental
Mi_g026137	mi10:21140177	Mi_g013052	mi7:15554307	0.17	0.45	0.38	segmental
Mi_g015857	mi10:10658394	Mi_g007343	mi7:11525363	0.06	0.23	0.25	segmental
Mi_g029930	mi10:18849928	Mi_g001538	mi7:8628712	0.18	0.22	0.82	segmental
Mi_g016052	mi10:20664649	Mi_g023833	mi7:17931842	0.34	0.73	0.47	segmental
Mi_g026079	mi10:20693875	Mi_g007855	mi7:17851925	0.36	0.13	2.67	segmental
Mi_g016607	mi10:859170	Mi_g031627	mi8:2010146	0.23	0.36	0.64	segmental
Mi_g017415	mi10:934502	Mi_g031709	mi8:1935643	0.39	0.68	0.57	segmental
Mi_g008837	mi10:17573538	Mi_g031193	mi9:10525472	0.1	0.65	0.15	segmental
Mi_g016283	mi10:13989619	Mi_g031193	mi9:10525472	0.19	0.77	0.24	segmental
Mi_g026137	mi10:21140177	Mi_g010260	mi9:11221247	0.2	0.72	0.28	segmental
Mi_g024178	mi11:13903673	Mi_g012944	mi11:17692660	0.22	0.46	0.48	segmental
Mi_g026713	mi11:4118960	Mi_g012944	mi11:17692660	0.24	0.58	0.41	segmental
Mi_g000673	mi11:6383105	Mi_g008629	mi2:3308069	0.05	0.16	0.31	segmental
Mi_g013106	mi11:15337792	Mi_g011505	mi2:12374106	0.26	0.58	0.45	segmental
Mi_g018396	mi11:19557350	Mi_g011516	mi2:12263600	0.09	0.38	0.23	segmental
Mi_g018353	mi11:7768542	Mi_g006597	mi2:15307155	0.12	0.49	0.25	segmental
Mi_g012944	mi11:17692660	Mi_g021826	mi4:7544613	0.07	0.26	0.28	segmental
Mi_g013106	mi11:15337792	Mi_g025517	mi5:1951773	0.38	1.42	0.27	segmental
Mi_g016435	mi11:20396468	Mi_g005086	mi5:24027298	0.29	0.64	0.45	segmental

Mi_g003265	mi11:19469450	Mi_g004164	mi5:14940268	0.11	0.29	0.38	segmental
Mi_g017949	mi11:8782261	Mi_g023839	mi7:17954246	1.65	0.31	5.26	segmental
Mi_g012944	mi11:17692660	Mi_g028365	mi8:8188380	0.29	0.52	0.55	segmental
Mi_g008382	mi11:1209382	Mi_g000576	mi8:10982834	0.11	0.36	0.3	segmental
Mi_g024178	mi11:13903673	Mi_g028365	mi8:8188380	0.19	0.48	0.39	segmental
Mi_g020932	mi11:2174609	Mi_g002140	mi8:10088354	0.11	0.26	0.42	segmental
Mi_g018396	mi11:19557350	Mi_g032408	mi8:11654421	0.11	0.41	0.28	segmental
Mi_g006597	mi2:15307155	Mi_g021090	mi3:1430597	0.09	0.44	0.19	segmental
Mi_g013325	mi2:9627690	Mi_g012524	mi4:20374634	0.28	0.63	0.44	segmental
Mi_g023573	mi2:7978043	Mi_g018991	mi4:24296918	0.21	0.59	0.35	segmental
Mi_g005722	mi2:15925203	Mi_g015986	mi6:6906503	0.23	0.45	0.52	segmental
Mi_g013325	mi2:9627690	Mi_g012626	mi7:224100	0.35	0.6	0.59	segmental
Mi_g011516	mi2:12263600	Mi_g032408	mi8:11654421	0.3	0.74	0.41	segmental
Mi_g023815	mi3:18164105	Mi_g026681	mi5:22272500	0.14	0.43	0.33	segmental
Mi_g005585	mi3:7372820	Mi_g024243	mi5:23651737	0.27	0.7	0.38	segmental
Mi_g024573	mi3:15984915	Mi_g007720	mi6:4621333	2.24	1.32	1.7	segmental
Mi_g029488	mi3:5903872	Mi_g013038	mi7:15430410	0.45	1.48	0.3	segmental
Mi_g029488	mi3:5903872	Mi_g015554	mi8:17772344	0.21	0.71	0.29	segmental
Mi_g011643	mi3:4741460	Mi_g031193	mi9:10525472	0.11	0.23	0.49	segmental
Mi_g005585	mi3:7372820	Mi_g012547	mi9:12095183	0.12	0.2	0.62	segmental
Mi_g005585	mi3:7372820	Mi_g009786	mi9:3461480	0.25	0.7	0.36	segmental

Mi_g029471	mi3:5703873	Mi_g014953	mi9:11347997	0.17	0.67	0.25	segmental
Mi_g001931	mi4:23032506	Mi_g025517	mi5:1951773	0.26	0.6	0.44	segmental
Mi_g030276	mi4:21624024	Mi_g000872	mi5:7972037	0.2	0.59	0.33	segmental
Mi_g020623	mi4:25952754	Mi_g003787	mi5:1143055	0.21	0.46	0.46	segmental
Mi_g008708	mi4:18781489	Mi_g025554	mi5:2349713	0.15	0.51	0.3	segmental
Mi_g020623	mi4:25952754	Mi_g002791	mi6:25555493	0.36	0.78	0.47	segmental
Mi_g012524	mi4:20374634	Mi_g012626	mi7:224100	0.17	0.51	0.33	segmental
Mi_g021826	mi4:7544613	Mi_g028365	mi8:8188380	0.1	0.32	0.32	segmental
Mi_g010968	mi4:10844301	Mi_g007661	mi9:16429117	0.41	0.77	0.53	segmental
Mi_g010269	mi5:19846589	Mi_g023093	mi6:1317584	0.14	0.38	0.37	segmental
Mi_g021008	mi5:20252585	Mi_g022053	mi6:1758744	0.24	0.51	0.47	segmental
Mi_g024243	mi5:23651737	Mi_g021477	mi6:25855352	0.21	0.56	0.36	segmental
Mi_g003787	mi5:1143055	Mi_g002791	mi6:25555493	0.37	0.72	0.52	segmental
Mi_g024243	mi5:23651737	Mi_g006425	mi6:11620634	0.23	0.64	0.37	segmental
Mi_g021008	mi5:20252585	Mi_g013023	mi7:15304512	0.34	0.56	0.61	segmental
Mi_g025663	mi5:5810722	Mi_g023833	mi7:17931842	0.36	1.15	0.32	segmental
Mi_g025663	mi5:5810722	Mi_g012551	mi9:12178451	0.19	0.55	0.35	segmental
Mi_g026681	mi5:22272500	Mi_g001779	mi9:5988156	0.28	0.79	0.36	segmental
Mi_g005112	mi5:24240498	Mi_g026489	mi9:2776800	0.66	1.04	0.64	segmental
Mi_g005137	mi5:24410333	Mi_g026448	mi9:2484854	0.11	0.3	0.36	segmental
Mi_g015986	mi6:6906503	Mi_g009970	mi6:7197336	0.16	0.49	0.33	segmental

Mi_g006425	mi6:11620634	Mi_g021477	mi6:25855352	0.24	0.61	0.4	segmental
Mi_g022049	mi6:1712968	Mi_g013038	mi7:15430410	0.22	0.37	0.61	segmental
Mi_g022051	mi6:1731674	Mi_g013025	mi7:15326570	0.04	0.28	0.15	segmental
Mi_g022053	mi6:1758744	Mi_g013023	mi7:15304512	0.2	0.45	0.45	segmental
Mi_g007998	mi6:10616573	Mi_g007343	mi7:11525363	0.07	0.22	0.3	segmental
Mi_g004323	mi6:27312708	Mi_g031709	mi8:1935643	0.36	0.81	0.45	segmental
Mi_g020057	mi6:21840169	Mi_g005961	mi9:4696768	NA	NA	NA	segmental
Mi_g027225	mi6:23625086	Mi_g008329	mi9:18061682	0.14	0.54	0.26	segmental
Mi_g019901	mi6:20440835	Mi_g001779	mi9:5988156	0.15	0.39	0.37	segmental
Mi_g021477	mi6:25855352	Mi_g009786	mi9:3461480	0.15	0.39	0.39	segmental
Mi_g021425	mi6:26333171	Mi_g026546	mi9:3120282	0.18	0.51	0.34	segmental
Mi_g020828	mi6:25343407	Mi_g002122	mi9:6941395	0.11	0.33	0.32	segmental
Mi_g002791	mi6:25555493	Mi_g012470	mi9:6747271	0.22	0.38	0.6	segmental
Mi_g007849	mi9:7701948	Mi_g022821	mi9:18407935	0.17	0.57	0.29	segmental
Mi_g022486	mi8:23997594	Mi_g022485	mi8:24004883	0	0.02	0.14	tandem
Mi_g008709	mi4:18784060	Mi_g008710	mi4:18786352	0	0	NA	tandem
Mi_g008708	mi4:18781489	Mi_g008709	mi4:18784060	0	0	NA	tandem
Mi_g013038	mi7:15430410	Mi_g013039	mi7:15434725	0.33	0.98	0.33	tandem
Mi_g031627	mi8:2010146	Mi_g031628	mi8:2013899	0.17	0.29	0.6	tandem
Mi_g023815	mi3:18164105	Mi_g023814	mi3:18170762	0	0	0	tandem
Mi_g015594	mi0:122808	Mi_g024178	mi11:13903673	0.09	0.43	0.21	transposed

Mi_g012341	mi6:27047886	Mi_g001538	mi7:8628712	1.67	1.51	1.1	transposed
Mi_g000908	mi10:255366	Mi_g031711	mi8:1909382	0.36	0.88	0.4	transposed
Mi_g017225	mi10:13102386	Mi_g029488	mi3:5903872	0.57	1.7	0.33	transposed
Mi_g017225	mi10:13102386	Mi_g010355	mi9:20391316	0.41	1.18	0.34	transposed
Mi_g007232	mi11:7556780	Mi_g022053	mi6:1758744	0.37	0.92	0.41	transposed
Mi_g005370	mi11:11370632	Mi_g012944	mi11:17692660	0.6	1.91	0.31	transposed
Mi_g005338	mi11:11785350	Mi_g023839	mi7:17954246	NA	NA	NA	transposed
Mi_g031532	mi11:14664361	Mi_g019853	mi1:2923201	0.3	0.81	0.38	transposed
Mi_g010941	mi9:17747983	Mi_g000967	mi3:9857540	0.16	1.13	0.15	transposed
Mi_g010316	mi1:10486724	Mi_g017465	mi7:9258956	1.18	1.39	0.85	transposed
Mi_g010316	mi1:10486724	Mi_g006597	mi2:15307155	1.28	2.05	0.62	transposed
Mi_g022835	mi1:22663937	Mi_g002237	mi8:22931823	0.48	1.24	0.39	transposed
Mi_g008651	mi2:3072072	Mi_g029930	mi10:18849928	0.4	0.59	0.67	transposed
Mi_g028414	mi8:8730115	Mi_g020049	mi4:10061016	0.41	2.09	0.2	transposed
Mi_g031056	mi1:14148975	Mi_g023573	mi2:7978043	0.23	0.5	0.47	transposed
Mi_g029523	mi3:6353211	Mi_g002237	mi8:22931823	0.51	0.97	0.52	transposed
Mi_g005621	mi3:7020580	Mi_g007720	mi6:4621333	2.18	1.38	1.59	transposed
Mi_g005533	mi3:7778217	Mi_g011643	mi3:4741460	0.9	1.63	0.55	transposed
Mi_g005645	mi3:6819975	Mi_g007720	mi6:4621333	NA	NA	NA	transposed
Mi_g009976	mi6:7058708	Mi_g019901	mi6:20440835	0.44	0.88	0.49	transposed
Mi_g024042	mi3:20135850	Mi_g027475	mi3:9565584	0.31	0.78	0.4	transposed

Mi_g019328	mi3:22458865	Mi_g015554	mi8:17772344	0.59	3.27	0.18	transposed
Mi_g031735	mi8:1645834	Mi_g007661	mi9:16429117	0.49	0.78	0.63	transposed
Mi_g001029	mi4:7969381	Mi_g004953	mi3:10197232	0.31	0.8	0.39	transposed
Mi_g008865	mi4:16700867	Mi_g003265	mi11:19469450	0.32	1.19	0.27	transposed
Mi_g030561	mi1:19908345	Mi_g003265	mi11:19469450	0.43	1.56	0.28	transposed
Mi_g000657	mi0:5211248	Mi_g008865	mi4:16700867	0.43	1.64	0.26	transposed
Mi_g000657	mi0:5211248	Mi_g003265	mi11:19469450	0.49	1.41	0.35	transposed
Mi_g016970	mi0:2003723	Mi_g003265	mi11:19469450	0.34	0.88	0.39	transposed
Mi_g016970	mi0:2003723	Mi_g030561	mi1:19908345	0.2	0.46	0.44	transposed
Mi_g009559	mi2:3681062	Mi_g003265	mi11:19469450	0.26	0.7	0.37	transposed
Mi_g008694	mi4:18602224	Mi_g032408	mi8:11654421	0.35	1.26	0.28	transposed
Mi_g018597	mi7:6581440	Mi_g003787	mi5:1143055	0.19	0.67	0.28	transposed
Mi_g029851	mi10:6619113	Mi_g003787	mi5:1143055	0.32	0.88	0.36	transposed
Mi_g023340	mi11:328232	Mi_g025554	mi5:2349713	0.28	1.76	0.16	transposed
Mi_g003034	mi6:3165444	Mi_g015554	mi8:17772344	0.54	1.39	0.39	transposed
Mi_g010355	mi9:20391316	Mi_g029488	mi3:5903872	0.52	1.77	0.29	transposed
Mi_g014262	mi6:4873899	Mi_g028484	mi7:10376079	0.45	1.1	0.41	transposed
Mi_g014574	mi10:8484141	Mi_g005585	mi3:7372820	0.61	1.73	0.35	transposed
Mi_g006309	mi2:2174562	Mi_g008329	mi9:18061682	0.3	1.25	0.24	transposed
Mi_g027397	mi10:3717910	Mi_g021477	mi6:25855352	0.18	0.4	0.46	transposed
Mi_g007306	mi10:6328467	Mi_g008562	mi6:29589580	0.24	1	0.24	transposed

Mi_g012642	mi7:387891	Mi_g032170	mi11:3017287	1.69	1.73	0.98	transposed
Mi_g029390	mi7:2063769	Mi_g012524	mi4:20374634	0.9	1.59	0.56	transposed
Mi_g029386	mi7:2121979	Mi_g023839	mi7:17954246	NA	NA	NA	transposed
Mi_g017465	mi7:9258956	Mi_g021090	mi3:1430597	0.2	0.7	0.29	transposed
Mi_g031010	mi7:15143966	Mi_g031435	mi2:11047508	0.31	1.22	0.26	transposed
Mi_g026997	mi8:19143224	Mi_g023093	mi6:1317584	0.23	0.54	0.43	transposed
Mi_g017587	mi6:24855615	Mi_g005275	mi9:9743648	0.36	1.14	0.31	transposed
Mi_g028992	mi7:20586062	Mi_g009254	mi4:1006715	0.35	0.76	0.46	transposed
Mi_g016662	mi1:3186783	Mi_g015962	mi1:4172748	0	0	NA	transposed
Mi_g020129	mi11:7480272	Mi_g031711	mi8:1909382	0.54	1.12	0.48	transposed
Mi_g017837	mi8:2204598	Mi_g015904	mi10:10346092	0.34	1.26	0.27	transposed
Mi_g028070	mi5:18104270	Mi_g001779	mi9:5988156	0.74	1.83	0.41	transposed
Mi_g004604	mi0:3142605	Mi_g021090	mi3:1430597	1.93	0.91	2.13	transposed
Mi_g021391	mi6:26662188	Mi_g029488	mi3:5903872	0.19	0.62	0.3	transposed
Mi_g026899	mi8:20063186	Mi_g010269	mi5:19846589	0.85	1.01	0.84	transposed
Mi_g028617	mi8:26314389	Mi_g031435	mi2:11047508	0.22	0.72	0.31	transposed
Mi_g001320	mi10:2931102	Mi_g008186	mi2:14623575	0.13	0.57	0.22	transposed
Mi_g005795	mi9:14634899	Mi_g016194	mi1:14372746	0.11	0.8	0.13	transposed
Mi_g005795	mi9:14634899	Mi_g019547	mi9:17320121	0.13	0.8	0.16	transposed
Mi_g020867	mi9:16885152	Mi_g016259	mi10:13791068	2.71	0.65	4.16	transposed
Mi_g019547	mi9:17320121	Mi_g002872	mi10:5275271	0.14	0.89	0.16	transposed

Mi_g005844	mi9:20962772	Mi_g008382	mi11:1209382	1.18	2.03	0.58	transposed
Mi_g005856	mi9:21138735	Mi_g005137	mi5:24410333	0.2	0.48	0.41	transposed
Mi_g001537	mi7:8642666	Mi_g026448	mi9:2484854	0.13	0.89	0.14	transposed
Mi_g006673	mi9:21747979	Mi_g014953	mi9:11347997	0.4	1.76	0.23	transposed
Mi_g003850	mi5:594008	Mi_g008186	mi2:14623575	0.12	0.58	0.21	transposed
Mi_g010294	mi5:19635296	Mi_g013025	mi7:15326570	0.76	1.16	0.66	transposed
Mi_g014228	mi6:5176608	Mi_g019901	mi6:20440835	0.21	0.87	0.24	transposed
Mi_g031699	mi2:13990664	Mi_g001538	mi7:8628712	NA	NA	NA	transposed

[a] The location referred to the first site of alignment on the chromosomes of *Musa itinerans* respectively
